# Supplementary material for: Improvement of Atopic Dermatitis by Synbiotic Baths
Source: Microorganisms. 2021 Mar 4;9(3):527. doi: 10.3390/microorganisms9030527 (PMC7998680; doi:10.3390/microorganisms9030527)
Supplement: Supplementary file 1 [file microorganisms-09-00527-s001.pdf]

*microorganisms*

MDPI

# Supplementary Materials: Improvement of Atopic Dermatitis by Synbiotic Baths

**Matthias Noll [1,*], Michael Jäger [1], Leonie Lux [1], Christian Buettner [1] and Michaela Axt-Gadermann [1]**

[1] Coburg University of Applied Sciences and Arts, Institute for Bioanalysis, Friedrich-Streib-Str. 2, 96450 Coburg, Germany

* Contact information of the corresponding author. Phone: +49-9561-317645, Fax: +49-9561-317346,

E-mail: matthias.noll@hs-coburg.de

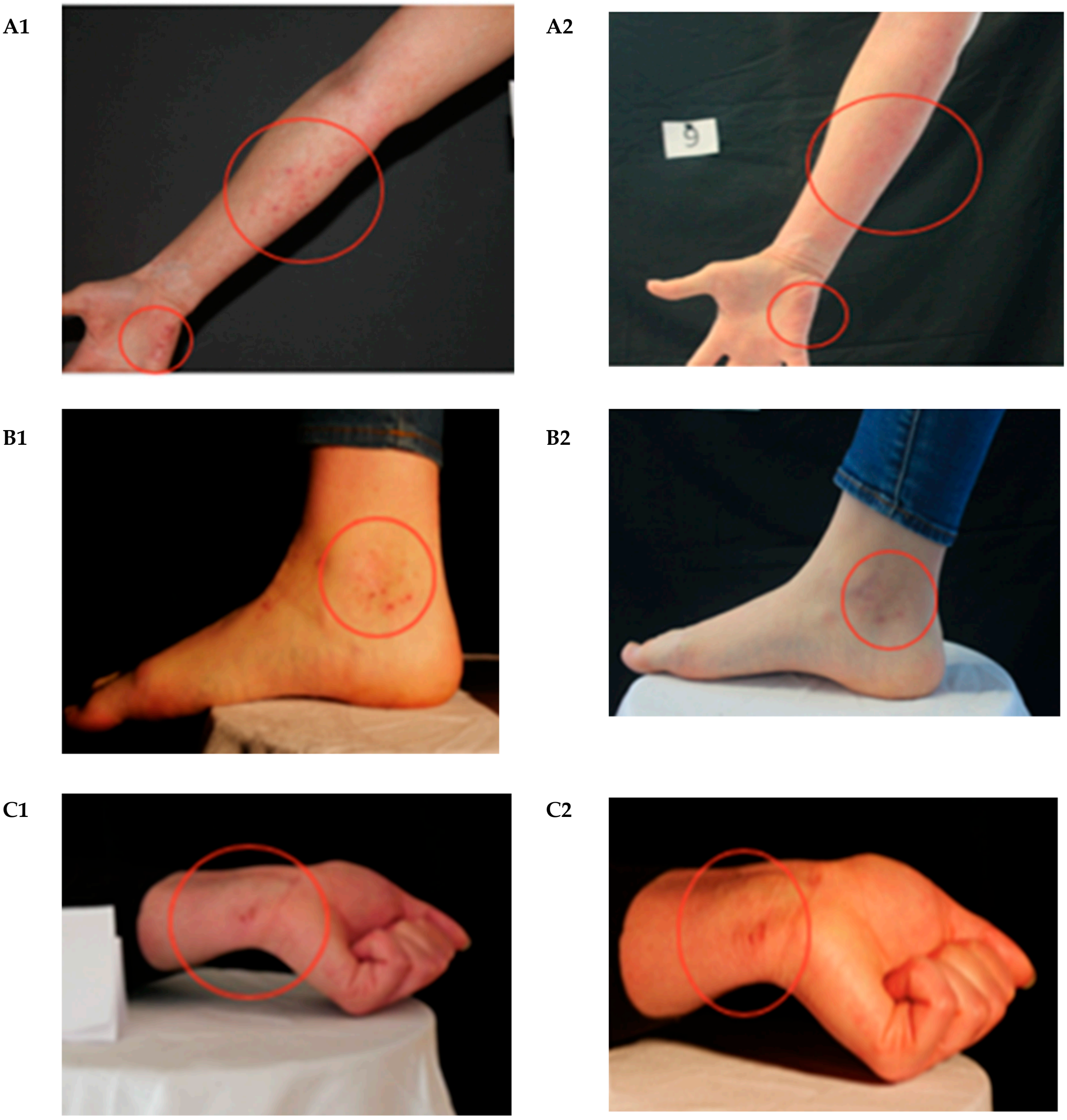


**Figure S1.** Changes in skin appearance from AD patients after daily snybiotic baths (**A**), prebiotic baths (**B**) or placebo baths (**C**) over time (start of incubation = 1; after 14 days of bath treatment = 2).

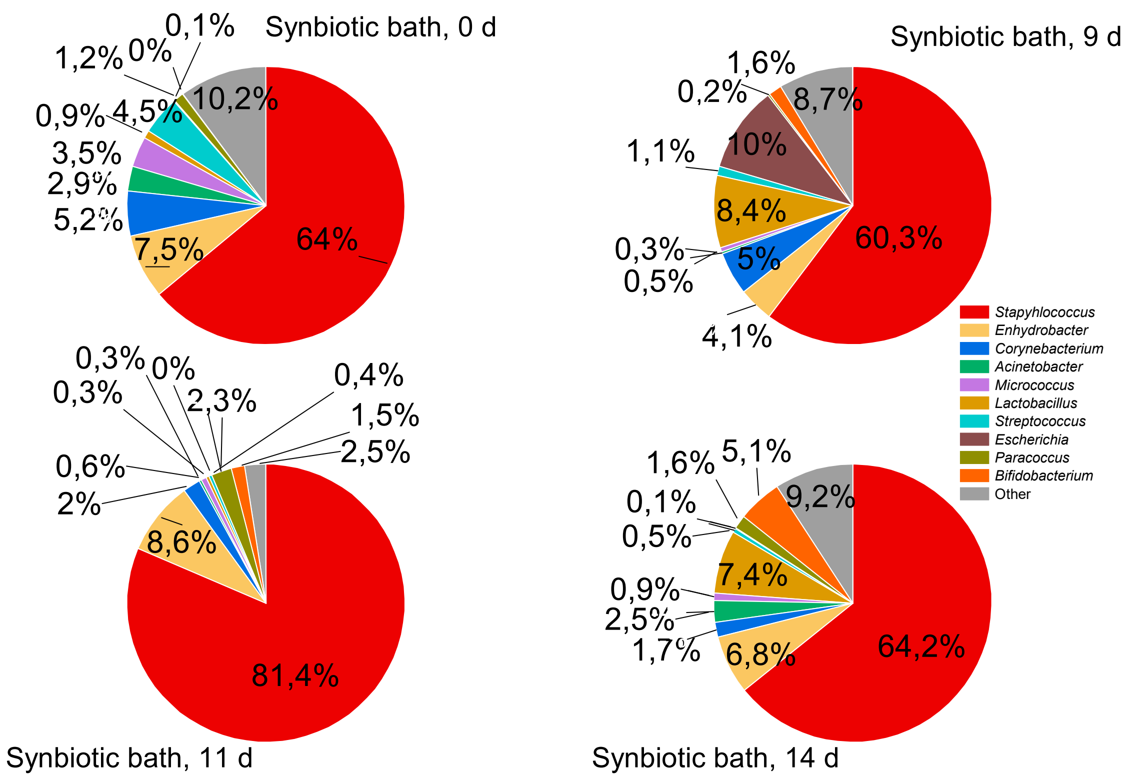
Synbiotic bath, 0 d
64%
7,5%
5,2%
2,9%
3,5%
0,9%
4,5%
1,2%
0%
0,1%
10,2%
Synbiotic bath, 9 d
60,3%
4,1%
0,5%
0,3%
5%
8,4%
1,1%
10%
0,2%
1,6%
8,7%
Stapyhlococcus
Enhydrobacter
Corynebacterium
Acinetobacter
Micrococcus
Lactobacillus
Streptococcus
Escherichia
Paracoccus
Bifidobacterium
Other
Synbiotic bath, 11 d
81,4%
8,6%
2%
0,6%
0,3%
0,3%
0%
2,3%
0,4%
1,5%
2,5%
Synbiotic bath, 14 d
64,2%
6,8%
1,7%
2,5%
0,9%
7,4%
0,5%
0,1%
1,6%
5,1%
9,2%

**A**

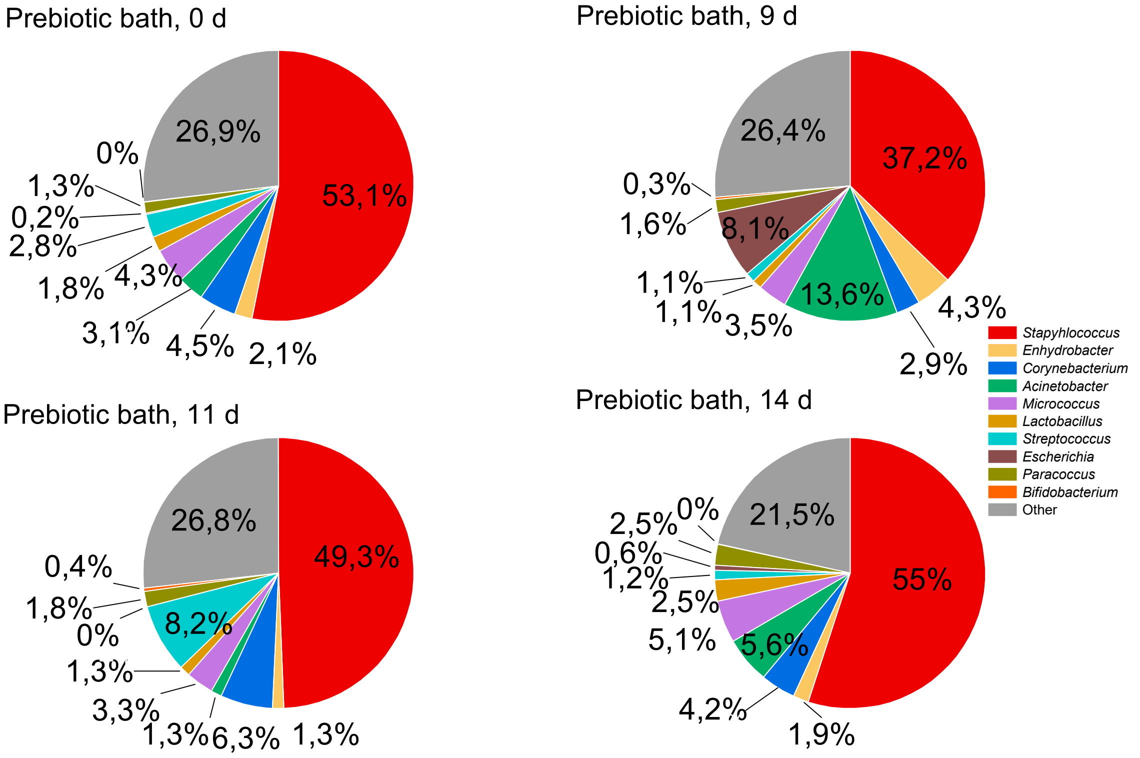
Prebiotic bath, 0 d
53,1%
2,1%
4,5%
3,1%
1,8%
4,3%
2,8%
0,2%
1,3%
0%
26,9%
Prebiotic bath, 9 d
37,2%
4,3%
2,9%
13,6%
3,5%
1,1%
1,1%
8,1%
1,6%
0,3%
26,4%
Stapyhlococcus
Enhydrobacter
Corynebacterium
Acinetobacter
Micrococcus
Lactobacillus
Streptococcus
Escherichia
Paracoccus
Bifidobacterium
Other
Prebiotic bath, 11 d
49,3%
1,3%
6,3%
1,3%
3,3%
1,3%
0%
8,2%
1,8%
0,4%
26,8%
Prebiotic bath, 14 d
55%
1,9%
4,2%
5,6%
5,1%
2,5%
1,2%
0,6%
2,5%
0%
21,5%

**B**

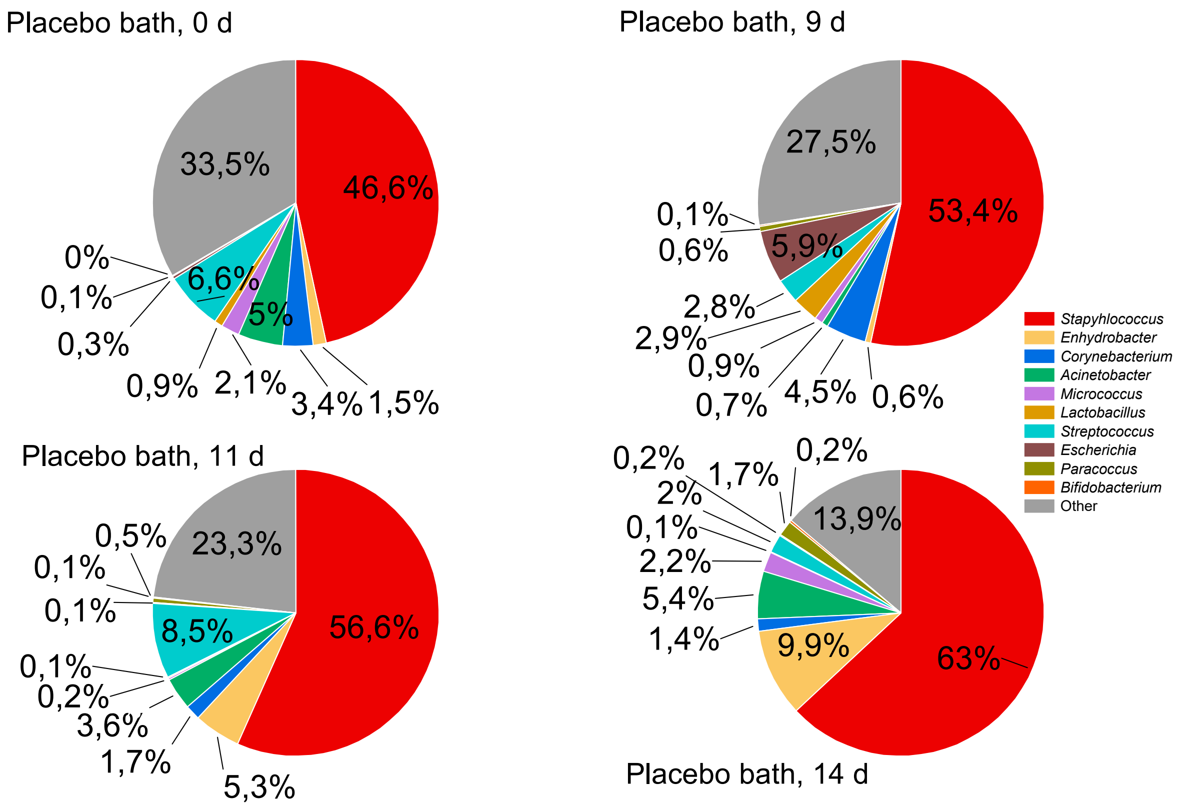


**C**

**Figure S2.** Relative sequence read abundances of the bacterial skin microbiome retrieved from AD patients after daily snybiotic baths (**A**), prebiotic baths (**B**) or placebo baths (**C**) over time.

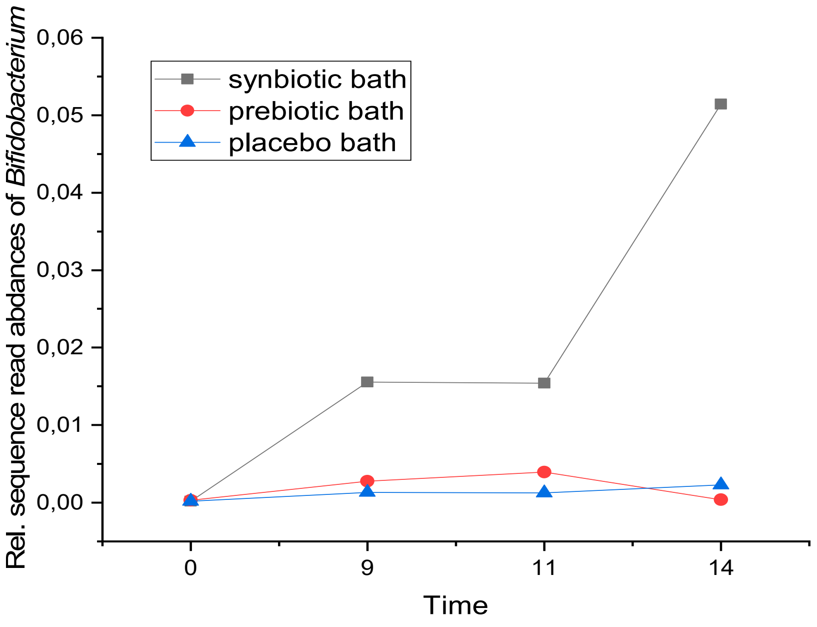


**A**

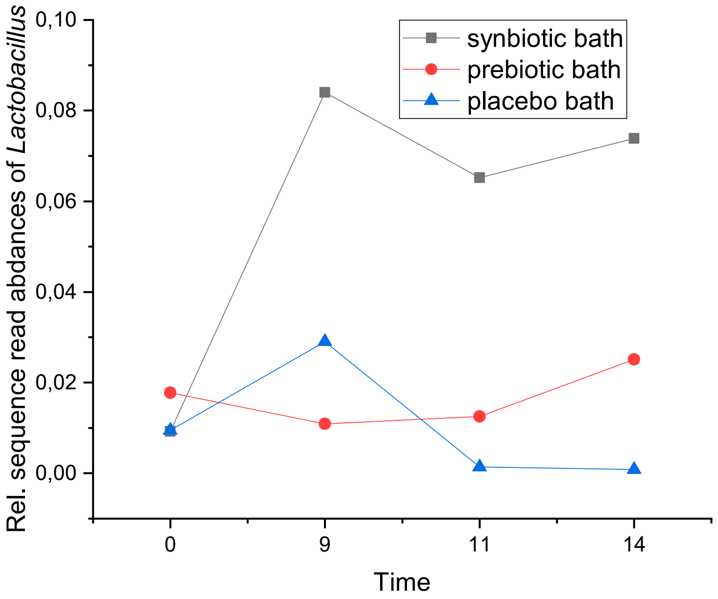


**B**

**Figure S3.** Relative sequence read abundances of members of the genera *Bifidobacterium* (**A**) and *Lactobacillus* (**B**) as part of the bacterial skin microbiome retrieved from AD patients after daily synbiotic, prebiotic or placebo baths over time.

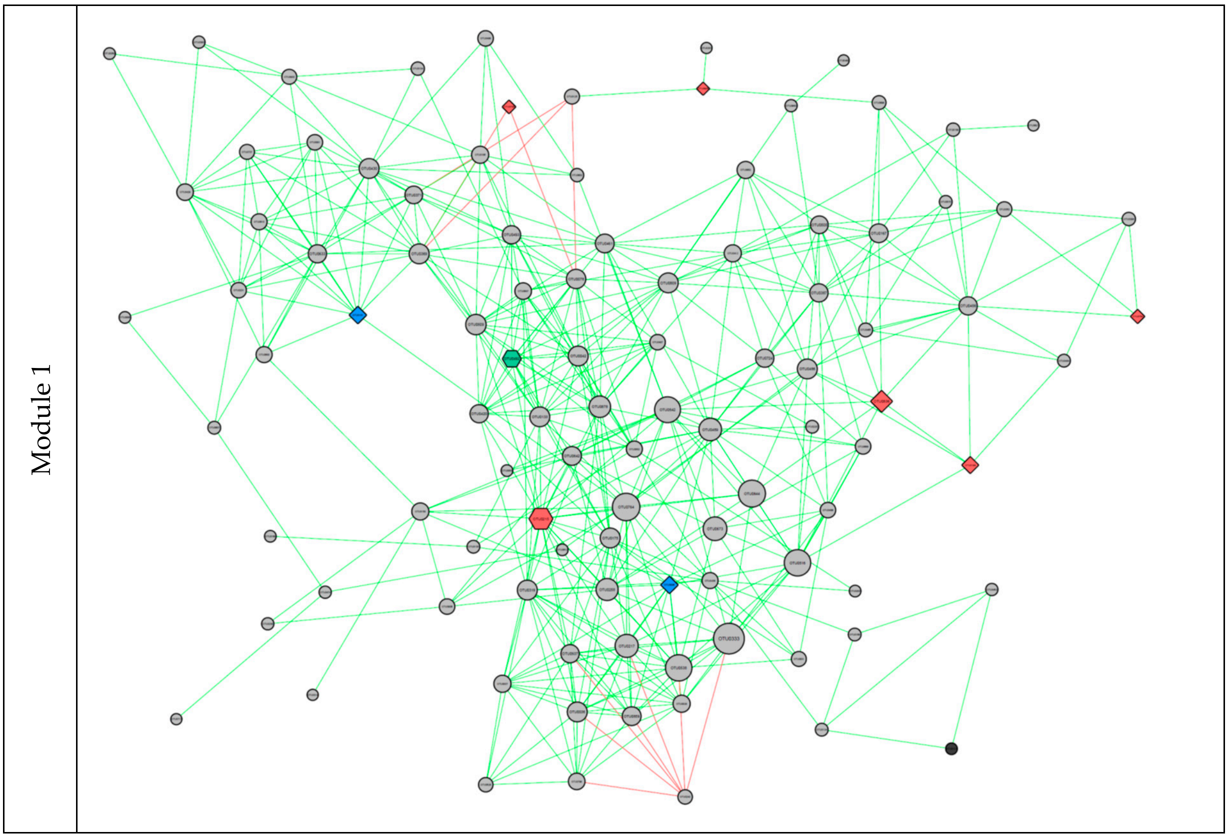

| | |
|---|---|
| Module 2 | 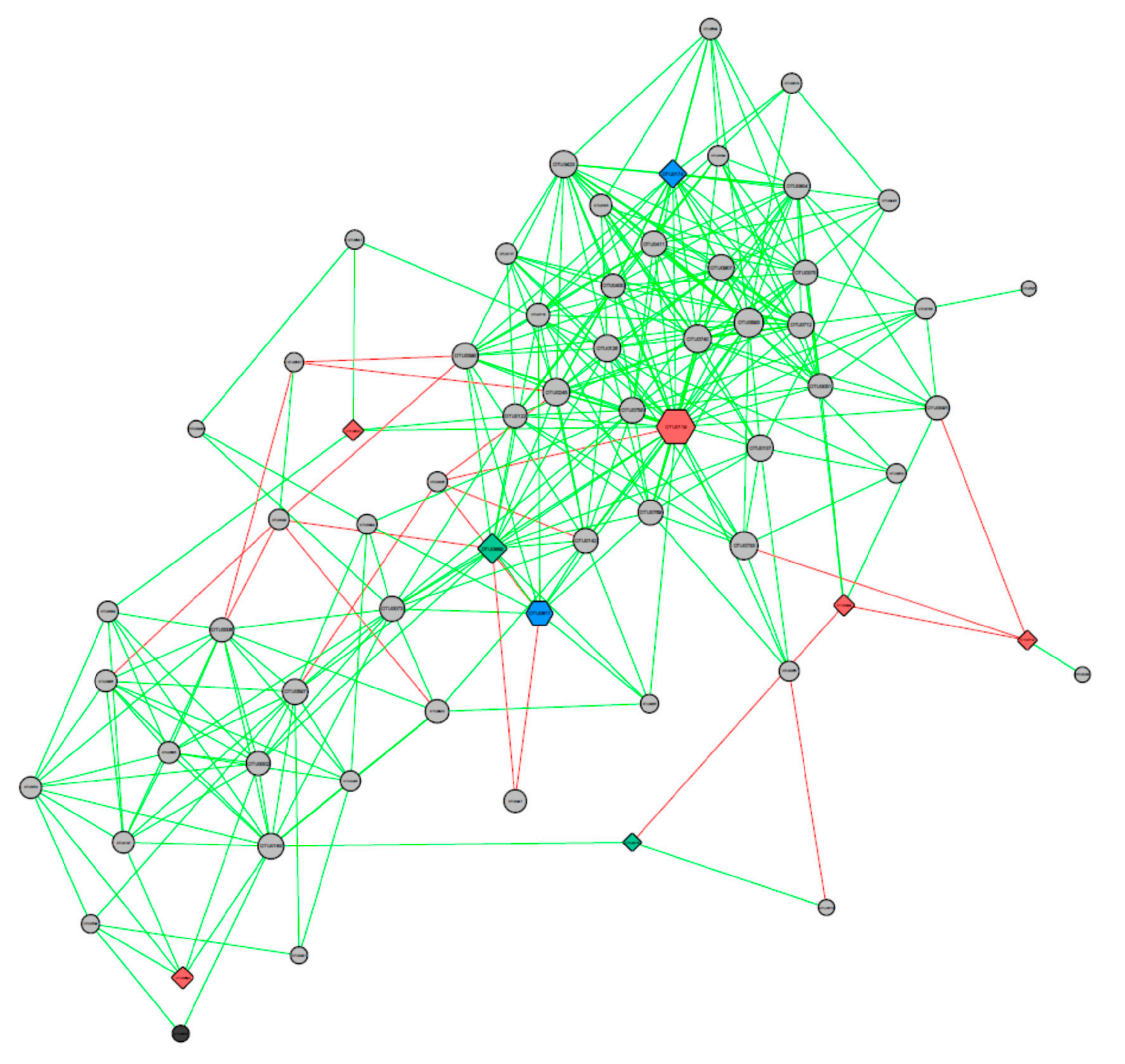 |

Module 3

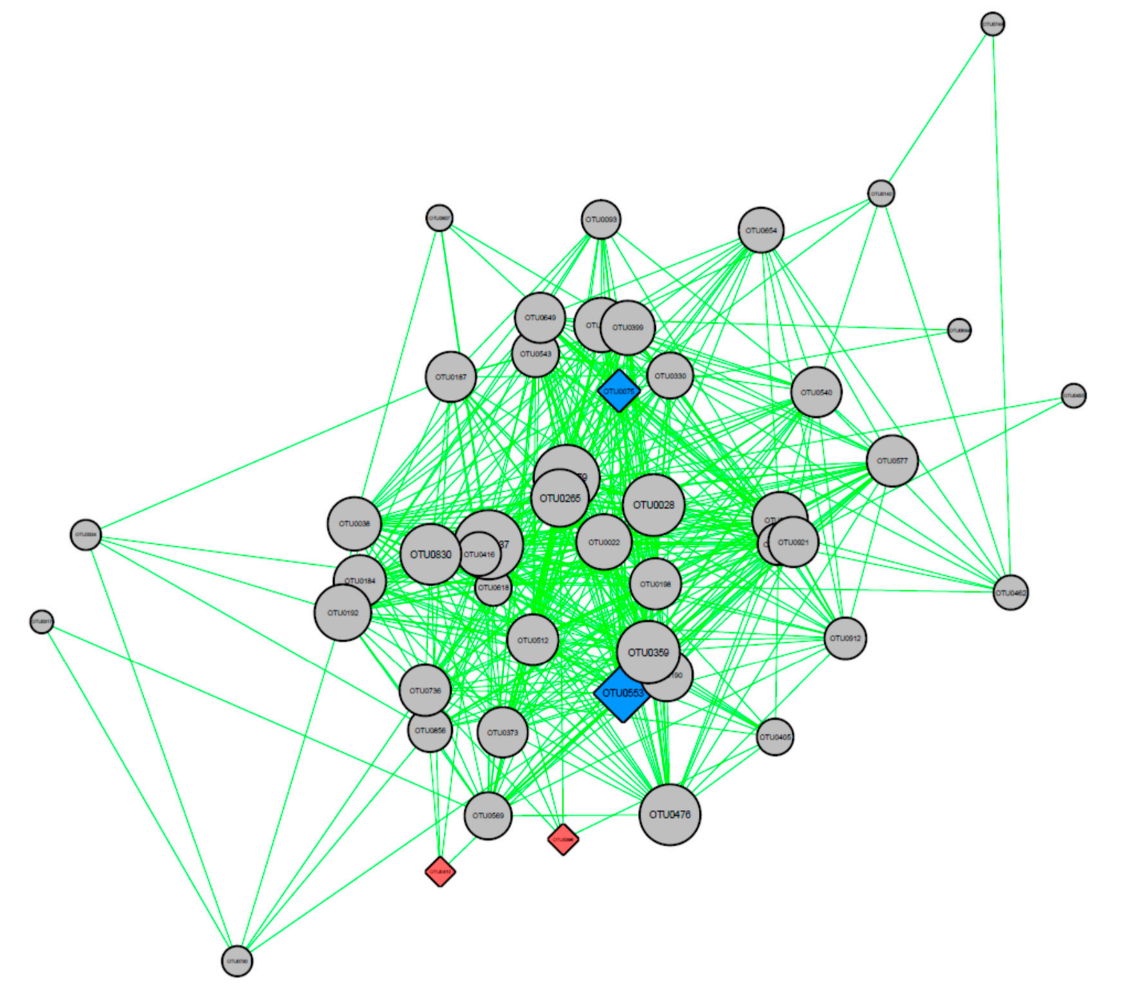


Module 4

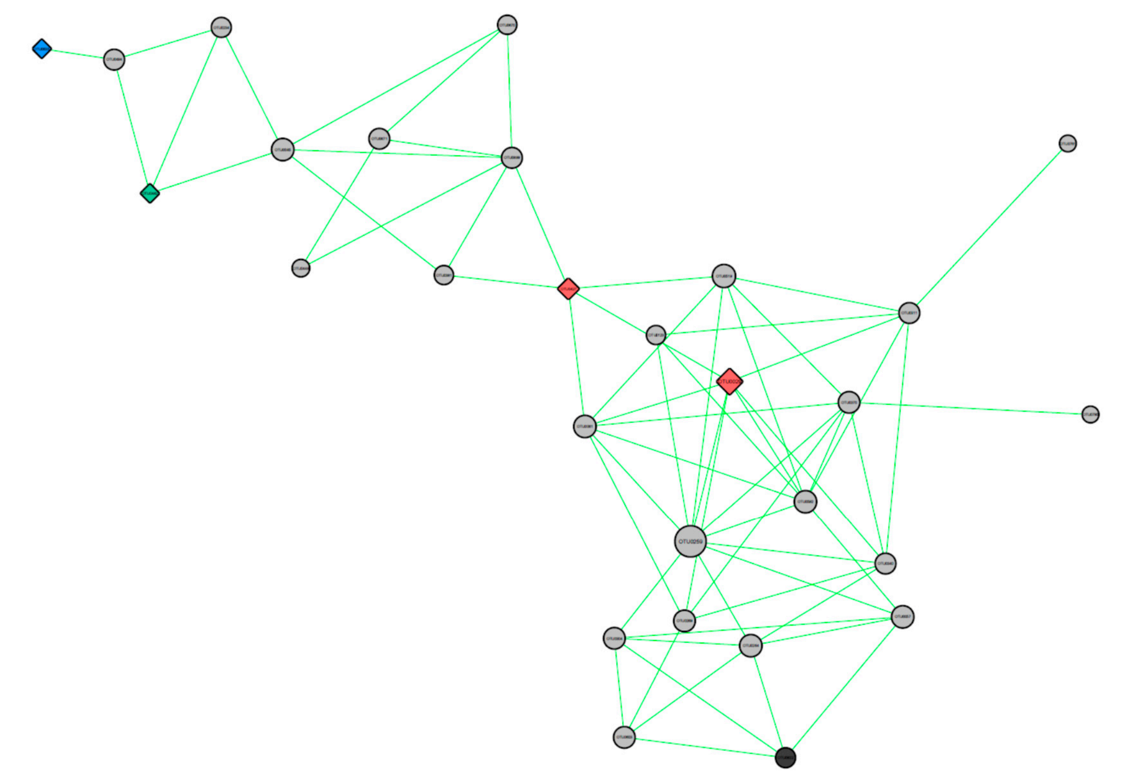

| | |
|---|---|
| Module 5 | 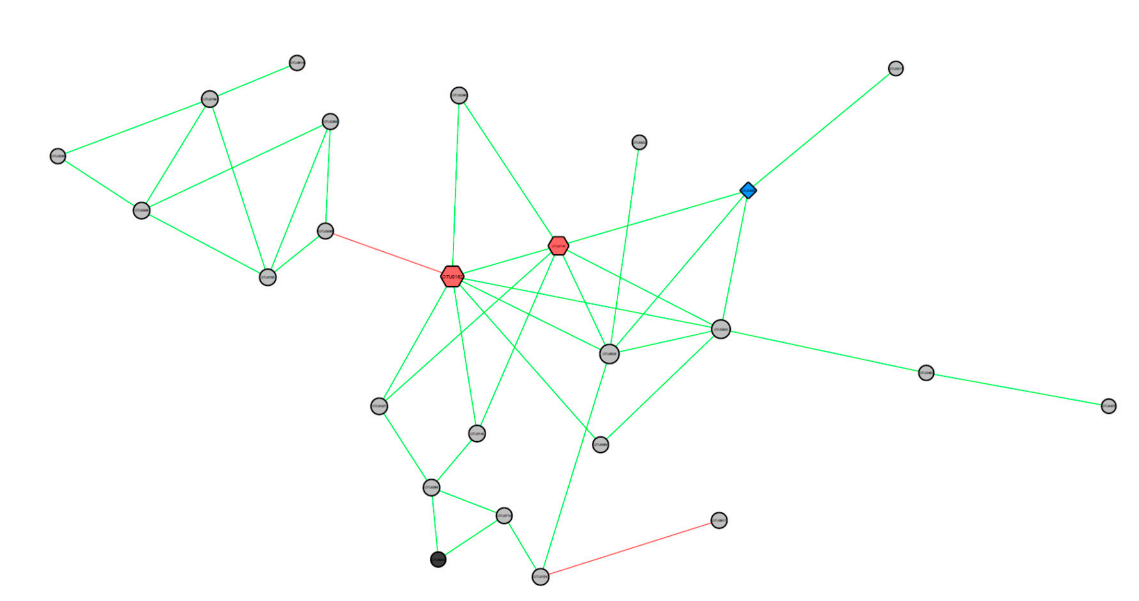 |
| Module 6 | 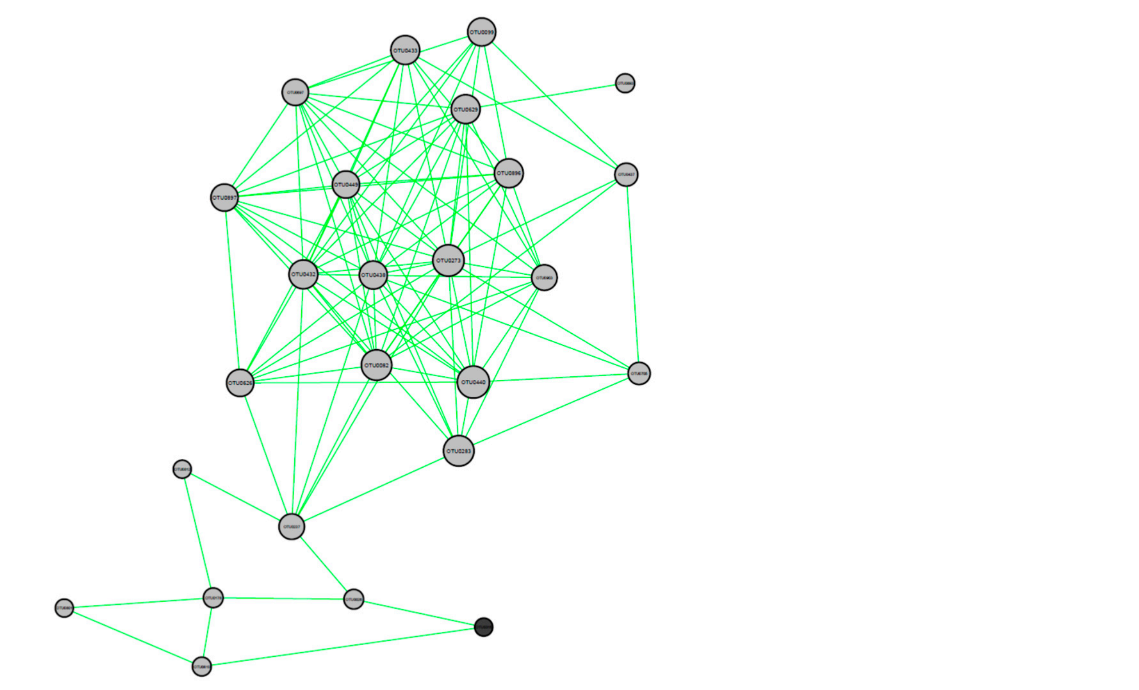 |

| | |
|---|---|
| Module 7 | 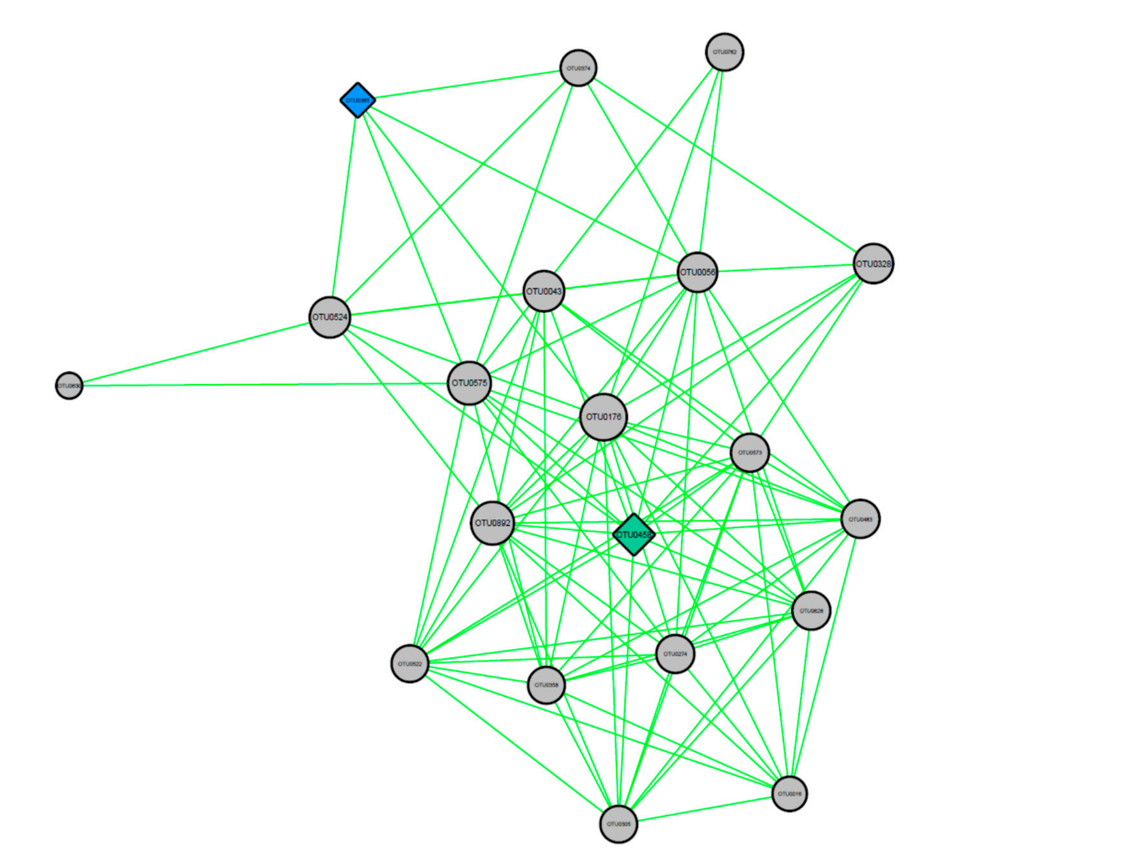  |
| Module 8 | 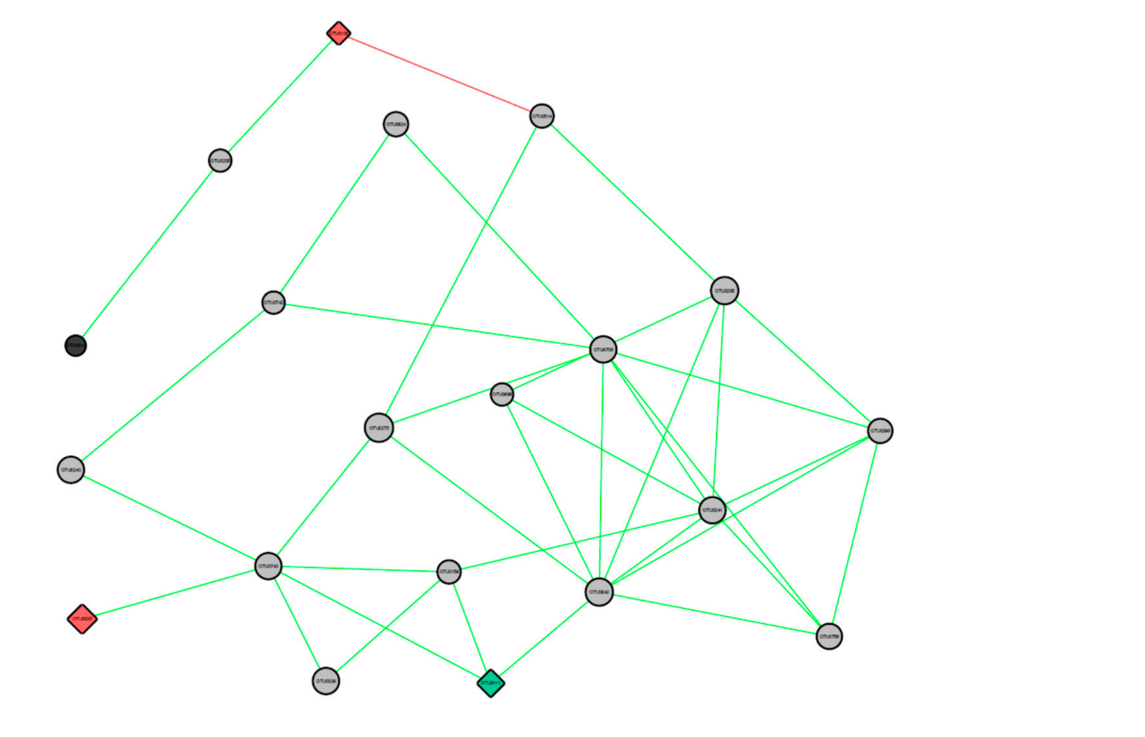 |

Module 9 and 10

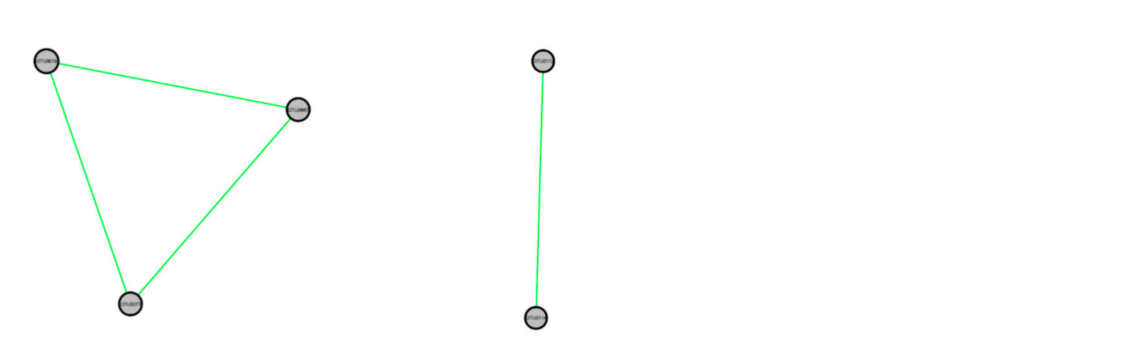

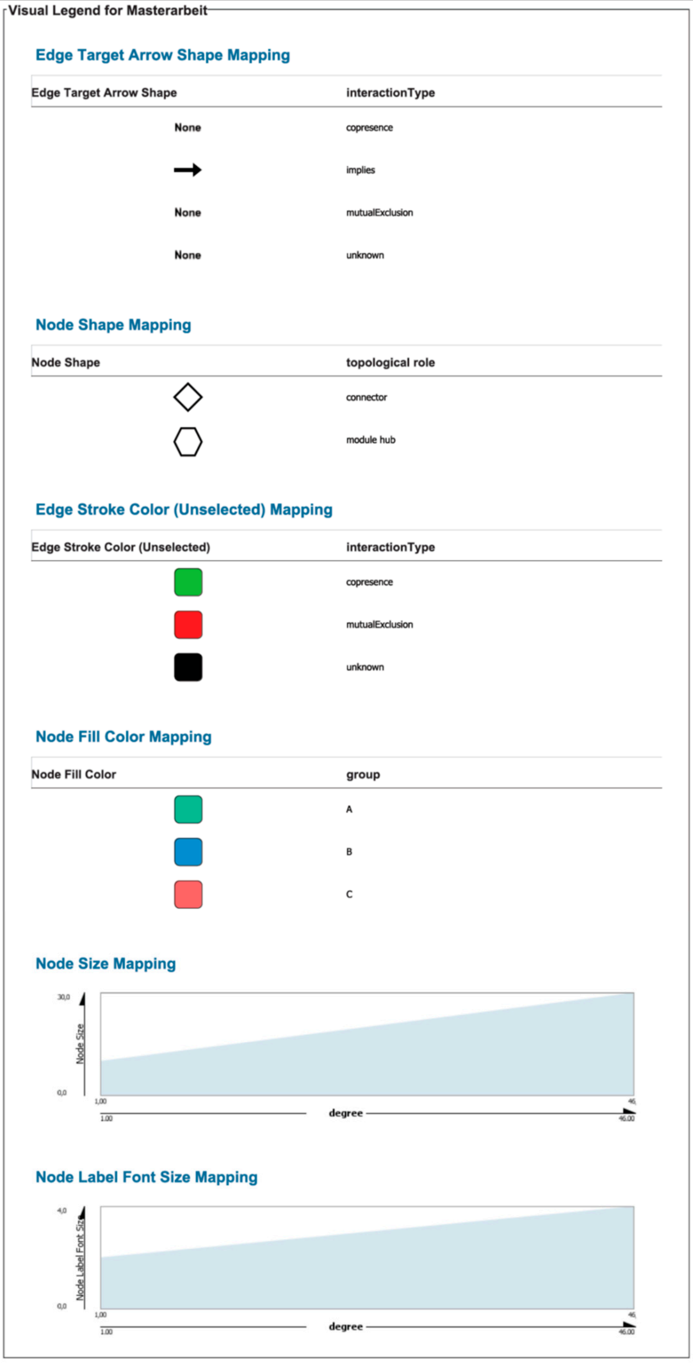


**Figure S4.** Co-occurence network from the differences of the bacterial skin microbiome networks retrieved from AD patients after daily synbiotic, prebiotic or placebo baths. In total, ten different module were found. Further details can be found in the material and methods section in the main document.

**Table S1.** Two way analysis of variance (ANOVA) of diversity indices from the bacterial skin microbiome derived from AD patients that attended synbiotic, prebiotic or placebo baths over time. Significance of bath treatment, time or the interaction of bath treatment and time is highlighted in **bold** ($p < 0.05$). DF, degree of freedom.

| Parameter | DF | Sum of Squares | Mean Square | F Value | P Value |
|---|---|---|---|---|---|
| *Chao1 richness estimator* | | | | | |
| **Time** | 3 | 942,634,121 | 314,211,374 | 0,98478 | 0,40542 |
| **Bath treatment** | 2 | 1,162,547,775 | 581,273,888 | 182,179 | 0,16978 |
| **Bath treatment x Time** | 6 | 813,839,017 | 135,639,836 | 0,42511 | 0,8597 |
| *Shannon diversity index* | | | | | |
| **Time** | 3 | 451,465 | 150,488 | 164,409 | 0,18766 |
| **Bath treatment** | **2** | **1,096,197** | **548,098** | **598,802** | **0,00408** |
| **Bath treatment x Time** | 6 | 245,948 | 0,40991 | 0,44783 | 0,84397 |
| *Simpson diversity index* | | | | | |
| **Time** | 3 | 0,19093 | 0,06364 | 0,71964 | 0,5438 |
| **Bath treatment** | **2** | **0,71126** | **0,35563** | **402,137** | **0,02249** |

**Table S2.** Topological role of OTUs in co-occurence networks derived from AD patients that attended synbiotic (A), prebiotic (B) or placebo baths and a merged network with the differences in the topological role between all three bath treatments (D).

**A** Synbiotic bath treatment:

| Node | Module | Edges All | Edges Inside Module | Edges Outside Module | Zi (within) | pi(outside) | Role |
|---|---|---|---|---|---|---|---|
| OTU0896 | 2 | 6 | 6 | 0 | -0,18544571 | 0 | Peripherals |
| OTU0124 | 2 | 7 | 6 | 1 | -0,18544571 | 0,24489796 | Peripherals |
| OTU0575 | 3 | 34 | 33 | 1 | 0,28510912 | 0,05709343 | Peripherals |
| OTU0093 | 3 | 52 | 51 | 1 | 1,3331102 | 0,03772189 | Peripherals |
| OTU0519 | 1 | 40 | 11 | 29 | 0,53080423 | 0,4675 | Peripherals |
| OTU0091 | 1 | 17 | 13 | 4 | 0,88467372 | 0,38062284 | Peripherals |
| OTU0126 | 5 | 22 | 21 | 1 | 0,81155763 | 0,08677686 | Peripherals |
| OTU0220 | 5 | 20 | 20 | 0 | 0,67923845 | 0 | Peripherals |
| OTU0795 | 3 | 55 | 53 | 2 | 1,44955476 | 0,0707438 | Peripherals |
| OTU0061 | 3 | 54 | 40 | 14 | 0,69266509 | 0,40672154 | Peripherals |
| OTU0684 | 3 | 48 | 48 | 0 | 1,15844335 | 0 | Peripherals |
| OTU0807 | 3 | 35 | 34 | 1 | 0,3433314 | 0,0555102 | Peripherals |
| OTU0730 | 3 | 60 | 57 | 3 | 1,68244389 | 0,09611111 | Peripherals |
| OTU0022 | 3 | 59 | 52 | 7 | 1,39133248 | 0,21488078 | Peripherals |
| OTU0176 | 6 | 7 | 5 | 2 | 0,07233642 | 0,40816327 | Peripherals |
| OTU0050 | 6 | 6 | 6 | 0 | 0,38579426 | 0 | Peripherals |
| OTU0383 | 3 | 56 | 53 | 3 | 1,44955476 | 0,10267857 | Peripherals |
| OTU0122 | 1 | 45 | 14 | 31 | 1,06160846 | 0,4582716 | Peripherals |
| OTU0150 | 3 | 43 | 30 | 13 | 0,11044227 | 0,43482964 | Peripherals |
| OTU0381 | 3 | 44 | 43 | 1 | 0,86733194 | 0,04442149 | Peripherals |
| OTU0729 | 1 | 24 | 9 | 15 | 0,17693474 | 0,66319444 | Connector |
| OTU0074 | 5 | 19 | 16 | 3 | 0,14996174 | 0,28254848 | Peripherals |

| | | | | | | | |
|---|---|---|---|---|---|---|---|
| OTU0830 | 3 | 26 | 24 | 2 | -0,23889143 | 0,14201183 | Peripherals |
| OTU0736 | 1 | 26 | 11 | 15 | 0,53080423 | 0,59467456 | Peripherals |
| OTU0399 | 3 | 53 | 51 | 2 | 1,3331102 | 0,07333571 | Peripherals |
| OTU0822 | 3 | 35 | 32 | 3 | 0,22688683 | 0,16 | Peripherals |
| OTU0733 | 5 | 19 | 19 | 0 | 0,54691927 | 0 | Peripherals |
| OTU0659 | 5 | 17 | 17 | 0 | 0,28228092 | 0 | Peripherals |
| OTU0201 | 5 | 18 | 18 | 0 | 0,41460009 | 0 | Peripherals |
| OTU0761 | 5 | 20 | 20 | 0 | 0,67923845 | 0 | Peripherals |
| OTU0148 | 5 | 21 | 20 | 1 | 0,67923845 | 0,09070295 | Peripherals |
| OTU0491 | 2 | 15 | 9 | 6 | 0,68383107 | 0,52444444 | Peripherals |
| OTU0458 | 1 | 5 | 2 | 3 | -1,06160846 | 0,64 | Connector |
| OTU0748 | 3 | 42 | 41 | 1 | 0,75088737 | 0,04648526 | Peripherals |
| OTU0588 | 3 | 34 | 34 | 0 | 0,3433314 | 0 | Peripherals |
| OTU0516 | 3 | 50 | 43 | 7 | 0,86733194 | 0,2496 | Peripherals |
| OTU0014 | 2 | 15 | 13 | 2 | 1,84286678 | 0,24 | Peripherals |
| OTU0295 | 2 | 11 | 11 | 0 | 1,26334893 | 0 | Peripherals |
| OTU0294 | 2 | 8 | 8 | 0 | 0,39407214 | 0 | Peripherals |
| OTU0513 | 3 | 38 | 38 | 0 | 0,57622053 | 0 | Peripherals |
| OTU0274 | 3 | 49 | 49 | 0 | 1,21666563 | 0 | Peripherals |
| OTU0192 | 3 | 59 | 53 | 6 | 1,44955476 | 0,18557886 | Peripherals |
| OTU0484 | 3 | 33 | 33 | 0 | 0,28510912 | 0 | Peripherals |
| OTU0190 | 3 | 51 | 50 | 1 | 1,27488792 | 0,03844675 | Peripherals |
| OTU0817 | 5 | 22 | 21 | 1 | 0,81155763 | 0,08677686 | Peripherals |
| OTU0933 | 5 | 8 | 7 | 1 | -1,04091087 | 0,21875 | Peripherals |
| OTU0667 | 2 | 14 | 14 | 0 | 2,13262571 | 0 | Peripherals |
| OTU0932 | 2 | 15 | 11 | 4 | 1,26334893 | 0,39111111 | Peripherals |
| OTU0051 | 3 | 46 | 41 | 5 | 0,75088737 | 0,19754253 | Peripherals |
| OTU0572 | 5 | 16 | 16 | 0 | 0,14996174 | 0 | Peripherals |
| OTU0573 | 2 | 2 | 1 | 1 | -1,63424035 | 0,5 | Peripherals |
| OTU0669 | 3 | 36 | 35 | 1 | 0,40155368 | 0,05401235 | Peripherals |
| OTU0039 | 5 | 25 | 20 | 5 | 0,67923845 | 0,3424 | Peripherals |
| OTU0856 | 3 | 39 | 39 | 0 | 0,63444281 | 0 | Peripherals |
| OTU0142 | 3 | 53 | 49 | 4 | 1,21666563 | 0,14168743 | Peripherals |
| OTU0149 | 3 | 45 | 36 | 9 | 0,45977596 | 0,32790123 | Peripherals |
| OTU0198 | 1 | 32 | 17 | 15 | 1,59241269 | 0,52539063 | Peripherals |
| OTU0895 | 3 | 9 | 7 | 2 | -1,22867023 | 0,34567901 | Peripherals |
| OTU0762 | 3 | 9 | 7 | 2 | -1,22867023 | 0,37037037 | Peripherals |
| OTU0892 | 3 | 7 | 4 | 3 | -1,40333708 | 0,48979592 | Peripherals |
| OTU0043 | 3 | 10 | 8 | 2 | -1,17044795 | 0,34 | Peripherals |
| OTU0728 | 3 | 14 | 13 | 1 | -0,87933653 | 0,13265306 | Peripherals |
| OTU0423 | 3 | 57 | 53 | 4 | 1,44955476 | 0,13357956 | Peripherals |
| OTU0002 | 3 | 9 | 7 | 2 | -1,22867023 | 0,37037037 | Peripherals |

| | | | | | | | |
|---|---|---|---|---|---|---|---|
| OTU0889 | 3 | 21 | 10 | 11 | -1,05400338 | 0,67120181 | Connector |
| OTU0203 | 3 | 40 | 40 | 0 | 0,69266509 | 0 | Peripherals |
| OTU0069 | 3 | 41 | 36 | 5 | 0,45977596 | 0,21891731 | Peripherals |
| OTU0525 | 3 | 13 | 13 | 0 | -0,87933653 | 0 | Peripherals |
| OTU0049 | 3 | 20 | 17 | 3 | -0,6464474 | 0,27 | Peripherals |
| OTU0714 | 6 | 5 | 1 | 4 | -1,18149491 | 0,32 | Peripherals |
| OTU0330 | 5 | 2 | 2 | 0 | -1,70250677 | 0 | Peripherals |
| OTU0624 | 5 | 16 | 15 | 1 | 0,01764256 | 0,1171875 | Peripherals |
| OTU0387 | 3 | 39 | 36 | 3 | 0,45977596 | 0,14464168 | Peripherals |
| OTU0412 | 3 | 40 | 40 | 0 | 0,69266509 | 0 | Peripherals |
| OTU0258 | 3 | 59 | 53 | 6 | 1,44955476 | 0,18557886 | Peripherals |
| OTU0737 | 1 | 4 | 3 | 1 | -0,88467372 | 0,375 | Peripherals |
| OTU0712 | 1 | 3 | 2 | 1 | -1,06160846 | 0,44444444 | Peripherals |
| OTU0007 | 3 | 27 | 23 | 4 | -0,29711371 | 0,260631 | Peripherals |
| OTU0217 | 3 | 52 | 48 | 4 | 1,15844335 | 0,14423077 | Peripherals |
| OTU0183 | 3 | 51 | 49 | 2 | 1,21666563 | 0,07612457 | Peripherals |
| OTU0365 | 1 | 4 | 4 | 0 | -0,70773897 | 0 | Peripherals |
| OTU0483 | 2 | 11 | 9 | 2 | 0,68383107 | 0,29752066 | Peripherals |
| OTU0019 | 2 | 8 | 8 | 0 | 0,39407214 | 0 | Peripherals |
| OTU0486 | 3 | 48 | 41 | 7 | 0,75088737 | 0,25954861 | Peripherals |
| OTU0675 | 3 | 19 | 17 | 2 | -0,6464474 | 0,18836565 | Peripherals |
| OTU0182 | 3 | 38 | 37 | 1 | 0,51799824 | 0,05124654 | Peripherals |
| OTU0455 | 2 | 11 | 11 | 0 | 1,26334893 | 0 | Peripherals |
| OTU0181 | 3 | 29 | 26 | 3 | -0,12244686 | 0,1902497 | Peripherals |
| OTU0125 | 3 | 18 | 18 | 0 | -0,58822512 | 0 | Peripherals |
| OTU0644 | 3 | 51 | 50 | 1 | 1,27488792 | 0,03844675 | Peripherals |
| OTU0100 | 3 | 61 | 57 | 4 | 1,68244389 | 0,12416017 | Peripherals |
| OTU0276 | 2 | 21 | 4 | 17 | -0,76496357 | 0,6893424 | Connector |
| OTU0564 | 3 | 56 | 48 | 8 | 1,15844335 | 0,24936224 | Peripherals |
| OTU0107 | 5 | 22 | 22 | 0 | 0,94387681 | 0 | Peripherals |
| OTU0570 | 3 | 62 | 58 | 4 | 1,74066618 | 0,12330905 | Peripherals |
| OTU0373 | 5 | 21 | 21 | 0 | 0,81155763 | 0 | Peripherals |
| OTU0410 | 3 | 48 | 42 | 6 | 0,80910966 | 0,21875 | Peripherals |
| OTU0240 | 6 | 4 | 4 | 0 | -0,24112141 | 0 | Peripherals |
| OTU0328 | 6 | 6 | 5 | 1 | 0,07233642 | 0,27777778 | Peripherals |
| OTU0379 | 2 | 43 | 5 | 38 | -0,47520464 | 0,52677123 | Peripherals |
| OTU0333 | 3 | 49 | 48 | 1 | 1,15844335 | 0,03998334 | Peripherals |
| OTU0543 | 3 | 29 | 20 | 9 | -0,47178056 | 0,49940547 | Peripherals |
| OTU0424 | 1 | 12 | 12 | 0 | 0,70773897 | 0 | Peripherals |
| OTU0086 | 1 | 14 | 12 | 2 | 0,70773897 | 0,25510204 | Peripherals |
| OTU0108 | 5 | 20 | 20 | 0 | 0,67923845 | 0 | Peripherals |
| OTU0175 | 1 | 25 | 14 | 11 | 1,06160846 | 0,5248 | Peripherals |

| | | | | | | | |
|---|---|---|---|---|---|---|---|
| OTU0755 | 1 | 9 | 9 | 0 | 0,17693474 | 0 | Peripherals |
| OTU0540 | 1 | 15 | 15 | 0 | 1,2385432 | 0 | Peripherals |
| OTU0184 | 5 | 19 | 19 | 0 | 0,54691927 | 0 | Peripherals |
| OTU0041 | 1 | 26 | 18 | 8 | 1,76934743 | 0,44674556 | Peripherals |
| OTU0535 | 3 | 25 | 19 | 6 | -0,53000284 | 0,3648 | Peripherals |
| OTU0314 | 2 | 8 | 6 | 2 | -0,18544571 | 0,375 | Peripherals |
| OTU0930 | 3 | 10 | 9 | 1 | -1,11222566 | 0,18 | Peripherals |
| OTU0597 | 3 | 27 | 26 | 1 | -0,12244686 | 0,07133059 | Peripherals |
| OTU0592 | 3 | 25 | 25 | 0 | -0,18066914 | 0 | Peripherals |
| OTU0836 | 3 | 19 | 17 | 2 | -0,6464474 | 0,19390582 | Peripherals |
| OTU0893 | 2 | 5 | 5 | 0 | -0,47520464 | 0 | Peripherals |
| OTU0082 | 2 | 10 | 10 | 0 | 0,97359 | 0 | Peripherals |
| OTU0542 | 3 | 58 | 55 | 3 | 1,56599933 | 0,09928656 | Peripherals |
| OTU0159 | 3 | 13 | 12 | 1 | -0,93755882 | 0,14201183 | Peripherals |
| OTU0593 | 6 | 2 | 1 | 1 | -1,18149491 | 0,5 | Peripherals |
| OTU0111 | 5 | 12 | 5 | 7 | -1,30554923 | 0,63888889 | Connector |
| OTU0692 | 5 | 22 | 22 | 0 | 0,94387681 | 0 | Peripherals |
| OTU0880 | 3 | 18 | 16 | 2 | -0,70466969 | 0,2037037 | Peripherals |
| OTU0492 | 6 | 15 | 13 | 2 | 2,5799991 | 0,23111111 | Module Hub |
| OTU0705 | 6 | 8 | 7 | 1 | 0,69925209 | 0,21875 | Peripherals |
| OTU0605 | 3 | 18 | 14 | 4 | -0,82111425 | 0,37654321 | Peripherals |
| OTU0342 | 2 | 4 | 4 | 0 | -0,76496357 | 0 | Peripherals |
| OTU0438 | 2 | 4 | 4 | 0 | -0,76496357 | 0 | Peripherals |
| OTU0528 | 5 | 22 | 22 | 0 | 0,94387681 | 0 | Peripherals |
| OTU0885 | 1 | 22 | 17 | 5 | 1,59241269 | 0,3677686 | Peripherals |
| OTU0629 | 2 | 11 | 11 | 0 | 1,26334893 | 0 | Peripherals |
| OTU0903 | 2 | 9 | 9 | 0 | 0,68383107 | 0 | Peripherals |
| OTU0140 | 3 | 38 | 37 | 1 | 0,51799824 | 0,05124654 | Peripherals |
| OTU0482 | 2 | 13 | 10 | 3 | 0,97359 | 0,35502959 | Peripherals |
| OTU0242 | 5 | 21 | 21 | 0 | 0,81155763 | 0 | Peripherals |
| OTU0626 | 2 | 6 | 6 | 0 | -0,18544571 | 0 | Peripherals |
| OTU0489 | 3 | 40 | 35 | 5 | 0,40155368 | 0,2275 | Peripherals |
| OTU0745 | 3 | 32 | 27 | 5 | -0,06422458 | 0,26367188 | Peripherals |
| OTU0509 | 2 | 8 | 5 | 3 | -0,47520464 | 0,53125 | Peripherals |
| OTU0494 | 5 | 6 | 4 | 2 | -1,43786841 | 0,44444444 | Peripherals |
| OTU0665 | 3 | 27 | 20 | 7 | -0,47178056 | 0,41152263 | Peripherals |
| OTU0663 | 3 | 24 | 20 | 4 | -0,47178056 | 0,29166667 | Peripherals |
| OTU0825 | 3 | 14 | 11 | 3 | -0,9957811 | 0,35714286 | Peripherals |
| OTU0457 | 3 | 17 | 15 | 2 | -0,76289197 | 0,20761246 | Peripherals |
| OTU0099 | 2 | 9 | 5 | 4 | -0,47520464 | 0,56790123 | Peripherals |
| OTU0584 | 2 | 6 | 6 | 0 | -0,18544571 | 0 | Peripherals |
| OTU0907 | 6 | 9 | 9 | 0 | 1,32616776 | 0 | Peripherals |

| | | | | | | | |
|---|---|---|---|---|---|---|---|
| OTU0275 | 6 | 11 | 9 | 2 | 1,32616776 | 0,31404959 | Peripherals |
| OTU0655 | 3 | 6 | 5 | 1 | -1,34511479 | 0,27777778 | Peripherals |
| OTU0764 | 3 | 25 | 23 | 2 | -0,29711371 | 0,1504 | Peripherals |
| OTU0296 | 5 | 8 | 8 | 0 | -0,9085917 | 0 | Peripherals |
| OTU0390 | 5 | 21 | 21 | 0 | 0,81155763 | 0 | Peripherals |
| OTU0416 | 3 | 42 | 34 | 8 | 0,3433314 | 0,32312925 | Peripherals |
| OTU0311 | 2 | 5 | 5 | 0 | -0,47520464 | 0 | Peripherals |
| OTU0134 | 3 | 17 | 16 | 1 | -0,70466969 | 0,11072664 | Peripherals |
| OTU0359 | 2 | 11 | 9 | 2 | 0,68383107 | 0,31404959 | Peripherals |
| OTU0476 | 3 | 46 | 33 | 13 | 0,28510912 | 0,4168242 | Peripherals |
| OTU0433 | 3 | 6 | 5 | 1 | -1,34511479 | 0,27777778 | Peripherals |
| OTU0119 | 3 | 13 | 13 | 0 | -0,87933653 | 0 | Peripherals |
| OTU0769 | 5 | 21 | 21 | 0 | 0,81155763 | 0 | Peripherals |
| OTU0366 | 2 | 13 | 13 | 0 | 1,84286678 | 0 | Peripherals |
| OTU0633 | 1 | 29 | 17 | 12 | 1,59241269 | 0,51129608 | Peripherals |
| OTU0461 | 2 | 6 | 6 | 0 | -0,18544571 | 0 | Peripherals |
| OTU0720 | 1 | 8 | 4 | 4 | -0,70773897 | 0,59375 | Peripherals |
| OTU0897 | 2 | 6 | 6 | 0 | -0,18544571 | 0 | Peripherals |
| OTU0440 | 2 | 13 | 13 | 0 | 1,84286678 | 0 | Peripherals |
| OTU0199 | 3 | 19 | 18 | 1 | -0,58822512 | 0,09972299 | Peripherals |
| OTU0285 | 3 | 2 | 2 | 0 | -1,51978164 | 0 | Peripherals |
| OTU0812 | 6 | 3 | 1 | 2 | -1,18149491 | 0,44444444 | Peripherals |
| OTU0641 | 6 | 4 | 4 | 0 | -0,24112141 | 0 | Peripherals |
| OTU0056 | 6 | 12 | 11 | 1 | 1,95308343 | 0,15277778 | Peripherals |
| OTU0756 | 1 | 4 | 4 | 0 | -0,70773897 | 0 | Peripherals |
| OTU0499 | 1 | 12 | 12 | 0 | 0,70773897 | 0 | Peripherals |
| OTU0524 | 6 | 3 | 2 | 1 | -0,86803708 | 0,44444444 | Peripherals |
| OTU0154 | 1 | 4 | 4 | 0 | -0,70773897 | 0 | Peripherals |
| OTU0511 | 2 | 10 | 9 | 1 | 0,68383107 | 0,18 | Peripherals |
| OTU0277 | 2 | 17 | 10 | 7 | 0,97359 | 0,52595156 | Peripherals |
| OTU0713 | 2 | 7 | 7 | 0 | 0,10431321 | 0 | Peripherals |
| OTU0052 | 6 | 12 | 7 | 5 | 0,69925209 | 0,56944444 | Peripherals |
| OTU0671 | 1 | 6 | 4 | 2 | -0,70773897 | 0,5 | Peripherals |
| OTU0630 | 6 | 17 | 4 | 13 | -0,24112141 | 0,51903114 | Peripherals |
| OTU0156 | 6 | 8 | 7 | 1 | 0,69925209 | 0,21875 | Peripherals |
| OTU0245 | 6 | 7 | 4 | 3 | -0,24112141 | 0,57142857 | Peripherals |
| OTU0742 | 2 | 9 | 9 | 0 | 0,68383107 | 0 | Peripherals |
| OTU0931 | 2 | 7 | 4 | 3 | -0,76496357 | 0,48979592 | Peripherals |
| OTU0367 | 1 | 3 | 3 | 0 | -0,88467372 | 0 | Peripherals |
| OTU0936 | 6 | 7 | 6 | 1 | 0,38579426 | 0,24489796 | Peripherals |
| OTU0446 | 3 | 18 | 17 | 1 | -0,6464474 | 0,10493827 | Peripherals |
| OTU0760 | 3 | 34 | 32 | 2 | 0,22688683 | 0,11072664 | Peripherals |

| | | | | | | | |
|---|---|---|---|---|---|---|---|
| OTU0510 | 2 | 8 | 6 | 2 | -0,18544571 | 0,40625 | Peripherals |
| OTU0427 | 1 | 3 | 3 | 0 | -0,88467372 | 0 | Peripherals |
| OTU0279 | 2 | 2 | 1 | 1 | -1,63424035 | 0,5 | Peripherals |
| OTU0442 | 6 | 5 | 2 | 3 | -0,86803708 | 0,64 | Connector |
| OTU0334 | 4 | 2 | 1 | 1 | -0,70710678 | 0,5 | Peripherals |
| OTU0374 | 4 | 2 | 2 | 0 | 1,41421356 | 0 | Peripherals |
| OTU0514 | 3 | 4 | 3 | 1 | -1,46155936 | 0,375 | Peripherals |
| OTU0352 | 2 | 6 | 5 | 1 | -0,47520464 | 0,27777778 | Peripherals |
| OTU0670 | 2 | 9 | 4 | 5 | -0,76496357 | 0,59259259 | Peripherals |
| OTU0512 | 3 | 30 | 30 | 0 | 0,11044227 | 0 | Peripherals |
| OTU0102 | 3 | 1 | 1 | 0 | -1,57800392 | 0 | Peripherals |
| OTU0259 | 3 | 42 | 30 | 12 | 0,11044227 | 0,44104308 | Peripherals |
| OTU0891 | 6 | 6 | 5 | 1 | 0,07233642 | 0,27777778 | Peripherals |
| OTU0269 | 6 | 3 | 2 | 1 | -0,86803708 | 0,44444444 | Peripherals |
| OTU0740 | 1 | 13 | 9 | 4 | 0,17693474 | 0,4260355 | Peripherals |
| OTU0414 | 1 | 2 | 1 | 1 | -1,2385432 | 0,5 | Peripherals |
| OTU0743 | 5 | 2 | 2 | 0 | -1,70250677 | 0 | Peripherals |
| OTU0168 | 5 | 3 | 2 | 1 | -1,70250677 | 0,44444444 | Peripherals |
| OTU0265 | 3 | 13 | 11 | 2 | -0,9957811 | 0,26035503 | Peripherals |
| OTU0305 | 1 | 2 | 2 | 0 | -1,06160846 | 0 | Peripherals |
| OTU0801 | 6 | 6 | 1 | 5 | -1,18149491 | 0,5 | Peripherals |
| OTU0585 | 3 | 3 | 3 | 0 | -1,46155936 | 0 | Peripherals |
| OTU0084 | 3 | 4 | 4 | 0 | -1,40333708 | 0 | Peripherals |
| OTU0299 | 2 | 4 | 4 | 0 | -0,76496357 | 0 | Peripherals |
| OTU0298 | 2 | 3 | 3 | 0 | -1,0547225 | 0 | Peripherals |
| OTU0654 | 2 | 8 | 5 | 3 | -0,47520464 | 0,46875 | Peripherals |
| OTU0187 | 3 | 3 | 3 | 0 | -1,46155936 | 0 | Peripherals |
| OTU0137 | 1 | 20 | 15 | 5 | 1,2385432 | 0,395 | Peripherals |
| OTU0129 | 2 | 9 | 8 | 1 | 0,39407214 | 0,19753086 | Peripherals |
| OTU0123 | 1 | 1 | 1 | 0 | -1,2385432 | 0 | Peripherals |
| OTU0092 | 1 | 5 | 3 | 2 | -0,88467372 | 0,48 | Peripherals |
| OTU0335 | 6 | 2 | 1 | 1 | -1,18149491 | 0,5 | Peripherals |
| OTU0618 | 2 | 1 | 1 | 0 | -1,63424035 | 0 | Peripherals |
| OTU0197 | 3 | 9 | 9 | 0 | -1,11222566 | 0 | Peripherals |
| OTU0537 | 6 | 2 | 2 | 0 | -0,86803708 | 0 | Peripherals |
| OTU0902 | 6 | 5 | 5 | 0 | 0,07233642 | 0 | Peripherals |
| OTU0738 | 1 | 10 | 10 | 0 | 0,35386949 | 0 | Peripherals |
| OTU0900 | 3 | 1 | 1 | 0 | -1,57800392 | 0 | Peripherals |
| OTU0635 | 5 | 3 | 2 | 1 | -1,70250677 | 0,44444444 | Peripherals |
| OTU0138 | 1 | 2 | 2 | 0 | -1,06160846 | 0 | Peripherals |
| OTU0053 | 2 | 3 | 1 | 2 | -1,63424035 | 0,44444444 | Peripherals |
| OTU0806 | 2 | 1 | 1 | 0 | -1,63424035 | 0 | Peripherals |

| | | | | | | | |
|---|---|---|---|---|---|---|---|
| OTU0852 | 3 | 3 | 3 | 0 | -1,46155936 | 0 | Peripherals |
| OTU0799 | 2 | 3 | 3 | 0 | -1,0547225 | 0 | Peripherals |
| OTU0329 | 5 | 2 | 2 | 0 | -1,70250677 | 0 | Peripherals |
| OTU0628 | 4 | 1 | 1 | 0 | -0,70710678 | 0 | Peripherals |
| OTU0842 | 3 | 1 | 1 | 0 | -1,57800392 | 0 | Peripherals |
| OTU0358 | 3 | 3 | 3 | 0 | -1,46155936 | 0 | Peripherals |
| OTU0300 | 1 | 2 | 2 | 0 | -1,06160846 | 0 | Peripherals |
| OTU0364 | 2 | 2 | 2 | 0 | -1,34448143 | 0 | Peripherals |
| OTU0850 | 1 | 1 | 1 | 0 | -1,2385432 | 0 | Peripherals |

**B** Prebiotic bath treatment:

| Node | Module | Edges | | | Zi (within) | pi(outside) | Role |
|---|---|---|---|---|---|---|---|
| | | All | Inside Module | Outside Module | | | |
| OTU0519 | 1 | 29 | 26 | 3 | 1,20356361 | 0,18549346 | Peripherals |
| OTU0093 | 8 | 15 | 9 | 6 | -0,22976102 | 0,48 | Peripherals |
| OTU0187 | 1 | 30 | 29 | 1 | 1,53056957 | 0,06444444 | Peripherals |
| OTU0193 | 8 | 15 | 6 | 9 | -0,70137575 | 0,48 | Peripherals |
| OTU0583 | 8 | 19 | 18 | 1 | 1,18508316 | 0,09972299 | Peripherals |
| OTU0749 | 8 | 17 | 16 | 1 | 0,87067334 | 0,11072664 | Peripherals |
| OTU0091 | 1 | 26 | 25 | 1 | 1,09456162 | 0,0739645 | Peripherals |
| OTU0684 | 1 | 26 | 25 | 1 | 1,09456162 | 0,0739645 | Peripherals |
| OTU0805 | 1 | 15 | 14 | 1 | -0,10446024 | 0,12444444 | Peripherals |
| OTU0084 | 8 | 13 | 13 | 0 | 0,39905861 | 0 | Peripherals |
| OTU0437 | 8 | 22 | 21 | 1 | 1,65669788 | 0,08677686 | Peripherals |
| OTU0553 | 7 | 6 | 3 | 3 | -0,26388991 | 0,66666667 | Connector |
| OTU0176 | 11 | 14 | 12 | 2 | -0,32585274 | 0,25510204 | Peripherals |
| OTU0062 | 8 | 17 | 16 | 1 | 0,87067334 | 0,11072664 | Peripherals |
| OTU0331 | 11 | 22 | 21 | 1 | 0,77390026 | 0,08677686 | Peripherals |
| OTU0342 | 11 | 21 | 21 | 0 | 0,77390026 | 0 | Peripherals |
| OTU0654 | 1 | 11 | 8 | 3 | -0,75847216 | 0,39669421 | Peripherals |
| OTU0597 | 3 | 13 | 11 | 2 | 1,93313844 | 0,26035503 | Peripherals |
| OTU0007 | 1 | 28 | 24 | 4 | 0,98555963 | 0,25255102 | Peripherals |
| OTU0586 | 1 | 25 | 13 | 12 | -0,21346222 | 0,5344 | Peripherals |
| OTU0642 | 1 | 24 | 24 | 0 | 0,98555963 | 0 | Peripherals |
| OTU0748 | 1 | 23 | 22 | 1 | 0,76755566 | 0,0831758 | Peripherals |
| OTU0137 | 1 | 23 | 20 | 3 | 0,54955169 | 0,23818526 | Peripherals |
| OTU0351 | 1 | 23 | 22 | 1 | 0,76755566 | 0,0831758 | Peripherals |
| OTU0237 | 11 | 23 | 23 | 0 | 1,01828981 | 0 | Peripherals |
| OTU0706 | 11 | 5 | 4 | 1 | -1,30341096 | 0,32 | Peripherals |
| OTU0520 | 4 | 15 | 8 | 7 | -0,09950372 | 0,59555556 | Peripherals |
| OTU0392 | 4 | 23 | 13 | 10 | 0,89553347 | 0,57844991 | Peripherals |

| | | | | | | | |
|---|---|---|---|---|---|---|---|
| OTU0787 | 4 | 14 | 9 | 5 | 0,09950372 | 0,45918367 | Peripherals |
| OTU0639 | 4 | 22 | 15 | 7 | 1,29354835 | 0,49173554 | Peripherals |
| OTU0817 | 3 | 15 | 14 | 1 | 2,89970766 | 0,12444444 | Module Hub |
| OTU0933 | 3 | 11 | 8 | 3 | 0,96656922 | 0,42975207 | Peripherals |
| OTU0373 | 1 | 37 | 29 | 8 | 1,53056957 | 0,35646457 | Peripherals |
| OTU0836 | 1 | 26 | 14 | 12 | -0,10446024 | 0,49704142 | Peripherals |
| OTU0241 | 11 | 2 | 2 | 0 | -1,54780052 | 0 | Peripherals |
| OTU0053 | 11 | 10 | 9 | 1 | -0,69243707 | 0,18 | Peripherals |
| OTU0667 | 8 | 14 | 13 | 1 | 0,39905861 | 0,13265306 | Peripherals |
| OTU0930 | 8 | 5 | 5 | 0 | -0,85858065 | 0 | Peripherals |
| OTU0043 | 11 | 21 | 21 | 0 | 0,77390026 | 0 | Peripherals |
| OTU0111 | 11 | 26 | 26 | 0 | 1,38487415 | 0 | Peripherals |
| OTU0338 | 11 | 25 | 23 | 2 | 1,01828981 | 0,1472 | Peripherals |
| OTU0856 | 1 | 29 | 28 | 1 | 1,42156758 | 0,0665874 | Peripherals |
| OTU0168 | 11 | 25 | 25 | 0 | 1,26267937 | 0 | Peripherals |
| OTU0624 | 11 | 27 | 27 | 0 | 1,50706892 | 0 | Peripherals |
| OTU0002 | 3 | 11 | 11 | 0 | 1,93313844 | 0 | Peripherals |
| OTU0528 | 3 | 11 | 10 | 1 | 1,6109487 | 0,16528926 | Peripherals |
| OTU0892 | 11 | 8 | 8 | 0 | -0,81463185 | 0 | Peripherals |
| OTU0889 | 3 | 9 | 8 | 1 | 0,96656922 | 0,19753086 | Peripherals |
| OTU0276 | 11 | 25 | 25 | 0 | 1,26267937 | 0 | Peripherals |
| OTU0756 | 11 | 17 | 17 | 0 | 0,28512115 | 0 | Peripherals |
| OTU0148 | 5 | 8 | 8 | 0 | 0,5 | 0 | Peripherals |
| OTU0489 | 5 | 7 | 7 | 0 | 0 | 0 | Peripherals |
| OTU0830 | 1 | 24 | 20 | 4 | 0,54955169 | 0,28819444 | Peripherals |
| OTU0724 | 1 | 27 | 16 | 11 | 0,11354374 | 0,48285322 | Peripherals |
| OTU0258 | 1 | 11 | 10 | 1 | -0,54046819 | 0,16528926 | Peripherals |
| OTU0909 | 3 | 3 | 1 | 2 | -1,28875896 | 0,44444444 | Peripherals |
| OTU0049 | 10 | 5 | 4 | 1 | 1,5 | 0,32 | Peripherals |
| OTU0712 | 10 | 5 | 3 | 2 | 0,5 | 0,56 | Peripherals |
| OTU0123 | 5 | 5 | 5 | 0 | -1 | 0 | Peripherals |
| OTU0387 | 4 | 12 | 8 | 4 | -0,09950372 | 0,44444444 | Peripherals |
| OTU0476 | 1 | 21 | 19 | 2 | 0,4405497 | 0,1723356 | Peripherals |
| OTU0669 | 1 | 26 | 26 | 0 | 1,20356361 | 0 | Peripherals |
| OTU0456 | 8 | 7 | 7 | 0 | -0,54417084 | 0 | Peripherals |
| OTU0082 | 8 | 17 | 17 | 0 | 1,02787825 | 0 | Peripherals |
| OTU0411 | 1 | 16 | 10 | 6 | -0,54046819 | 0,46875 | Peripherals |
| OTU0543 | 1 | 35 | 28 | 7 | 1,42156758 | 0,34612245 | Peripherals |
| OTU0272 | 4 | 29 | 19 | 10 | 2,0895781 | 0,48989298 | Peripherals |
| OTU0405 | 4 | 12 | 7 | 5 | -0,29851116 | 0,48611111 | Peripherals |
| OTU0074 | 1 | 29 | 27 | 2 | 1,3125656 | 0,13079667 | Peripherals |
| OTU0556 | 1 | 25 | 25 | 0 | 1,09456162 | 0 | Peripherals |

| | | | | | | | |
|---|---|---|---|---|---|---|---|
| OTU0795 | 1 | 23 | 23 | 0 | 0,87655765 | 0 | Peripherals |
| OTU0240 | 11 | 28 | 28 | 0 | 1,6292637 | 0 | Peripherals |
| OTU0725 | 8 | 18 | 18 | 0 | 1,18508316 | 0 | Peripherals |
| OTU0329 | 11 | 21 | 21 | 0 | 0,77390026 | 0 | Peripherals |
| OTU0461 | 11 | 17 | 17 | 0 | 0,28512115 | 0 | Peripherals |
| OTU0119 | 5 | 8 | 8 | 0 | 0,5 | 0 | Peripherals |
| OTU0127 | 1 | 31 | 31 | 0 | 1,74857354 | 0 | Peripherals |
| OTU0189 | 1 | 17 | 16 | 1 | 0,11354374 | 0,11072664 | Peripherals |
| OTU0450 | 4 | 19 | 13 | 6 | 0,89553347 | 0,47645429 | Peripherals |
| OTU0319 | 4 | 14 | 9 | 5 | 0,09950372 | 0,45918367 | Peripherals |
| OTU0577 | 4 | 20 | 13 | 7 | 0,89553347 | 0,485 | Peripherals |
| OTU0430 | 1 | 14 | 13 | 1 | -0,21346222 | 0,13265306 | Peripherals |
| OTU0809 | 3 | 6 | 3 | 3 | -0,64437948 | 0,5 | Peripherals |
| OTU0181 | 3 | 11 | 4 | 7 | -0,32218974 | 0,7107438 | Connector |
| OTU0561 | 1 | 19 | 19 | 0 | 0,4405497 | 0 | Peripherals |
| OTU0046 | 11 | 21 | 21 | 0 | 0,77390026 | 0 | Peripherals |
| OTU0081 | 4 | 25 | 13 | 12 | 0,89553347 | 0,592 | Peripherals |
| OTU0183 | 3 | 8 | 8 | 0 | 0,96656922 | 0 | Peripherals |
| OTU0182 | 3 | 9 | 9 | 0 | 1,28875896 | 0 | Peripherals |
| OTU0564 | 1 | 32 | 22 | 10 | 0,76755566 | 0,46289063 | Peripherals |
| OTU0733 | 3 | 4 | 2 | 2 | -0,96656922 | 0,5 | Peripherals |
| OTU0633 | 1 | 26 | 21 | 5 | 0,65855367 | 0,31065089 | Peripherals |
| OTU0201 | 1 | 28 | 21 | 7 | 0,65855367 | 0,4005102 | Peripherals |
| OTU0234 | 11 | 12 | 12 | 0 | -0,32585274 | 0 | Peripherals |
| OTU0612 | 1 | 16 | 15 | 1 | 0,00454175 | 0,1171875 | Peripherals |
| OTU0427 | 1 | 20 | 20 | 0 | 0,54955169 | 0 | Peripherals |
| OTU0719 | 11 | 9 | 7 | 2 | -0,93682663 | 0,34567901 | Peripherals |
| OTU0705 | 11 | 5 | 4 | 1 | -1,30341096 | 0,32 | Peripherals |
| OTU0714 | 1 | 4 | 3 | 1 | -1,3034821 | 0,375 | Peripherals |
| OTU0885 | 1 | 29 | 21 | 8 | 0,65855367 | 0,41617122 | Peripherals |
| OTU0713 | 11 | 3 | 3 | 0 | -1,42560574 | 0 | Peripherals |
| OTU0896 | 8 | 16 | 16 | 0 | 0,87067334 | 0 | Peripherals |
| OTU0452 | 8 | 15 | 15 | 0 | 0,71346843 | 0 | Peripherals |
| OTU0600 | 4 | 17 | 11 | 6 | 0,4975186 | 0,49134948 | Peripherals |
| OTU0655 | 1 | 11 | 8 | 3 | -0,75847216 | 0,39669421 | Peripherals |
| OTU0190 | 1 | 9 | 9 | 0 | -0,64947017 | 0 | Peripherals |
| OTU0448 | 11 | 21 | 21 | 0 | 0,77390026 | 0 | Peripherals |
| OTU0730 | 1 | 17 | 14 | 3 | -0,10446024 | 0,29065744 | Peripherals |
| OTU0352 | 1 | 30 | 29 | 1 | 1,53056957 | 0,06444444 | Peripherals |
| OTU0499 | 1 | 21 | 21 | 0 | 0,65855367 | 0 | Peripherals |
| OTU0274 | 1 | 9 | 9 | 0 | -0,64947017 | 0 | Peripherals |
| OTU0107 | 5 | 8 | 8 | 0 | 0,5 | 0 | Peripherals |

| | | | | | | | |
|---|---|---|---|---|---|---|---|
| OTU0277 | 8 | 11 | 11 | 0 | 0,0846488 | 0 | Peripherals |
| OTU0440 | 8 | 20 | 20 | 0 | 1,49949297 | 0 | Peripherals |
| OTU0086 | 1 | 12 | 12 | 0 | -0,32246421 | 0 | Peripherals |
| OTU0665 | 8 | 5 | 5 | 0 | -0,85858065 | 0 | Peripherals |
| OTU0340 | 8 | 5 | 4 | 1 | -1,01578556 | 0,32 | Peripherals |
| OTU0174 | 3 | 4 | 2 | 2 | -0,96656922 | 0,625 | Connector |
| OTU0433 | 8 | 15 | 14 | 1 | 0,55626352 | 0,12444444 | Peripherals |
| OTU0702 | 1 | 6 | 5 | 1 | -1,08547812 | 0,27777778 | Peripherals |
| OTU0891 | 11 | 22 | 22 | 0 | 0,89609504 | 0 | Peripherals |
| OTU0897 | 8 | 9 | 9 | 0 | -0,22976102 | 0 | Peripherals |
| OTU0153 | 8 | 21 | 21 | 0 | 1,65669788 | 0 | Peripherals |
| OTU0807 | 1 | 19 | 19 | 0 | 0,4405497 | 0 | Peripherals |
| OTU0902 | 3 | 11 | 11 | 0 | 1,93313844 | 0 | Peripherals |
| OTU0742 | 7 | 2 | 2 | 0 | -0,68611376 | 0 | Peripherals |
| OTU0191 | 7 | 8 | 8 | 0 | 1,84722935 | 0 | Peripherals |
| OTU0585 | 3 | 4 | 4 | 0 | -0,32218974 | 0 | Peripherals |
| OTU0120 | 3 | 7 | 7 | 0 | 0,64437948 | 0 | Peripherals |
| OTU0383 | 1 | 9 | 9 | 0 | -0,64947017 | 0 | Peripherals |
| OTU0374 | 11 | 15 | 15 | 0 | 0,04073159 | 0 | Peripherals |
| OTU0893 | 8 | 19 | 19 | 0 | 1,34228806 | 0 | Peripherals |
| OTU0574 | 4 | 13 | 10 | 3 | 0,29851116 | 0,35502959 | Peripherals |
| OTU0269 | 1 | 21 | 21 | 0 | 0,65855367 | 0 | Peripherals |
| OTU0165 | 3 | 6 | 6 | 0 | 0,32218974 | 0 | Peripherals |
| OTU0593 | 3 | 5 | 5 | 0 | 0 | 0 | Peripherals |
| OTU0592 | 3 | 4 | 4 | 0 | -0,32218974 | 0 | Peripherals |
| OTU0265 | 1 | 31 | 30 | 1 | 1,63957156 | 0,06243496 | Peripherals |
| OTU0762 | 11 | 9 | 9 | 0 | -0,69243707 | 0 | Peripherals |
| OTU0328 | 11 | 10 | 8 | 2 | -0,81463185 | 0,34 | Peripherals |
| OTU0014 | 1 | 29 | 28 | 1 | 1,42156758 | 0,0665874 | Peripherals |
| OTU0256 | 1 | 13 | 13 | 0 | -0,21346222 | 0 | Peripherals |
| OTU0524 | 11 | 10 | 6 | 4 | -1,05902141 | 0,56 | Peripherals |
| OTU0420 | 8 | 11 | 10 | 1 | -0,07255611 | 0,16528926 | Peripherals |
| OTU0523 | 10 | 5 | 3 | 2 | 0,5 | 0,56 | Peripherals |
| OTU0412 | 8 | 13 | 13 | 0 | 0,39905861 | 0 | Peripherals |
| OTU0147 | 7 | 6 | 5 | 1 | 0,5805578 | 0,27777778 | Peripherals |
| OTU0740 | 7 | 9 | 9 | 0 | 2,2694532 | 0 | Peripherals |
| OTU0542 | 1 | 32 | 26 | 6 | 1,20356361 | 0,3203125 | Peripherals |
| OTU0381 | 1 | 26 | 25 | 1 | 1,09456162 | 0,0739645 | Peripherals |
| OTU0217 | 1 | 21 | 21 | 0 | 0,65855367 | 0 | Peripherals |
| OTU0445 | 11 | 20 | 20 | 0 | 0,65170548 | 0 | Peripherals |
| OTU0275 | 11 | 27 | 27 | 0 | 1,50706892 | 0 | Peripherals |
| OTU0575 | 11 | 9 | 8 | 1 | -0,81463185 | 0,19753086 | Peripherals |

| | | | | | | | |
|---|---|---|---|---|---|---|---|
| OTU0400 | 1 | 4 | 4 | 0 | -1,19448011 | 0 | Peripherals |
| OTU0159 | 1 | 11 | 10 | 1 | -0,54046819 | 0,16528926 | Peripherals |
| OTU0026 | 8 | 17 | 17 | 0 | 1,02787825 | 0 | Peripherals |
| OTU0099 | 8 | 16 | 16 | 0 | 0,87067334 | 0 | Peripherals |
| OTU0262 | 8 | 21 | 6 | 15 | -0,70137575 | 0,65306122 | Connector |
| OTU0755 | 3 | 5 | 5 | 0 | 0 | 0 | Peripherals |
| OTU0880 | 3 | 8 | 5 | 3 | 0 | 0,53125 | Peripherals |
| OTU0423 | 1 | 14 | 12 | 2 | -0,32246421 | 0,25510204 | Peripherals |
| OTU0051 | 4 | 19 | 16 | 3 | 1,49255579 | 0,28254848 | Peripherals |
| OTU0602 | 1 | 9 | 7 | 2 | -0,86747415 | 0,34567901 | Peripherals |
| OTU0663 | 1 | 33 | 33 | 0 | 1,96657752 | 0 | Peripherals |
| OTU0300 | 1 | 9 | 7 | 2 | -0,86747415 | 0,34567901 | Peripherals |
| OTU0482 | 8 | 13 | 13 | 0 | 0,39905861 | 0 | Peripherals |
| OTU0629 | 8 | 23 | 23 | 0 | 1,9711077 | 0 | Peripherals |
| OTU0465 | 7 | 4 | 2 | 2 | -0,68611376 | 0,5 | Peripherals |
| OTU0761 | 11 | 7 | 6 | 1 | -1,05902141 | 0,24489796 | Peripherals |
| OTU0626 | 8 | 12 | 12 | 0 | 0,24185371 | 0 | Peripherals |
| OTU0815 | 1 | 11 | 11 | 0 | -0,4314662 | 0 | Peripherals |
| OTU0075 | 3 | 12 | 5 | 7 | 0 | 0,625 | Connector |
| OTU0294 | 3 | 5 | 3 | 2 | -0,64437948 | 0,56 | Peripherals |
| OTU0371 | 8 | 12 | 8 | 4 | -0,38696593 | 0,51388889 | Peripherals |
| OTU0492 | 8 | 14 | 14 | 0 | 0,55626352 | 0 | Peripherals |
| OTU0764 | 4 | 13 | 10 | 3 | 0,29851116 | 0,37869822 | Peripherals |
| OTU0198 | 8 | 13 | 11 | 2 | 0,0846488 | 0,26035503 | Peripherals |
| OTU0745 | 1 | 20 | 18 | 2 | 0,33154771 | 0,18 | Peripherals |
| OTU0335 | 11 | 23 | 22 | 1 | 0,89609504 | 0,0831758 | Peripherals |
| OTU0644 | 1 | 13 | 12 | 1 | -0,32246421 | 0,14201183 | Peripherals |
| OTU0019 | 1 | 7 | 4 | 3 | -1,19448011 | 0,57142857 | Peripherals |
| OTU0407 | 3 | 3 | 3 | 0 | -0,64437948 | 0 | Peripherals |
| OTU0375 | 1 | 14 | 13 | 1 | -0,21346222 | 0,13265306 | Peripherals |
| OTU0673 | 4 | 16 | 9 | 7 | 0,09950372 | 0,5703125 | Peripherals |
| OTU0140 | 8 | 5 | 5 | 0 | -0,85858065 | 0 | Peripherals |
| OTU0878 | 1 | 32 | 32 | 0 | 1,85757553 | 0 | Peripherals |
| OTU0717 | 1 | 14 | 13 | 1 | -0,21346222 | 0,13265306 | Peripherals |
| OTU0516 | 1 | 14 | 11 | 3 | -0,4314662 | 0,35714286 | Peripherals |
| OTU0154 | 7 | 4 | 4 | 0 | 0,15833394 | 0 | Peripherals |
| OTU0540 | 3 | 10 | 9 | 1 | 1,28875896 | 0,18 | Peripherals |
| OTU0333 | 11 | 18 | 17 | 1 | 0,28512115 | 0,10493827 | Peripherals |
| OTU0630 | 11 | 18 | 18 | 0 | 0,40731593 | 0 | Peripherals |
| OTU0774 | 8 | 5 | 5 | 0 | -0,85858065 | 0 | Peripherals |
| OTU0416 | 1 | 8 | 8 | 0 | -0,75847216 | 0 | Peripherals |
| OTU0505 | 1 | 21 | 21 | 0 | 0,65855367 | 0 | Peripherals |

| | | | | | | | |
|---|---|---|---|---|---|---|---|
| OTU0245 | 3 | 4 | 3 | 1 | -0,64437948 | 0,375 | Peripherals |
| OTU0769 | 3 | 3 | 1 | 2 | -1,28875896 | 0,44444444 | Peripherals |
| OTU0907 | 7 | 5 | 5 | 0 | 0,5805578 | 0 | Peripherals |
| OTU0263 | 7 | 4 | 4 | 0 | 0,15833394 | 0 | Peripherals |
| OTU0850 | 4 | 2 | 1 | 1 | -1,49255579 | 0,5 | Peripherals |
| OTU0366 | 3 | 10 | 3 | 7 | -0,64437948 | 0,8 | Connector |
| OTU0088 | 4 | 2 | 1 | 1 | -1,49255579 | 0,5 | Peripherals |
| OTU0480 | 8 | 8 | 8 | 0 | -0,38696593 | 0 | Peripherals |
| OTU0285 | 11 | 16 | 15 | 1 | 0,04073159 | 0,1171875 | Peripherals |
| OTU0635 | 11 | 4 | 2 | 2 | -1,54780052 | 0,625 | Connector |
| OTU0697 | 8 | 3 | 2 | 1 | -1,33019538 | 0,44444444 | Peripherals |
| OTU0582 | 11 | 25 | 25 | 0 | 1,26267937 | 0 | Peripherals |
| OTU0029 | 1 | 12 | 11 | 1 | -0,4314662 | 0,15277778 | Peripherals |
| OTU0446 | 8 | 21 | 21 | 0 | 1,65669788 | 0 | Peripherals |
| OTU0283 | 8 | 18 | 18 | 0 | 1,18508316 | 0 | Peripherals |
| OTU0298 | 8 | 8 | 8 | 0 | -0,38696593 | 0 | Peripherals |
| OTU0908 | 3 | 11 | 2 | 9 | -0,96656922 | 0,52892562 | Peripherals |
| OTU0868 | 1 | 5 | 3 | 2 | -1,3034821 | 0,56 | Peripherals |
| OTU0859 | 4 | 7 | 4 | 3 | -0,89553347 | 0,57142857 | Peripherals |
| OTU0056 | 8 | 4 | 3 | 1 | -1,17299047 | 0,375 | Peripherals |
| OTU0304 | 3 | 11 | 11 | 0 | 1,93313844 | 0 | Peripherals |
| OTU0739 | 3 | 9 | 9 | 0 | 1,28875896 | 0 | Peripherals |
| OTU0736 | 1 | 24 | 22 | 2 | 0,76755566 | 0,15277778 | Peripherals |
| OTU0054 | 11 | 4 | 4 | 0 | -1,30341096 | 0 | Peripherals |
| OTU0323 | 11 | 22 | 22 | 0 | 0,89609504 | 0 | Peripherals |
| OTU0128 | 8 | 10 | 10 | 0 | -0,07255611 | 0 | Peripherals |
| OTU0141 | 8 | 5 | 5 | 0 | -0,85858065 | 0 | Peripherals |
| OTU0525 | 1 | 9 | 7 | 2 | -0,86747415 | 0,34567901 | Peripherals |
| OTU0435 | 11 | 7 | 6 | 1 | -1,05902141 | 0,24489796 | Peripherals |
| OTU0118 | 1 | 5 | 4 | 1 | -1,19448011 | 0,32 | Peripherals |
| OTU0122 | 3 | 5 | 5 | 0 | 0 | 0 | Peripherals |
| OTU0169 | 3 | 9 | 9 | 0 | 1,28875896 | 0 | Peripherals |
| OTU0925 | 3 | 5 | 5 | 0 | 0 | 0 | Peripherals |
| OTU0462 | 4 | 4 | 3 | 1 | -1,09454091 | 0,375 | Peripherals |
| OTU0486 | 1 | 12 | 11 | 1 | -0,4314662 | 0,15277778 | Peripherals |
| OTU0689 | 8 | 22 | 22 | 0 | 1,81390279 | 0 | Peripherals |
| OTU0501 | 1 | 22 | 20 | 2 | 0,54955169 | 0,16528926 | Peripherals |
| OTU0513 | 3 | 7 | 6 | 1 | 0,32218974 | 0,24489796 | Peripherals |
| OTU0124 | 8 | 10 | 7 | 3 | -0,54417084 | 0,46 | Peripherals |
| OTU0334 | 11 | 11 | 6 | 5 | -1,05902141 | 0,49586777 | Peripherals |
| OTU0491 | 11 | 5 | 3 | 2 | -1,42560574 | 0,48 | Peripherals |
| OTU0754 | 1 | 6 | 5 | 1 | -1,08547812 | 0,27777778 | Peripherals |

| | | | | | | | |
|---|---|---|---|---|---|---|---|
| OTU0125 | 3 | 7 | 7 | 0 | 0,64437948 | 0 | Peripherals |
| OTU0197 | 3 | 5 | 5 | 0 | 0 | 0 | Peripherals |
| OTU0442 | 11 | 20 | 20 | 0 | 0,65170548 | 0 | Peripherals |
| OTU0305 | 8 | 24 | 24 | 0 | 2,12831261 | 0 | Peripherals |
| OTU0050 | 1 | 8 | 8 | 0 | -0,75847216 | 0 | Peripherals |
| OTU0359 | 1 | 3 | 3 | 0 | -1,3034821 | 0 | Peripherals |
| OTU0447 | 8 | 19 | 19 | 0 | 1,34228806 | 0 | Peripherals |
| OTU0692 | 5 | 7 | 7 | 0 | 0 | 0 | Peripherals |
| OTU0203 | 3 | 4 | 4 | 0 | -0,32218974 | 0 | Peripherals |
| OTU0618 | 3 | 1 | 1 | 0 | -1,28875896 | 0 | Peripherals |
| OTU0912 | 8 | 2 | 2 | 0 | -1,33019538 | 0 | Peripherals |
| OTU0399 | 3 | 7 | 5 | 2 | 0 | 0,40816327 | Peripherals |
| OTU0458 | 11 | 17 | 13 | 4 | -0,20365796 | 0,38062284 | Peripherals |
| OTU0722 | 3 | 6 | 5 | 1 | 0 | 0,27777778 | Peripherals |
| OTU0175 | 3 | 7 | 5 | 2 | 0 | 0,40816327 | Peripherals |
| OTU0641 | 5 | 9 | 9 | 0 | 1 | 0 | Peripherals |
| OTU0231 | 1 | 9 | 8 | 1 | -0,75847216 | 0,19753086 | Peripherals |
| OTU0220 | 3 | 7 | 4 | 3 | -0,32218974 | 0,48979592 | Peripherals |
| OTU0259 | 1 | 37 | 35 | 2 | 2,18458149 | 0,10372535 | Peripherals |
| OTU0671 | 8 | 9 | 8 | 1 | -0,38696593 | 0,19753086 | Peripherals |
| OTU0016 | 3 | 5 | 3 | 2 | -0,64437948 | 0,56 | Peripherals |
| OTU0410 | 8 | 13 | 10 | 3 | -0,07255611 | 0,39053254 | Peripherals |
| OTU0932 | 7 | 6 | 6 | 0 | 1,00278165 | 0 | Peripherals |
| OTU0377 | 4 | 7 | 6 | 1 | -0,4975186 | 0,24489796 | Peripherals |
| OTU0512 | 1 | 11 | 10 | 1 | -0,54046819 | 0,16528926 | Peripherals |
| OTU0073 | 3 | 3 | 2 | 1 | -0,96656922 | 0,44444444 | Peripherals |
| OTU0292 | 3 | 2 | 2 | 0 | -0,96656922 | 0 | Peripherals |
| OTU0603 | 1 | 7 | 7 | 0 | -0,86747415 | 0 | Peripherals |
| OTU0077 | 4 | 8 | 4 | 4 | -0,89553347 | 0,59375 | Peripherals |
| OTU0199 | 3 | 6 | 6 | 0 | 0,32218974 | 0 | Peripherals |
| OTU0184 | 3 | 1 | 1 | 0 | -1,28875896 | 0 | Peripherals |
| OTU0432 | 8 | 1 | 1 | 0 | -1,48740029 | 0 | Peripherals |
| OTU0330 | 11 | 6 | 6 | 0 | -1,05902141 | 0 | Peripherals |
| OTU0743 | 5 | 9 | 9 | 0 | 1 | 0 | Peripherals |
| OTU0390 | 8 | 9 | 9 | 0 | -0,22976102 | 0 | Peripherals |
| OTU0584 | 1 | 2 | 2 | 0 | -1,41248408 | 0 | Peripherals |
| OTU0428 | 3 | 6 | 4 | 2 | -0,32218974 | 0,44444444 | Peripherals |
| OTU0511 | 8 | 8 | 6 | 2 | -0,70137575 | 0,40625 | Peripherals |
| OTU0509 | 8 | 7 | 5 | 2 | -0,85858065 | 0,44897959 | Peripherals |
| OTU0895 | 10 | 4 | 1 | 3 | -1,5 | 0,625 | Connector |
| OTU0589 | 1 | 7 | 4 | 3 | -1,19448011 | 0,57142857 | Peripherals |
| OTU0573 | 10 | 5 | 3 | 2 | 0,5 | 0,56 | Peripherals |

| | | | | | | | |
|---|---|---|---|---|---|---|---|
| OTU0065 | 3 | 8 | 8 | 0 | 0,96656922 | 0 | Peripherals |
| OTU0303 | 3 | 5 | 5 | 0 | 0 | 0 | Peripherals |
| OTU0365 | 3 | 3 | 1 | 2 | -1,28875896 | 0,66666667 | Connector |
| OTU0317 | 8 | 9 | 9 | 0 | -0,22976102 | 0 | Peripherals |
| OTU0903 | 8 | 7 | 7 | 0 | -0,54417084 | 0 | Peripherals |
| OTU0108 | 8 | 3 | 2 | 1 | -1,33019538 | 0,44444444 | Peripherals |
| OTU0899 | 1 | 2 | 2 | 0 | -1,41248408 | 0 | Peripherals |
| OTU0142 | 5 | 7 | 7 | 0 | 0 | 0 | Peripherals |
| OTU0852 | 8 | 9 | 9 | 0 | -0,22976102 | 0 | Peripherals |
| OTU0738 | 8 | 6 | 3 | 3 | -1,17299047 | 0,5 | Peripherals |
| OTU0605 | 3 | 5 | 4 | 1 | -0,32218974 | 0,32 | Peripherals |
| OTU0570 | 1 | 7 | 4 | 3 | -1,19448011 | 0,48979592 | Peripherals |
| OTU0126 | 8 | 2 | 2 | 0 | -1,33019538 | 0 | Peripherals |
| OTU0057 | 3 | 7 | 4 | 3 | -0,32218974 | 0,57142857 | Peripherals |
| OTU0178 | 3 | 4 | 4 | 0 | -0,32218974 | 0 | Peripherals |
| OTU0572 | 7 | 2 | 1 | 1 | -1,10833761 | 0,5 | Peripherals |
| OTU0535 | 8 | 3 | 3 | 0 | -1,17299047 | 0 | Peripherals |
| OTU0069 | 1 | 5 | 3 | 2 | -1,3034821 | 0,56 | Peripherals |
| OTU0757 | 1 | 7 | 7 | 0 | -0,86747415 | 0 | Peripherals |
| OTU0737 | 3 | 2 | 2 | 0 | -0,96656922 | 0 | Peripherals |
| OTU0822 | 1 | 3 | 3 | 0 | -1,3034821 | 0 | Peripherals |
| OTU0018 | 7 | 2 | 2 | 0 | -0,68611376 | 0 | Peripherals |
| OTU0783 | 8 | 3 | 3 | 0 | -1,17299047 | 0 | Peripherals |
| OTU0192 | 8 | 6 | 3 | 3 | -1,17299047 | 0,61111111 | Peripherals |
| OTU0494 | 11 | 6 | 6 | 0 | -1,05902141 | 0 | Peripherals |
| OTU0825 | 3 | 1 | 1 | 0 | -1,28875896 | 0 | Peripherals |
| OTU0866 | 3 | 2 | 2 | 0 | -0,96656922 | 0 | Peripherals |
| OTU0484 | 1 | 5 | 5 | 0 | -1,08547812 | 0 | Peripherals |
| OTU0242 | 7 | 3 | 3 | 0 | -0,26388991 | 0 | Peripherals |
| OTU0594 | 4 | 1 | 1 | 0 | -1,49255579 | 0 | Peripherals |
| OTU0236 | 10 | 2 | 2 | 0 | -0,5 | 0 | Peripherals |
| OTU0439 | 8 | 4 | 2 | 2 | -1,33019538 | 0,625 | Connector |
| OTU0367 | 7 | 3 | 2 | 1 | -0,68611376 | 0,44444444 | Peripherals |
| OTU0443 | 10 | 4 | 3 | 1 | 0,5 | 0,375 | Peripherals |
| OTU0042 | 10 | 2 | 1 | 1 | -1,5 | 0,5 | Peripherals |
| OTU0299 | 8 | 5 | 3 | 2 | -1,17299047 | 0,56 | Peripherals |
| OTU0172 | 7 | 3 | 1 | 2 | -1,10833761 | 0,66666667 | Connector |
| OTU0063 | 3 | 4 | 2 | 2 | -0,96656922 | 0,5 | Peripherals |
| OTU0171 | 1 | 3 | 3 | 0 | -1,3034821 | 0 | Peripherals |
| OTU0708 | 7 | 1 | 1 | 0 | -1,10833761 | 0 | Peripherals |
| OTU0173 | 1 | 2 | 1 | 1 | -1,52148607 | 0,5 | Peripherals |
| OTU0496 | 5 | 2 | 2 | 0 | -2,5 | 0 | Peripherals |

| | | | | | | | |
|---|---|---|---|---|---|---|---|
| OTU0522 | 1 | 2 | 2 | 0 | -1,41248408 | 0 | Peripherals |
| OTU0092 | 4 | 1 | 1 | 0 | -1,49255579 | 0 | Peripherals |
| OTU0801 | 3 | 1 | 1 | 0 | -1,28875896 | 0 | Peripherals |
| OTU0910 | 1 | 1 | 1 | 0 | -1,52148607 | 0 | Peripherals |
| OTU0112 | 1 | 1 | 1 | 0 | -1,52148607 | 0 | Peripherals |

**C** Placebo bath treatment:

| Node | Module | Edges All | Edges Inside Module | Edges Outside Module | Zi (within) | pi(outside) | Role |
|---|---|---|---|---|---|---|---|
| OTU0446 | 5 | 9 | 4 | 5 | -0,96164943 | 0,49382716 | Peripherals |
| OTU0907 | 6 | 15 | 14 | 1 | 0,66011525 | 0,12444444 | Peripherals |
| OTU0187 | 7 | 28 | 19 | 9 | -0,01834612 | 0,45663265 | Peripherals |
| OTU0192 | 7 | 34 | 26 | 8 | 0,68084479 | 0,35986159 | Peripherals |
| OTU0243 | 5 | 1 | 1 | 0 | -1,48938388 | 0 | Peripherals |
| OTU0714 | 5 | 7 | 3 | 4 | -1,13756092 | 0,65306122 | Connector |
| OTU0190 | 7 | 30 | 24 | 6 | 0,48107596 | 0,32 | Peripherals |
| OTU0892 | 10 | 16 | 16 | 0 | 1,32041575 | 0 | Peripherals |
| OTU0056 | 10 | 13 | 13 | 0 | 0,55017323 | 0 | Peripherals |
| OTU0462 | 7 | 13 | 9 | 4 | -1,01719027 | 0,4260355 | Peripherals |
| OTU0184 | 7 | 30 | 24 | 6 | 0,48107596 | 0,32 | Peripherals |
| OTU0830 | 7 | 38 | 25 | 13 | 0,58096038 | 0,48199446 | Peripherals |
| OTU0736 | 7 | 29 | 20 | 9 | 0,0815383 | 0,49702735 | Peripherals |
| OTU0569 | 7 | 25 | 23 | 2 | 0,38119155 | 0,1472 | Peripherals |
| OTU0174 | 6 | 18 | 16 | 2 | 1,01742534 | 0,19753086 | Peripherals |
| OTU0713 | 6 | 12 | 11 | 1 | 0,12415012 | 0,15277778 | Peripherals |
| OTU0712 | 6 | 16 | 15 | 1 | 0,83877029 | 0,1171875 | Peripherals |
| OTU0852 | 3 | 10 | 10 | 0 | 0,14968576 | 0 | Peripherals |
| OTU0647 | 3 | 11 | 11 | 0 | 0,33305081 | 0 | Peripherals |
| OTU0593 | 6 | 20 | 18 | 2 | 1,37473543 | 0,18 | Peripherals |
| OTU0804 | 6 | 16 | 15 | 1 | 0,83877029 | 0,1171875 | Peripherals |
| OTU0182 | 2 | 14 | 10 | 4 | 3,12042672 | 0,43877551 | Module Hub |
| OTU0371 | 3 | 13 | 12 | 1 | 0,51641586 | 0,14201183 | Peripherals |
| OTU0181 | 3 | 12 | 12 | 0 | 0,51641586 | 0 | Peripherals |
| OTU0176 | 10 | 19 | 18 | 1 | 1,83391077 | 0,09972299 | Peripherals |
| OTU0043 | 10 | 14 | 13 | 1 | 0,55017323 | 0,13265306 | Peripherals |
| OTU0573 | 10 | 12 | 12 | 0 | 0,29342572 | 0 | Peripherals |
| OTU0305 | 10 | 11 | 10 | 1 | -0,22006929 | 0,16528926 | Peripherals |
| OTU0831 | 3 | 8 | 5 | 3 | -0,7671395 | 0,46875 | Peripherals |
| OTU0435 | 3 | 10 | 10 | 0 | 0,14968576 | 0 | Peripherals |
| OTU0002 | 6 | 13 | 13 | 0 | 0,4814602 | 0 | Peripherals |
| OTU0889 | 6 | 20 | 17 | 3 | 1,19608038 | 0,27 | Peripherals |

| | | | | | | | |
|---|---|---|---|---|---|---|---|
| OTU0524 | 10 | 14 | 12 | 2 | 0,29342572 | 0,25510204 | Peripherals |
| OTU0458 | 10 | 15 | 14 | 1 | 0,80692074 | 0,12444444 | Peripherals |
| OTU0577 | 7 | 29 | 25 | 4 | 0,58096038 | 0,24494649 | Peripherals |
| OTU0649 | 7 | 28 | 27 | 1 | 0,78072921 | 0,06887755 | Peripherals |
| OTU0359 | 7 | 40 | 34 | 6 | 1,47992012 | 0,26125 | Peripherals |
| OTU0093 | 7 | 17 | 15 | 2 | -0,41788378 | 0,20761246 | Peripherals |
| OTU0258 | 3 | 21 | 11 | 10 | 0,33305081 | 0,49886621 | Peripherals |
| OTU0906 | 3 | 9 | 6 | 3 | -0,58377445 | 0,44444444 | Peripherals |
| OTU0007 | 3 | 12 | 12 | 0 | 0,51641586 | 0 | Peripherals |
| OTU0217 | 3 | 23 | 16 | 7 | 1,24987606 | 0,42344045 | Peripherals |
| OTU0744 | 2 | 10 | 9 | 1 | 2,64442943 | 0,18 | Module Hub |
| OTU0421 | 2 | 4 | 3 | 1 | -0,21155435 | 0,375 | Peripherals |
| OTU0423 | 6 | 17 | 17 | 0 | 1,19608038 | 0 | Peripherals |
| OTU0390 | 6 | 15 | 15 | 0 | 0,83877029 | 0 | Peripherals |
| OTU0245 | 6 | 17 | 17 | 0 | 1,19608038 | 0 | Peripherals |
| OTU0119 | 6 | 33 | 31 | 2 | 3,697251 | 0,11570248 | Module Hub |
| OTU0652 | 3 | 12 | 7 | 5 | -0,4004094 | 0,48611111 | Peripherals |
| OTU0809 | 3 | 17 | 17 | 0 | 1,43324111 | 0 | Peripherals |
| OTU0215 | 3 | 24 | 23 | 1 | 2,53343142 | 0,07986111 | Module Hub |
| OTU0698 | 3 | 6 | 4 | 2 | -0,95050455 | 0,5 | Peripherals |
| OTU0052 | 3 | 8 | 7 | 1 | -0,4004094 | 0,21875 | Peripherals |
| OTU0400 | 6 | 13 | 12 | 1 | 0,30280516 | 0,14201183 | Peripherals |
| OTU0769 | 6 | 14 | 14 | 0 | 0,66011525 | 0 | Peripherals |
| OTU0644 | 3 | 30 | 11 | 19 | 0,33305081 | 0,50444444 | Peripherals |
| OTU0069 | 7 | 20 | 14 | 6 | -0,5177682 | 0,445 | Peripherals |
| OTU0399 | 7 | 32 | 29 | 3 | 0,98049804 | 0,16992188 | Peripherals |
| OTU0807 | 7 | 5 | 5 | 0 | -1,41672794 | 0 | Peripherals |
| OTU0028 | 7 | 39 | 27 | 12 | 0,78072921 | 0,44049967 | Peripherals |
| OTU0416 | 7 | 22 | 17 | 5 | -0,21811495 | 0,38429752 | Peripherals |
| OTU0535 | 6 | 3 | 2 | 1 | -1,48374528 | 0,44444444 | Peripherals |
| OTU0584 | 6 | 5 | 5 | 0 | -0,94778015 | 0 | Peripherals |
| OTU0358 | 10 | 11 | 11 | 0 | 0,03667822 | 0 | Peripherals |
| OTU0016 | 10 | 9 | 9 | 0 | -0,4768168 | 0 | Peripherals |
| OTU0654 | 7 | 23 | 18 | 5 | -0,11823053 | 0,35538752 | Peripherals |
| OTU0937 | 3 | 14 | 14 | 0 | 0,88314596 | 0 | Peripherals |
| OTU0538 | 3 | 29 | 15 | 14 | 1,06651101 | 0,49940547 | Peripherals |
| OTU0107 | 6 | 16 | 13 | 3 | 0,4814602 | 0,328125 | Peripherals |
| OTU0137 | 7 | 46 | 35 | 11 | 1,57980453 | 0,37334594 | Peripherals |
| OTU0333 | 3 | 37 | 11 | 26 | 0,33305081 | 0,41782323 | Peripherals |
| OTU0330 | 7 | 24 | 14 | 10 | -0,5177682 | 0,60763889 | Peripherals |
| OTU0642 | 3 | 15 | 12 | 3 | 0,51641586 | 0,32 | Peripherals |
| OTU0724 | 3 | 14 | 13 | 1 | 0,69978091 | 0,13265306 | Peripherals |

| | | | | | | | |
|---|---|---|---|---|---|---|---|
| OTU0240 | 1 | 7 | 6 | 1 | 0,46551508 | 0,24489796 | Peripherals |
| OTU0329 | 1 | 7 | 6 | 1 | 0,46551508 | 0,24489796 | Peripherals |
| OTU0519 | 8 | 9 | 5 | 4 | 0,02103516 | 0,61728395 | Peripherals |
| OTU0061 | 8 | 8 | 6 | 2 | 0,42070316 | 0,40625 | Peripherals |
| OTU0585 | 2 | 8 | 5 | 3 | 0,74044024 | 0,46875 | Peripherals |
| OTU0490 | 3 | 9 | 4 | 5 | -0,95050455 | 0,49382716 | Peripherals |
| OTU0175 | 3 | 17 | 17 | 0 | 1,43324111 | 0 | Peripherals |
| OTU0319 | 3 | 17 | 17 | 0 | 1,43324111 | 0 | Peripherals |
| OTU0433 | 5 | 15 | 15 | 0 | 0,97337687 | 0 | Peripherals |
| OTU0449 | 5 | 13 | 13 | 0 | 0,6215539 | 0 | Peripherals |
| OTU0499 | 6 | 9 | 4 | 5 | -1,12643519 | 0,66666667 | Connector |
| OTU0276 | 6 | 5 | 3 | 2 | -1,30509024 | 0,56 | Peripherals |
| OTU0930 | 3 | 8 | 7 | 1 | -0,4004094 | 0,21875 | Peripherals |
| OTU0186 | 3 | 12 | 10 | 2 | 0,14968576 | 0,27777778 | Peripherals |
| OTU0159 | 1 | 4 | 4 | 0 | -0,34913631 | 0 | Peripherals |
| OTU0241 | 1 | 7 | 7 | 0 | 0,87284077 | 0 | Peripherals |
| OTU0075 | 7 | 21 | 19 | 2 | -0,01834612 | 0,1723356 | Peripherals |
| OTU0764 | 3 | 31 | 18 | 13 | 1,61660616 | 0,53485952 | Peripherals |
| OTU0553 | 7 | 36 | 33 | 3 | 1,3800357 | 0,15277778 | Peripherals |
| OTU0145 | 7 | 32 | 25 | 7 | 0,58096038 | 0,34179688 | Peripherals |
| OTU0375 | 6 | 14 | 14 | 0 | 0,66011525 | 0 | Peripherals |
| OTU0440 | 5 | 19 | 19 | 0 | 1,67702279 | 0 | Peripherals |
| OTU0528 | 3 | 7 | 6 | 1 | -0,58377445 | 0,24489796 | Peripherals |
| OTU0476 | 7 | 38 | 30 | 8 | 1,08038245 | 0,34210526 | Peripherals |
| OTU0754 | 3 | 6 | 3 | 3 | -1,1338696 | 0,66666667 | Connector |
| OTU0911 | 2 | 3 | 1 | 2 | -1,16354895 | 0,44444444 | Peripherals |
| OTU0896 | 5 | 15 | 15 | 0 | 0,97337687 | 0 | Peripherals |
| OTU0575 | 10 | 16 | 16 | 0 | 1,32041575 | 0 | Peripherals |
| OTU0817 | 6 | 17 | 16 | 1 | 1,01742534 | 0,11072664 | Peripherals |
| OTU0902 | 6 | 12 | 11 | 1 | 0,12415012 | 0,15277778 | Peripherals |
| OTU0122 | 6 | 13 | 11 | 2 | 0,12415012 | 0,27218935 | Peripherals |
| OTU0374 | 10 | 10 | 8 | 2 | -0,73356431 | 0,32 | Peripherals |
| OTU0740 | 6 | 18 | 17 | 1 | 1,19608038 | 0,10493827 | Peripherals |
| OTU0438 | 5 | 14 | 14 | 0 | 0,79746538 | 0 | Peripherals |
| OTU0745 | 6 | 10 | 9 | 1 | -0,23315997 | 0,18 | Peripherals |
| OTU0437 | 5 | 8 | 8 | 0 | -0,25800351 | 0 | Peripherals |
| OTU0921 | 7 | 28 | 26 | 2 | 0,68084479 | 0,13265306 | Peripherals |
| OTU0369 | 3 | 17 | 15 | 2 | 1,06651101 | 0,21453287 | Peripherals |
| OTU0133 | 3 | 17 | 17 | 0 | 1,43324111 | 0 | Peripherals |
| OTU0140 | 7 | 5 | 5 | 0 | -1,41672794 | 0 | Peripherals |
| OTU0432 | 5 | 15 | 15 | 0 | 0,97337687 | 0 | Peripherals |
| OTU0493 | 3 | 14 | 14 | 0 | 0,88314596 | 0 | Peripherals |

| | | | | | | | |
|---|---|---|---|---|---|---|---|
| OTU0285 | 1 | 8 | 8 | 0 | 1,28016647 | 0 | Peripherals |
| OTU0296 | 1 | 5 | 5 | 0 | 0,05818938 | 0 | Peripherals |
| OTU0618 | 7 | 15 | 14 | 1 | -0,5177682 | 0,12444444 | Peripherals |
| OTU0762 | 10 | 11 | 9 | 2 | -0,4768168 | 0,31404959 | Peripherals |
| OTU0328 | 10 | 13 | 11 | 2 | 0,03667822 | 0,27218935 | Peripherals |
| OTU0878 | 3 | 20 | 20 | 0 | 1,98333627 | 0 | Peripherals |
| OTU0273 | 5 | 18 | 18 | 0 | 1,50111131 | 0 | Peripherals |
| OTU0200 | 3 | 3 | 2 | 1 | -1,31723465 | 0,44444444 | Peripherals |
| OTU0456 | 3 | 14 | 14 | 0 | 0,88314596 | 0 | Peripherals |
| OTU0108 | 6 | 9 | 7 | 2 | -0,59047006 | 0,37037037 | Peripherals |
| OTU0628 | 10 | 12 | 12 | 0 | 0,29342572 | 0 | Peripherals |
| OTU0795 | 8 | 1 | 1 | 0 | -1,57763686 | 0 | Peripherals |
| OTU0370 | 8 | 7 | 7 | 0 | 0,82037117 | 0 | Peripherals |
| OTU0038 | 7 | 31 | 28 | 3 | 0,88061362 | 0,1748179 | Peripherals |
| OTU0755 | 6 | 15 | 15 | 0 | 0,83877029 | 0 | Peripherals |
| OTU0730 | 7 | 9 | 7 | 2 | -1,2169591 | 0,34567901 | Peripherals |
| OTU0334 | 7 | 9 | 5 | 4 | -1,41672794 | 0,61728395 | Peripherals |
| OTU0111 | 1 | 8 | 8 | 0 | 1,28016647 | 0 | Peripherals |
| OTU0125 | 6 | 8 | 4 | 4 | -1,12643519 | 0,59375 | Peripherals |
| OTU0910 | 7 | 33 | 30 | 3 | 1,08038245 | 0,16896235 | Peripherals |
| OTU0629 | 5 | 15 | 15 | 0 | 0,97337687 | 0 | Peripherals |
| OTU0903 | 5 | 11 | 10 | 1 | 0,09381946 | 0,16528926 | Peripherals |
| OTU0790 | 3 | 11 | 10 | 1 | 0,14968576 | 0,16528926 | Peripherals |
| OTU0091 | 7 | 13 | 5 | 8 | -1,41672794 | 0,55621302 | Peripherals |
| OTU0365 | 10 | 9 | 5 | 4 | -1,50380683 | 0,56790123 | Peripherals |
| OTU0387 | 3 | 14 | 14 | 0 | 0,88314596 | 0 | Peripherals |
| OTU0073 | 6 | 14 | 14 | 0 | 0,66011525 | 0 | Peripherals |
| OTU0605 | 6 | 10 | 10 | 0 | -0,05450493 | 0 | Peripherals |
| OTU0540 | 7 | 28 | 26 | 2 | 0,68084479 | 0,13520408 | Peripherals |
| OTU0036 | 3 | 17 | 14 | 3 | 0,88314596 | 0,29065744 | Peripherals |
| OTU0242 | 3 | 7 | 7 | 0 | -0,4004094 | 0 | Peripherals |
| OTU0733 | 6 | 18 | 10 | 8 | -0,05450493 | 0,61111111 | Peripherals |
| OTU0603 | 8 | 7 | 4 | 3 | -0,37863285 | 0,57142857 | Peripherals |
| OTU0895 | 3 | 12 | 12 | 0 | 0,51641586 | 0 | Peripherals |
| OTU0126 | 6 | 17 | 17 | 0 | 1,19608038 | 0 | Peripherals |
| OTU0706 | 1 | 7 | 7 | 0 | 0,87284077 | 0 | Peripherals |
| OTU0756 | 1 | 6 | 6 | 0 | 0,46551508 | 0 | Peripherals |
| OTU0279 | 7 | 43 | 33 | 10 | 1,3800357 | 0,3569497 | Peripherals |
| OTU0198 | 7 | 29 | 26 | 3 | 0,68084479 | 0,18549346 | Peripherals |
| OTU0336 | 2 | 2 | 2 | 0 | -0,68755165 | 0 | Peripherals |
| OTU0789 | 2 | 4 | 4 | 0 | 0,26444294 | 0 | Peripherals |
| OTU0461 | 3 | 15 | 14 | 1 | 0,88314596 | 0,12444444 | Peripherals |

| | | | | | | | |
|---|---|---|---|---|---|---|---|
| OTU0512 | 7 | 29 | 27 | 2 | 0,78072921 | 0,12841855 | Peripherals |
| OTU0455 | 7 | 3 | 2 | 1 | -1,71638118 | 0,44444444 | Peripherals |
| OTU0692 | 6 | 10 | 5 | 5 | -0,94778015 | 0,64 | Connector |
| OTU0509 | 3 | 18 | 18 | 0 | 1,61660616 | 0 | Peripherals |
| OTU0405 | 7 | 15 | 10 | 5 | -0,91730586 | 0,48 | Peripherals |
| OTU0673 | 3 | 24 | 10 | 14 | 0,14968576 | 0,48611111 | Peripherals |
| OTU0022 | 7 | 33 | 24 | 9 | 0,48107596 | 0,39669421 | Peripherals |
| OTU0396 | 7 | 10 | 5 | 5 | -1,41672794 | 0,66 | Connector |
| OTU0859 | 3 | 15 | 12 | 3 | 0,51641586 | 0,32 | Peripherals |
| OTU0671 | 2 | 6 | 6 | 0 | 1,21643754 | 0 | Peripherals |
| OTU0670 | 2 | 4 | 3 | 1 | -0,21155435 | 0,375 | Peripherals |
| OTU0398 | 3 | 5 | 4 | 1 | -0,95050455 | 0,32 | Peripherals |
| OTU0265 | 7 | 35 | 33 | 2 | 1,3800357 | 0,10938776 | Peripherals |
| OTU0078 | 3 | 16 | 15 | 1 | 1,06651101 | 0,1171875 | Peripherals |
| OTU0912 | 7 | 20 | 19 | 1 | -0,01834612 | 0,095 | Peripherals |
| OTU0317 | 7 | 2 | 2 | 0 | -1,71638118 | 0 | Peripherals |
| OTU0856 | 7 | 22 | 20 | 2 | 0,0815383 | 0,16528926 | Peripherals |
| OTU0836 | 3 | 20 | 9 | 11 | -0,0336793 | 0,67 | Connector |
| OTU0427 | 2 | 7 | 2 | 5 | -0,68755165 | 0,69387755 | Connector |
| OTU0597 | 6 | 15 | 15 | 0 | 0,83877029 | 0 | Peripherals |
| OTU0295 | 6 | 8 | 8 | 0 | -0,41181502 | 0 | Peripherals |
| OTU0492 | 3 | 15 | 15 | 0 | 1,06651101 | 0 | Peripherals |
| OTU0633 | 3 | 14 | 14 | 0 | 0,88314596 | 0 | Peripherals |
| OTU0231 | 3 | 9 | 9 | 0 | -0,0336793 | 0 | Peripherals |
| OTU0006 | 6 | 13 | 13 | 0 | 0,4814602 | 0 | Peripherals |
| OTU0806 | 6 | 10 | 9 | 1 | -0,23315997 | 0,18 | Peripherals |
| OTU0274 | 10 | 12 | 11 | 1 | 0,03667822 | 0,15277778 | Peripherals |
| OTU0483 | 10 | 12 | 12 | 0 | 0,29342572 | 0 | Peripherals |
| OTU0842 | 1 | 8 | 8 | 0 | 1,28016647 | 0 | Peripherals |
| OTU0866 | 1 | 3 | 3 | 0 | -0,756462 | 0 | Peripherals |
| OTU0029 | 8 | 13 | 6 | 7 | 0,42070316 | 0,69822485 | Connector |
| OTU0134 | 3 | 5 | 3 | 2 | -1,1338696 | 0,56 | Peripherals |
| OTU0127 | 3 | 2 | 2 | 0 | -1,31723465 | 0 | Peripherals |
| OTU0124 | 3 | 4 | 4 | 0 | -0,95050455 | 0 | Peripherals |
| OTU0084 | 6 | 8 | 8 | 0 | -0,41181502 | 0 | Peripherals |
| OTU0311 | 8 | 6 | 6 | 0 | 0,42070316 | 0 | Peripherals |
| OTU0123 | 8 | 4 | 3 | 1 | -0,77830085 | 0,375 | Peripherals |
| OTU0100 | 1 | 4 | 2 | 2 | -1,1637877 | 0,625 | Connector |
| OTU0514 | 1 | 4 | 3 | 1 | -0,756462 | 0,375 | Peripherals |
| OTU0429 | 3 | 11 | 11 | 0 | 0,33305081 | 0 | Peripherals |
| OTU0081 | 3 | 9 | 9 | 0 | -0,0336793 | 0 | Peripherals |
| OTU0933 | 6 | 3 | 3 | 0 | -1,30509024 | 0 | Peripherals |

| | | | | | | | |
|---|---|---|---|---|---|---|---|
| OTU0641 | 6 | 6 | 3 | 3 | -1,30509024 | 0,61111111 | Peripherals |
| OTU0381 | 2 | 4 | 3 | 1 | -0,21155435 | 0,375 | Peripherals |
| OTU0899 | 2 | 6 | 6 | 0 | 1,21643754 | 0 | Peripherals |
| OTU0169 | 3 | 4 | 3 | 1 | -1,1338696 | 0,375 | Peripherals |
| OTU0373 | 7 | 28 | 24 | 4 | 0,48107596 | 0,24489796 | Peripherals |
| OTU0430 | 3 | 17 | 17 | 0 | 1,43324111 | 0 | Peripherals |
| OTU0383 | 3 | 3 | 3 | 0 | -1,1338696 | 0 | Peripherals |
| OTU0697 | 5 | 12 | 12 | 0 | 0,44564242 | 0 | Peripherals |
| OTU0635 | 10 | 4 | 3 | 1 | -2,01730184 | 0,375 | Peripherals |
| OTU0626 | 5 | 13 | 13 | 0 | 0,6215539 | 0 | Peripherals |
| OTU0201 | 8 | 1 | 1 | 0 | -1,57763686 | 0 | Peripherals |
| OTU0925 | 8 | 4 | 2 | 2 | -1,17796885 | 0,625 | Connector |
| OTU0510 | 3 | 10 | 10 | 0 | 0,14968576 | 0 | Peripherals |
| OTU0486 | 3 | 17 | 11 | 6 | 0,33305081 | 0,53979239 | Peripherals |
| OTU0489 | 3 | 22 | 19 | 3 | 1,79997121 | 0,23553719 | Peripherals |
| OTU0053 | 3 | 2 | 2 | 0 | -1,31723465 | 0 | Peripherals |
| OTU0197 | 6 | 10 | 9 | 1 | -0,23315997 | 0,18 | Peripherals |
| OTU0885 | 3 | 2 | 2 | 0 | -1,31723465 | 0 | Peripherals |
| OTU0607 | 3 | 4 | 4 | 0 | -0,95050455 | 0 | Peripherals |
| OTU0600 | 3 | 10 | 8 | 2 | -0,21704435 | 0,34 | Peripherals |
| OTU0304 | 8 | 7 | 5 | 2 | 0,02103516 | 0,40816327 | Peripherals |
| OTU0897 | 5 | 13 | 13 | 0 | 0,6215539 | 0 | Peripherals |
| OTU0183 | 6 | 15 | 15 | 0 | 0,83877029 | 0 | Peripherals |
| OTU0142 | 6 | 14 | 14 | 0 | 0,66011525 | 0 | Peripherals |
| OTU0922 | 8 | 5 | 4 | 1 | -0,37863285 | 0,32 | Peripherals |
| OTU0039 | 6 | 8 | 6 | 2 | -0,76912511 | 0,40625 | Peripherals |
| OTU0822 | 2 | 9 | 3 | 6 | -0,21155435 | 0,64197531 | Connector |
| OTU0452 | 3 | 3 | 3 | 0 | -1,1338696 | 0 | Peripherals |
| OTU0412 | 3 | 12 | 12 | 0 | 0,51641586 | 0 | Peripherals |
| OTU0237 | 5 | 11 | 9 | 2 | -0,08209202 | 0,29752066 | Peripherals |
| OTU0082 | 5 | 17 | 17 | 0 | 1,32519983 | 0 | Peripherals |
| OTU0411 | 6 | 15 | 14 | 1 | 0,66011525 | 0,12444444 | Peripherals |
| OTU0269 | 8 | 7 | 6 | 1 | 0,42070316 | 0,24489796 | Peripherals |
| OTU0484 | 3 | 4 | 4 | 0 | -0,95050455 | 0 | Peripherals |
| OTU0828 | 3 | 12 | 12 | 0 | 0,51641586 | 0 | Peripherals |
| OTU0505 | 3 | 13 | 13 | 0 | 0,69978091 | 0 | Peripherals |
| OTU0150 | 3 | 12 | 9 | 3 | -0,0336793 | 0,375 | Peripherals |
| OTU0737 | 3 | 8 | 8 | 0 | -0,21704435 | 0 | Peripherals |
| OTU0522 | 10 | 11 | 11 | 0 | 0,03667822 | 0 | Peripherals |
| OTU0482 | 2 | 2 | 2 | 0 | -0,68755165 | 0 | Peripherals |
| OTU0900 | 2 | 7 | 6 | 1 | 1,21643754 | 0,24489796 | Peripherals |
| OTU0049 | 6 | 7 | 6 | 1 | -0,76912511 | 0,24489796 | Peripherals |

| | | | | | | | |
|---|---|---|---|---|---|---|---|
| OTU0154 | 2 | 4 | 3 | 1 | -0,21155435 | 0,375 | Peripherals |
| OTU0719 | 2 | 3 | 3 | 0 | -0,21155435 | 0 | Peripherals |
| OTU0167 | 3 | 15 | 11 | 4 | 0,33305081 | 0,42666667 | Peripherals |
| OTU0042 | 3 | 17 | 17 | 0 | 1,43324111 | 0 | Peripherals |
| OTU0705 | 5 | 7 | 7 | 0 | -0,43391499 | 0 | Peripherals |
| OTU0624 | 1 | 5 | 2 | 3 | -1,1637877 | 0,48 | Peripherals |
| OTU0051 | 6 | 13 | 12 | 1 | 0,30280516 | 0,14201183 | Peripherals |
| OTU0936 | 2 | 3 | 3 | 0 | -0,21155435 | 0 | Peripherals |
| OTU0099 | 5 | 14 | 13 | 1 | 0,6215539 | 0,13265306 | Peripherals |
| OTU0748 | 7 | 2 | 2 | 0 | -1,71638118 | 0 | Peripherals |
| OTU0283 | 5 | 17 | 17 | 0 | 1,32519983 | 0 | Peripherals |
| OTU0542 | 3 | 28 | 17 | 11 | 1,43324111 | 0,47704082 | Peripherals |
| OTU0516 | 3 | 29 | 16 | 13 | 1,24987606 | 0,52318668 | Peripherals |
| OTU0669 | 3 | 9 | 7 | 2 | -0,4004094 | 0,34567901 | Peripherals |
| OTU0604 | 6 | 9 | 8 | 1 | -0,41181502 | 0,19753086 | Peripherals |
| OTU0466 | 3 | 9 | 9 | 0 | -0,0336793 | 0 | Peripherals |
| OTU0761 | 8 | 1 | 1 | 0 | -1,57763686 | 0 | Peripherals |
| OTU0092 | 6 | 8 | 6 | 2 | -0,76912511 | 0,375 | Peripherals |
| OTU0825 | 6 | 7 | 5 | 2 | -0,94778015 | 0,40816327 | Peripherals |
| OTU0065 | 6 | 7 | 5 | 2 | -0,94778015 | 0,40816327 | Peripherals |
| OTU0220 | 3 | 4 | 4 | 0 | -0,95050455 | 0 | Peripherals |
| OTU0407 | 3 | 9 | 9 | 0 | -0,0336793 | 0 | Peripherals |
| OTU0335 | 1 | 10 | 5 | 5 | 0,05818938 | 0,66 | Connector |
| OTU0026 | 5 | 4 | 4 | 0 | -0,96164943 | 0 | Peripherals |
| OTU0447 | 5 | 11 | 9 | 2 | -0,08209202 | 0,29752066 | Peripherals |
| OTU0630 | 10 | 2 | 2 | 0 | -2,27404935 | 0 | Peripherals |
| OTU0543 | 7 | 25 | 24 | 1 | 0,48107596 | 0,0768 | Peripherals |
| OTU0537 | 6 | 6 | 4 | 2 | -1,12643519 | 0,5 | Peripherals |
| OTU0442 | 2 | 4 | 3 | 1 | -0,21155435 | 0,375 | Peripherals |
| OTU0494 | 2 | 6 | 2 | 4 | -0,68755165 | 0,44444444 | Peripherals |
| OTU0708 | 3 | 5 | 2 | 3 | -1,31723465 | 0,64 | Connector |
| OTU0340 | 8 | 6 | 6 | 0 | 0,42070316 | 0 | Peripherals |
| OTU0259 | 8 | 19 | 11 | 8 | 2,41904318 | 0,59279778 | Peripherals |
| OTU0264 | 8 | 8 | 6 | 2 | 0,42070316 | 0,375 | Peripherals |
| OTU0602 | 3 | 5 | 4 | 1 | -0,95050455 | 0,32 | Peripherals |
| OTU0893 | 5 | 3 | 3 | 0 | -1,13756092 | 0 | Peripherals |
| OTU0275 | 1 | 9 | 9 | 0 | 1,68749216 | 0 | Peripherals |
| OTU0420 | 3 | 14 | 14 | 0 | 0,88314596 | 0 | Peripherals |
| OTU0074 | 6 | 10 | 9 | 1 | -0,23315997 | 0,18 | Peripherals |
| OTU0235 | 1 | 3 | 2 | 1 | -1,1637877 | 0,44444444 | Peripherals |
| OTU0743 | 1 | 7 | 6 | 1 | 0,46551508 | 0,24489796 | Peripherals |
| OTU0592 | 8 | 8 | 8 | 0 | 1,22003917 | 0 | Peripherals |

| | | | | | | | |
|---|---|---|---|---|---|---|---|
| OTU0909 | 2 | 4 | 4 | 0 | 0,26444294 | 0 | Peripherals |
| OTU0165 | 2 | 4 | 4 | 0 | 0,26444294 | 0 | Peripherals |
| OTU0199 | 3 | 11 | 5 | 6 | -0,7671395 | 0,67768595 | Connector |
| OTU0448 | 2 | 2 | 2 | 0 | -0,68755165 | 0 | Peripherals |
| OTU0742 | 1 | 3 | 3 | 0 | -0,756462 | 0 | Peripherals |
| OTU0352 | 6 | 7 | 4 | 3 | -1,12643519 | 0,57142857 | Peripherals |
| OTU0318 | 5 | 7 | 4 | 3 | -0,96164943 | 0,48979592 | Peripherals |
| OTU0177 | 6 | 9 | 9 | 0 | -0,23315997 | 0 | Peripherals |
| OTU0148 | 2 | 4 | 4 | 0 | 0,26444294 | 0 | Peripherals |
| OTU0086 | 2 | 4 | 4 | 0 | 0,26444294 | 0 | Peripherals |
| OTU0366 | 2 | 4 | 4 | 0 | 0,26444294 | 0 | Peripherals |
| OTU0410 | 7 | 9 | 4 | 5 | -1,51661235 | 0,69135802 | Connector |
| OTU0057 | 8 | 8 | 6 | 2 | 0,42070316 | 0,375 | Peripherals |
| OTU0189 | 3 | 5 | 5 | 0 | -0,7671395 | 0 | Peripherals |
| OTU0203 | 3 | 2 | 2 | 0 | -1,31723465 | 0 | Peripherals |
| OTU0525 | 6 | 1 | 1 | 0 | -1,66240033 | 0 | Peripherals |
| OTU0234 | 2 | 5 | 3 | 2 | -0,21155435 | 0,48 | Peripherals |
| OTU0014 | 3 | 1 | 1 | 0 | -1,5005997 | 0 | Peripherals |
| OTU0663 | 3 | 3 | 3 | 0 | -1,1338696 | 0 | Peripherals |
| OTU0814 | 2 | 2 | 1 | 1 | -1,16354895 | 0,5 | Peripherals |
| OTU0050 | 3 | 2 | 2 | 0 | -1,31723465 | 0 | Peripherals |
| OTU0298 | 2 | 3 | 3 | 0 | -0,21155435 | 0 | Peripherals |
| OTU0292 | 2 | 2 | 2 | 0 | -0,68755165 | 0 | Peripherals |
| OTU0536 | 2 | 8 | 7 | 1 | 1,69243483 | 0,21875 | Peripherals |
| OTU0367 | 6 | 5 | 5 | 0 | -0,94778015 | 0 | Peripherals |
| OTU0570 | 3 | 4 | 4 | 0 | -0,95050455 | 0 | Peripherals |
| OTU0684 | 7 | 2 | 2 | 0 | -1,71638118 | 0 | Peripherals |
| OTU0088 | 2 | 3 | 2 | 1 | -0,68755165 | 0,44444444 | Peripherals |
| OTU0610 | 5 | 3 | 3 | 0 | -1,13756092 | 0 | Peripherals |
| OTU0801 | 5 | 2 | 2 | 0 | -1,3134724 | 0 | Peripherals |
| OTU0431 | 6 | 3 | 3 | 0 | -1,30509024 | 0 | Peripherals |
| OTU0478 | 2 | 1 | 1 | 0 | -1,16354895 | 0 | Peripherals |
| OTU0138 | 3 | 4 | 4 | 0 | -0,95050455 | 0 | Peripherals |
| OTU0299 | 2 | 4 | 2 | 2 | -0,68755165 | 0,5 | Peripherals |
| OTU0480 | 3 | 1 | 1 | 0 | -1,5005997 | 0 | Peripherals |
| OTU0168 | 3 | 2 | 1 | 1 | -1,5005997 | 0,5 | Peripherals |
| OTU0178 | 5 | 4 | 4 | 0 | -0,96164943 | 0 | Peripherals |
| OTU0891 | 1 | 1 | 1 | 0 | -1,57111339 | 0 | Peripherals |
| OTU0474 | 3 | 3 | 3 | 0 | -1,1338696 | 0 | Peripherals |
| OTU0717 | 3 | 1 | 1 | 0 | -1,5005997 | 0 | Peripherals |
| OTU0572 | 6 | 2 | 2 | 0 | -1,48374528 | 0 | Peripherals |
| OTU0465 | 3 | 6 | 6 | 0 | -0,58377445 | 0 | Peripherals |

| | | | | | | | |
|---|---|---|---|---|---|---|---|
| OTU0812 | 5 | 2 | 2 | 0 | -1,3134724 | 0 | Peripherals |
| OTU0315 | 5 | 2 | 2 | 0 | -1,3134724 | 0 | Peripherals |
| OTU0612 | 3 | 2 | 2 | 0 | -1,31723465 | 0 | Peripherals |
| OTU0511 | 2 | 1 | 1 | 0 | -1,16354895 | 0 | Peripherals |
| OTU0621 | 3 | 1 | 1 | 0 | -1,5005997 | 0 | Peripherals |
| OTU0932 | 2 | 1 | 1 | 0 | -1,16354895 | 0 | Peripherals |
| OTU0314 | 1 | 1 | 1 | 0 | -1,57111339 | 0 | Peripherals |

**D** merged network with the differences in the topological role between all three bath treatments

| Node | Module | Edges | | | Zi (within) | pi(outside) | Rolle |
|---|---|---|---|---|---|---|---|
| | | All | Inside Module | Outside Module | | | |
| OTU0932 | 1 | 1 | 1 | 0 | -1,17260394 | 0 | Peripherals |
| OTU0511 | 1 | 1 | 1 | 0 | -1,17260394 | 0 | Peripherals |
| OTU0612 | 3 | 2 | 2 | 0 | -1,26267274 | 0 | Peripherals |
| OTU0717 | 3 | 1 | 1 | 0 | -1,45077 | 0 | Peripherals |
| OTU0801 | 4 | 2 | 2 | 0 | -1,37919905 | 0 | Peripherals |
| OTU0610 | 4 | 3 | 3 | 0 | -1,1523571 | 0 | Peripherals |
| OTU0088 | 1 | 3 | 2 | 1 | -0,63960215 | 0,44444444 | Peripherals |
| OTU0684 | 5 | 2 | 2 | 0 | -1,74843654 | 0 | Peripherals |
| OTU0367 | 6 | 5 | 5 | 0 | -0,81843962 | 0 | Peripherals |
| OTU0480 | 3 | 1 | 1 | 0 | -1,45077 | 0 | Peripherals |
| OTU0663 | 3 | 3 | 3 | 0 | -1,07457547 | 0 | Peripherals |
| OTU0014 | 3 | 1 | 1 | 0 | -1,45077 | 0 | Peripherals |
| OTU0234 | 7 | 5 | 3 | 2 | -0,72140842 | 0,48 | Peripherals |
| OTU0742 | 8 | 3 | 3 | 0 | -0,34956332 | 0 | Peripherals |
| OTU0298 | 1 | 3 | 3 | 0 | -0,10660036 | 0 | Peripherals |
| OTU0165 | 1 | 4 | 4 | 0 | 0,42640143 | 0 | Peripherals |
| OTU0240 | 8 | 2 | 2 | 0 | -0,84893949 | 0 | Peripherals |
| OTU0743 | 8 | 7 | 6 | 1 | 1,14856519 | 0,24489796 | Peripherals |
| OTU0893 | 4 | 1 | 1 | 0 | -1,606041 | 0 | Peripherals |
| OTU0442 | 7 | 4 | 3 | 1 | -0,72140842 | 0,375 | Peripherals |
| OTU0447 | 6 | 5 | 2 | 3 | -1,35579897 | 0,48 | Peripherals |
| OTU0178 | 4 | 4 | 4 | 0 | -0,92551515 | 0 | Peripherals |
| OTU0936 | 1 | 3 | 3 | 0 | -0,10660036 | 0 | Peripherals |
| OTU0292 | 1 | 2 | 2 | 0 | -0,63960215 | 0 | Peripherals |
| OTU0719 | 1 | 3 | 3 | 0 | -0,10660036 | 0 | Peripherals |
| OTU0482 | 1 | 2 | 2 | 0 | -0,63960215 | 0 | Peripherals |
| OTU0026 | 4 | 3 | 3 | 0 | -1,1523571 | 0 | Peripherals |
| OTU0412 | 3 | 12 | 12 | 0 | 0,61829992 | 0 | Peripherals |
| OTU0897 | 4 | 11 | 11 | 0 | 0,66237849 | 0 | Peripherals |
| OTU0057 | 7 | 6 | 5 | 1 | 0,19674775 | 0,27777778 | Peripherals |

| | | | | | | | |
|---|---|---|---|---|---|---|---|
| OTU0304 | 7 | 7 | 5 | 2 | 0,19674775 | 0,40816327 | Peripherals |
| OTU0220 | 3 | 3 | 3 | 0 | -1,07457547 | 0 | Peripherals |
| OTU0053 | 3 | 2 | 2 | 0 | -1,26267274 | 0 | Peripherals |
| OTU0201 | 3 | 1 | 1 | 0 | -1,45077 | 0 | Peripherals |
| OTU0761 | 7 | 1 | 1 | 0 | -1,63956459 | 0 | Peripherals |
| OTU0123 | 7 | 4 | 3 | 1 | -0,72140842 | 0,375 | Peripherals |
| OTU0452 | 3 | 3 | 3 | 0 | -1,07457547 | 0 | Peripherals |
| OTU0127 | 3 | 2 | 2 | 0 | -1,26267274 | 0 | Peripherals |
| OTU0592 | 7 | 8 | 8 | 0 | 1,57398201 | 0 | Peripherals |
| OTU0269 | 7 | 6 | 5 | 1 | 0,19674775 | 0,27777778 | Peripherals |
| OTU0340 | 7 | 6 | 6 | 0 | 0,65582584 | 0 | Peripherals |
| OTU0039 | 6 | 8 | 7 | 1 | -0,46020006 | 0,21875 | Peripherals |
| OTU0029 | 7 | 12 | 7 | 5 | 1,11490392 | 0,58333333 | Peripherals |
| OTU0275 | 8 | 4 | 4 | 0 | 0,14981285 | 0 | Peripherals |
| OTU0600 | 3 | 8 | 6 | 2 | -0,51028367 | 0,40625 | Peripherals |
| OTU0885 | 3 | 2 | 2 | 0 | -1,26267274 | 0 | Peripherals |
| OTU0597 | 6 | 13 | 13 | 0 | 0,61451864 | 0 | Peripherals |
| OTU0448 | 7 | 2 | 2 | 0 | -1,1804865 | 0 | Peripherals |
| OTU0909 | 1 | 4 | 4 | 0 | 0,42640143 | 0 | Peripherals |
| OTU0352 | 6 | 6 | 4 | 2 | -0,9975594 | 0,5 | Peripherals |
| OTU0065 | 6 | 7 | 5 | 2 | -0,81843962 | 0,40816327 | Peripherals |
| OTU0126 | 6 | 17 | 16 | 1 | 1,15187799 | 0,11072664 | Peripherals |
| OTU0259 | 7 | 17 | 10 | 7 | 2,49213818 | 0,58131488 | Peripherals |
| OTU0603 | 7 | 6 | 4 | 2 | -0,26233033 | 0,5 | Peripherals |
| OTU0733 | 6 | 18 | 10 | 8 | 0,07715929 | 0,61111111 | Peripherals |
| OTU0933 | 6 | 3 | 3 | 0 | -1,17667919 | 0 | Peripherals |
| OTU0317 | 5 | 2 | 2 | 0 | -1,74843654 | 0 | Peripherals |
| OTU0334 | 5 | 9 | 5 | 4 | -1,42892529 | 0,61728395 | Peripherals |
| OTU0049 | 6 | 7 | 6 | 1 | -0,63931984 | 0,24489796 | Peripherals |
| OTU0755 | 6 | 14 | 14 | 0 | 0,79363842 | 0 | Peripherals |
| OTU0795 | 7 | 1 | 1 | 0 | -1,63956459 | 0 | Peripherals |
| OTU0525 | 6 | 1 | 1 | 0 | -1,53491875 | 0 | Peripherals |
| OTU0484 | 3 | 4 | 4 | 0 | -0,8864782 | 0 | Peripherals |
| OTU0189 | 3 | 4 | 4 | 0 | -0,8864782 | 0 | Peripherals |
| OTU0494 | 7 | 4 | 3 | 1 | -0,72140842 | 0,375 | Peripherals |
| OTU0641 | 6 | 5 | 3 | 2 | -1,17667919 | 0,48 | Peripherals |
| OTU0762 | 10 | 5 | 3 | 2 | -1,85696145 | 0,56 | Peripherals |
| OTU0231 | 3 | 9 | 9 | 0 | 0,05400813 | 0 | Peripherals |
| OTU0633 | 3 | 14 | 14 | 0 | 0,99449446 | 0 | Peripherals |
| OTU0708 | 3 | 5 | 2 | 3 | -1,26267274 | 0,64 | Connector |
| OTU0737 | 3 | 8 | 8 | 0 | -0,13408914 | 0 | Peripherals |
| OTU0125 | 3 | 7 | 4 | 3 | -0,8864782 | 0,48979592 | Peripherals |

| | | | | | | | |
|---|---|---|---|---|---|---|---|
| OTU0902 | 6 | 8 | 7 | 1 | -0,46020006 | 0,21875 | Peripherals |
| OTU0817 | 6 | 11 | 11 | 0 | 0,25627907 | 0 | Peripherals |
| OTU0283 | 4 | 8 | 8 | 0 | -0,01814736 | 0 | Peripherals |
| OTU0697 | 4 | 11 | 11 | 0 | 0,66237849 | 0 | Peripherals |
| OTU0099 | 4 | 8 | 7 | 1 | -0,24498931 | 0,21875 | Peripherals |
| OTU0896 | 4 | 11 | 11 | 0 | 0,66237849 | 0 | Peripherals |
| OTU0456 | 3 | 14 | 14 | 0 | 0,99449446 | 0 | Peripherals |
| OTU0754 | 3 | 6 | 3 | 3 | -1,07457547 | 0,66666667 | Connector |
| OTU0626 | 4 | 9 | 9 | 0 | 0,20869459 | 0 | Peripherals |
| OTU0629 | 4 | 10 | 10 | 0 | 0,43553654 | 0 | Peripherals |
| OTU0082 | 4 | 13 | 13 | 0 | 1,11606239 | 0 | Peripherals |
| OTU0440 | 4 | 12 | 10 | 2 | 0,43553654 | 0,27777778 | Peripherals |
| OTU0335 | 8 | 5 | 1 | 4 | -1,34831566 | 0,72 | Connector |
| OTU0706 | 8 | 7 | 7 | 0 | 1,64794136 | 0 | Peripherals |
| OTU0756 | 8 | 4 | 4 | 0 | 0,14981285 | 0 | Peripherals |
| OTU0866 | 8 | 3 | 3 | 0 | -0,34956332 | 0 | Peripherals |
| OTU0285 | 8 | 5 | 5 | 0 | 0,64918902 | 0 | Peripherals |
| OTU0329 | 8 | 2 | 2 | 0 | -0,84893949 | 0 | Peripherals |
| OTU0111 | 8 | 3 | 3 | 0 | -0,34956332 | 0 | Peripherals |
| OTU0241 | 8 | 7 | 7 | 0 | 1,64794136 | 0 | Peripherals |
| OTU0159 | 8 | 4 | 4 | 0 | 0,14981285 | 0 | Peripherals |
| OTU0237 | 4 | 11 | 8 | 3 | -0,01814736 | 0,42975207 | Peripherals |
| OTU0383 | 3 | 3 | 3 | 0 | -1,07457547 | 0 | Peripherals |
| OTU0050 | 3 | 2 | 2 | 0 | -1,26267274 | 0 | Peripherals |
| OTU0430 | 3 | 17 | 17 | 0 | 1,55878625 | 0 | Peripherals |
| OTU0930 | 3 | 8 | 7 | 1 | -0,32218641 | 0,21875 | Peripherals |
| OTU0100 | 8 | 4 | 2 | 2 | -0,84893949 | 0,625 | Connector |
| OTU0154 | 1 | 4 | 3 | 1 | -0,10660036 | 0,375 | Peripherals |
| OTU0572 | 6 | 2 | 2 | 0 | -1,35579897 | 0 | Peripherals |
| OTU0276 | 6 | 5 | 3 | 2 | -1,17667919 | 0,56 | Peripherals |
| OTU0903 | 4 | 11 | 10 | 1 | 0,43553654 | 0,16528926 | Peripherals |
| OTU0432 | 4 | 15 | 14 | 1 | 1,34290434 | 0,12444444 | Peripherals |
| OTU0437 | 4 | 6 | 5 | 1 | -0,6986732 | 0,27777778 | Peripherals |
| OTU0438 | 4 | 14 | 14 | 0 | 1,34290434 | 0 | Peripherals |
| OTU0433 | 4 | 10 | 9 | 1 | 0,20869459 | 0,18 | Peripherals |
| OTU0602 | 3 | 5 | 4 | 1 | -0,8864782 | 0,32 | Peripherals |
| OTU0899 | 7 | 6 | 6 | 0 | 0,65582584 | 0 | Peripherals |
| OTU0381 | 7 | 4 | 3 | 1 | -0,72140842 | 0,375 | Peripherals |
| OTU0585 | 7 | 8 | 5 | 3 | 0,19674775 | 0,46875 | Peripherals |
| OTU0427 | 7 | 6 | 5 | 1 | 0,19674775 | 0,27777778 | Peripherals |
| OTU0061 | 7 | 8 | 7 | 1 | 1,11490392 | 0,21875 | Peripherals |
| OTU0519 | 7 | 9 | 6 | 3 | 0,65582584 | 0,49382716 | Peripherals |

| | | | | | | | |
|---|---|---|---|---|---|---|---|
| OTU0242 | 3 | 7 | 7 | 0 | -0,32218641 | 0 | Peripherals |
| OTU0016 | 10 | 9 | 9 | 0 | -0,08842674 | 0 | Peripherals |
| OTU0584 | 6 | 5 | 5 | 0 | -0,81843962 | 0 | Peripherals |
| OTU0535 | 6 | 3 | 2 | 1 | -1,35579897 | 0,44444444 | Peripherals |
| OTU0499 | 6 | 7 | 5 | 2 | -0,81843962 | 0,40816327 | Peripherals |
| OTU0654 | 5 | 19 | 15 | 4 | -0,36388781 | 0,34903047 | Peripherals |
| OTU0807 | 5 | 5 | 5 | 0 | -1,42892529 | 0 | Peripherals |
| OTU0122 | 6 | 12 | 11 | 1 | 0,25627907 | 0,15277778 | Peripherals |
| OTU0730 | 5 | 8 | 6 | 2 | -1,32242154 | 0,375 | Peripherals |
| OTU0465 | 3 | 6 | 6 | 0 | -0,51028367 | 0 | Peripherals |
| OTU0570 | 3 | 4 | 4 | 0 | -0,8864782 | 0 | Peripherals |
| OTU0925 | 3 | 3 | 3 | 0 | -1,07457547 | 0 | Peripherals |
| OTU0764 | 3 | 28 | 15 | 13 | 1,18259172 | 0,55612245 | Peripherals |
| OTU0420 | 3 | 13 | 13 | 0 | 0,80639719 | 0 | Peripherals |
| OTU0489 | 3 | 22 | 19 | 3 | 1,93498078 | 0,23553719 | Peripherals |
| OTU0724 | 3 | 11 | 10 | 1 | 0,24210539 | 0,16528926 | Peripherals |
| OTU0492 | 3 | 13 | 13 | 0 | 0,80639719 | 0 | Peripherals |
| OTU0505 | 3 | 12 | 12 | 0 | 0,61829992 | 0 | Peripherals |
| OTU0387 | 3 | 14 | 14 | 0 | 0,99449446 | 0 | Peripherals |
| OTU0461 | 3 | 15 | 14 | 1 | 0,99449446 | 0,12444444 | Peripherals |
| OTU0167 | 3 | 15 | 11 | 4 | 0,43020266 | 0,42666667 | Peripherals |
| OTU0809 | 3 | 17 | 17 | 0 | 1,55878625 | 0 | Peripherals |
| OTU0740 | 6 | 17 | 17 | 0 | 1,33099777 | 0 | Peripherals |
| OTU0400 | 6 | 13 | 12 | 1 | 0,43539886 | 0,14201183 | Peripherals |
| OTU0245 | 6 | 16 | 16 | 0 | 1,15187799 | 0 | Peripherals |
| OTU0375 | 6 | 14 | 14 | 0 | 0,79363842 | 0 | Peripherals |
| OTU0423 | 6 | 17 | 17 | 0 | 1,33099777 | 0 | Peripherals |
| OTU0086 | 1 | 4 | 4 | 0 | 0,42640143 | 0 | Peripherals |
| OTU0148 | 1 | 4 | 3 | 1 | -0,10660036 | 0,375 | Peripherals |
| OTU0299 | 1 | 4 | 2 | 2 | -0,63960215 | 0,5 | Peripherals |
| OTU0671 | 7 | 6 | 3 | 3 | -0,72140842 | 0,5 | Peripherals |
| OTU0366 | 1 | 4 | 4 | 0 | 0,42640143 | 0 | Peripherals |
| OTU0859 | 3 | 15 | 12 | 3 | 0,61829992 | 0,32 | Peripherals |
| OTU0528 | 3 | 7 | 6 | 1 | -0,51028367 | 0,24489796 | Peripherals |
| OTU0895 | 3 | 12 | 12 | 0 | 0,61829992 | 0 | Peripherals |
| OTU0007 | 3 | 12 | 12 | 0 | 0,61829992 | 0 | Peripherals |
| OTU0642 | 3 | 13 | 10 | 3 | 0,24210539 | 0,35502959 | Peripherals |
| OTU0258 | 3 | 21 | 11 | 10 | 0,43020266 | 0,49886621 | Peripherals |
| OTU0825 | 6 | 7 | 5 | 2 | -0,81843962 | 0,40816327 | Peripherals |
| OTU0512 | 5 | 28 | 26 | 2 | 0,80765343 | 0,13265306 | Peripherals |
| OTU0543 | 5 | 21 | 20 | 1 | 0,16863094 | 0,09070295 | Peripherals |
| OTU0644 | 3 | 24 | 9 | 15 | 0,05400813 | 0,46875 | Peripherals |

| | | | | | | | |
|---|---|---|---|---|---|---|---|
| OTU0359 | 5 | 37 | 31 | 6 | 1,34017217 | 0,27903579 | Peripherals |
| OTU0399 | 5 | 32 | 29 | 3 | 1,12716467 | 0,16992188 | Peripherals |
| OTU0137 | 5 | 39 | 29 | 10 | 1,12716467 | 0,39316239 | Peripherals |
| OTU0168 | 3 | 2 | 1 | 1 | -1,45077 | 0,5 | Peripherals |
| OTU0540 | 5 | 27 | 25 | 2 | 0,70114968 | 0,1399177 | Peripherals |
| OTU0618 | 5 | 15 | 14 | 1 | -0,47039156 | 0,12444444 | Peripherals |
| OTU0522 | 10 | 11 | 11 | 0 | 0,50108484 | 0 | Peripherals |
| OTU0575 | 10 | 11 | 11 | 0 | 0,50108484 | 0 | Peripherals |
| OTU0577 | 5 | 29 | 25 | 4 | 0,70114968 | 0,24494649 | Peripherals |
| OTU0630 | 10 | 2 | 2 | 0 | -2,15171723 | 0 | Peripherals |
| OTU0635 | 7 | 2 | 1 | 1 | -1,63956459 | 0,5 | Peripherals |
| OTU0365 | 10 | 9 | 5 | 4 | -1,26744988 | 0,56790123 | Peripherals |
| OTU0458 | 10 | 12 | 11 | 1 | 0,50108484 | 0,15277778 | Peripherals |
| OTU0524 | 10 | 10 | 8 | 2 | -0,38318252 | 0,34 | Peripherals |
| OTU0092 | 6 | 8 | 6 | 2 | -0,63931984 | 0,375 | Peripherals |
| OTU0605 | 6 | 10 | 10 | 0 | 0,07715929 | 0 | Peripherals |
| OTU0692 | 6 | 10 | 5 | 5 | -0,81843962 | 0,64 | Connector |
| OTU0073 | 6 | 14 | 14 | 0 | 0,79363842 | 0 | Peripherals |
| OTU0074 | 6 | 10 | 9 | 1 | -0,10196049 | 0,18 | Peripherals |
| OTU0183 | 6 | 14 | 14 | 0 | 0,79363842 | 0 | Peripherals |
| OTU0197 | 6 | 10 | 9 | 1 | -0,10196049 | 0,18 | Peripherals |
| OTU0889 | 6 | 19 | 17 | 2 | 1,33099777 | 0,19390582 | Peripherals |
| OTU0002 | 6 | 11 | 11 | 0 | 0,25627907 | 0 | Peripherals |
| OTU0319 | 3 | 17 | 17 | 0 | 1,55878625 | 0 | Peripherals |
| OTU0124 | 3 | 4 | 4 | 0 | -0,8864782 | 0 | Peripherals |
| OTU0169 | 3 | 4 | 3 | 1 | -1,07457547 | 0,375 | Peripherals |
| OTU0175 | 3 | 17 | 17 | 0 | 1,55878625 | 0 | Peripherals |
| OTU0673 | 3 | 22 | 9 | 13 | 0,05400813 | 0,48347107 | Peripherals |
| OTU0435 | 3 | 10 | 10 | 0 | 0,24210539 | 0 | Peripherals |
| OTU0537 | 6 | 6 | 4 | 2 | -0,9975594 | 0,5 | Peripherals |
| OTU0624 | 8 | 5 | 2 | 3 | -0,84893949 | 0,48 | Peripherals |
| OTU0081 | 3 | 8 | 8 | 0 | -0,13408914 | 0 | Peripherals |
| OTU0181 | 3 | 11 | 11 | 0 | 0,43020266 | 0 | Peripherals |
| OTU0371 | 3 | 12 | 12 | 0 | 0,61829992 | 0 | Peripherals |
| OTU0822 | 6 | 9 | 4 | 5 | -0,9975594 | 0,64197531 | Connector |
| OTU0084 | 6 | 8 | 8 | 0 | -0,28108027 | 0 | Peripherals |
| OTU0108 | 6 | 9 | 8 | 1 | -0,28108027 | 0,19753086 | Peripherals |
| OTU0745 | 6 | 9 | 8 | 1 | -0,28108027 | 0,19753086 | Peripherals |
| OTU0142 | 6 | 13 | 13 | 0 | 0,61451864 | 0 | Peripherals |
| OTU0878 | 3 | 18 | 18 | 0 | 1,74688352 | 0 | Peripherals |
| OTU0042 | 3 | 16 | 16 | 0 | 1,37068899 | 0 | Peripherals |
| OTU0407 | 3 | 9 | 9 | 0 | 0,05400813 | 0 | Peripherals |

| | | | | | | | |
|---|---|---|---|---|---|---|---|
| OTU0509 | 3 | 16 | 16 | 0 | 1,37068899 | 0 | Peripherals |
| OTU0203 | 3 | 2 | 2 | 0 | -1,26267274 | 0 | Peripherals |
| OTU0852 | 3 | 8 | 8 | 0 | -0,13408914 | 0 | Peripherals |
| OTU0410 | 5 | 9 | 4 | 5 | -1,53542904 | 0,69135802 | Connector |
| OTU0476 | 5 | 34 | 27 | 7 | 0,91415717 | 0,33737024 | Peripherals |
| OTU0486 | 3 | 17 | 11 | 6 | 0,43020266 | 0,53979239 | Peripherals |
| OTU0198 | 5 | 27 | 24 | 3 | 0,59464593 | 0,20301783 | Peripherals |
| OTU0199 | 3 | 10 | 5 | 5 | -0,69838094 | 0,66 | Connector |
| OTU0542 | 3 | 24 | 17 | 7 | 1,55878625 | 0,41319444 | Peripherals |
| OTU0553 | 5 | 36 | 32 | 4 | 1,44667592 | 0,20216049 | Peripherals |
| OTU0265 | 5 | 32 | 30 | 2 | 1,23366842 | 0,11914063 | Peripherals |
| OTU0279 | 5 | 43 | 33 | 10 | 1,55317967 | 0,3569497 | Peripherals |
| OTU0333 | 3 | 37 | 11 | 26 | 0,43020266 | 0,41782323 | Peripherals |
| OTU0069 | 5 | 20 | 14 | 6 | -0,47039156 | 0,445 | Peripherals |
| OTU0830 | 5 | 34 | 22 | 12 | 0,38163843 | 0,49307958 | Peripherals |
| OTU0516 | 3 | 27 | 15 | 12 | 1,18259172 | 0,52400549 | Peripherals |
| OTU0405 | 5 | 15 | 10 | 5 | -0,89640655 | 0,48 | Peripherals |
| OTU0669 | 3 | 9 | 7 | 2 | -0,32218641 | 0,34567901 | Peripherals |
| OTU0075 | 5 | 20 | 18 | 2 | -0,04437656 | 0,18 | Peripherals |
| OTU0748 | 5 | 2 | 2 | 0 | -1,74843654 | 0 | Peripherals |
| OTU0140 | 5 | 5 | 5 | 0 | -1,42892529 | 0 | Peripherals |
| OTU0184 | 5 | 30 | 24 | 6 | 0,59464593 | 0,32 | Peripherals |
| OTU0462 | 5 | 13 | 9 | 4 | -1,0029103 | 0,4260355 | Peripherals |
| OTU0374 | 10 | 7 | 5 | 2 | -1,26744988 | 0,40816327 | Peripherals |
| OTU0483 | 10 | 11 | 11 | 0 | 0,50108484 | 0 | Peripherals |
| OTU0176 | 10 | 15 | 14 | 1 | 1,38535219 | 0,12444444 | Peripherals |
| OTU0573 | 10 | 12 | 12 | 0 | 0,79584062 | 0 | Peripherals |
| OTU0274 | 10 | 12 | 11 | 1 | 0,50108484 | 0,15277778 | Peripherals |
| OTU0628 | 10 | 11 | 11 | 0 | 0,50108484 | 0 | Peripherals |
| OTU0305 | 10 | 11 | 10 | 1 | 0,20632905 | 0,16528926 | Peripherals |
| OTU0328 | 10 | 7 | 6 | 1 | -0,97269409 | 0,24489796 | Peripherals |
| OTU0043 | 10 | 9 | 8 | 1 | -0,38318252 | 0,19753086 | Peripherals |
| OTU0056 | 10 | 13 | 13 | 0 | 1,09059641 | 0 | Peripherals |
| OTU0892 | 10 | 14 | 14 | 0 | 1,38535219 | 0 | Peripherals |
| OTU0091 | 6 | 12 | 8 | 4 | -0,28108027 | 0,44444444 | Peripherals |
| OTU0714 | 6 | 7 | 4 | 3 | -0,9975594 | 0,57142857 | Peripherals |
| OTU0217 | 3 | 22 | 15 | 7 | 1,18259172 | 0,4338843 | Peripherals |
| OTU0910 | 5 | 33 | 30 | 3 | 1,23366842 | 0,16896235 | Peripherals |
| OTU0912 | 5 | 20 | 19 | 1 | 0,06212719 | 0,095 | Peripherals |
| OTU0736 | 5 | 27 | 19 | 8 | 0,06212719 | 0,46364883 | Peripherals |
| OTU0836 | 3 | 17 | 8 | 9 | -0,13408914 | 0,62283737 | Connector |
| OTU0856 | 5 | 19 | 17 | 2 | -0,15088031 | 0,18836565 | Peripherals |

| | | | | | | | |
|---|---|---|---|---|---|---|---|
| OTU0330 | 5 | 21 | 14 | 7 | -0,47039156 | 0,51247166 | Peripherals |
| OTU0373 | 5 | 20 | 19 | 1 | 0,06212719 | 0,095 | Peripherals |
| OTU0093 | 5 | 15 | 13 | 2 | -0,5768953 | 0,23111111 | Peripherals |
| OTU0416 | 5 | 20 | 15 | 5 | -0,36388781 | 0,395 | Peripherals |
| OTU0182 | 1 | 14 | 8 | 6 | 2,5584086 | 0,6122449 | Module Hub |
| OTU0190 | 5 | 28 | 22 | 6 | 0,38163843 | 0,33673469 | Peripherals |
| OTU0192 | 5 | 33 | 25 | 8 | 0,70114968 | 0,36730946 | Peripherals |
| OTU0187 | 5 | 24 | 16 | 8 | -0,25738406 | 0,46875 | Peripherals |
| OTU0390 | 6 | 15 | 15 | 0 | 0,9727582 | 0 | Peripherals |
| OTU0051 | 6 | 13 | 13 | 0 | 0,61451864 | 0 | Peripherals |
| OTU0712 | 6 | 16 | 16 | 0 | 1,15187799 | 0 | Peripherals |
| OTU0713 | 6 | 12 | 12 | 0 | 0,43539886 | 0 | Peripherals |
| OTU0769 | 6 | 13 | 13 | 0 | 0,61451864 | 0 | Peripherals |
| OTU0119 | 6 | 31 | 31 | 0 | 3,83867473 | 0 | Module Hub |
| OTU0705 | 4 | 7 | 5 | 2 | -0,6986732 | 0,40816327 | Peripherals |
| OTU0411 | 6 | 15 | 15 | 0 | 0,9727582 | 0 | Peripherals |
| OTU0107 | 6 | 14 | 11 | 3 | 0,25627907 | 0,36734694 | Peripherals |
| OTU0174 | 6 | 18 | 18 | 0 | 1,51011755 | 0 | Peripherals |
| OTU0593 | 6 | 19 | 19 | 0 | 1,68923733 | 0 | Peripherals |
| OTU0907 | 6 | 14 | 14 | 0 | 0,79363842 | 0 | Peripherals |
| OTU0446 | 6 | 7 | 6 | 1 | -0,63931984 | 0,24489796 | Peripherals |
| OTU0314 | 8 | 1 | 1 | 0 | -1,34831566 | 0 | Peripherals |
| OTU0621 | 3 | 1 | 1 | 0 | -1,45077 | 0 | Peripherals |
| OTU0315 | 4 | 2 | 2 | 0 | -1,37919905 | 0 | Peripherals |
| OTU0812 | 4 | 2 | 2 | 0 | -1,37919905 | 0 | Peripherals |
| OTU0474 | 3 | 3 | 3 | 0 | -1,07457547 | 0 | Peripherals |
| OTU0138 | 3 | 4 | 4 | 0 | -0,8864782 | 0 | Peripherals |
| OTU0478 | 1 | 1 | 1 | 0 | -1,17260394 | 0 | Peripherals |
| OTU0431 | 6 | 3 | 3 | 0 | -1,17667919 | 0 | Peripherals |
| OTU0536 | 1 | 8 | 6 | 2 | 1,49240501 | 0,40625 | Peripherals |
| OTU0814 | 1 | 2 | 1 | 1 | -1,17260394 | 0,5 | Peripherals |
| OTU0177 | 6 | 9 | 9 | 0 | -0,10196049 | 0 | Peripherals |
| OTU0318 | 6 | 7 | 4 | 3 | -0,9975594 | 0,48979592 | Peripherals |
| OTU0235 | 8 | 3 | 2 | 1 | -0,84893949 | 0,44444444 | Peripherals |
| OTU0264 | 7 | 8 | 6 | 2 | 0,65582584 | 0,375 | Peripherals |
| OTU0466 | 3 | 9 | 9 | 0 | 0,05400813 | 0 | Peripherals |
| OTU0604 | 6 | 9 | 8 | 1 | -0,28108027 | 0,19753086 | Peripherals |
| OTU0900 | 1 | 7 | 6 | 1 | 1,49240501 | 0,24489796 | Peripherals |
| OTU0150 | 3 | 12 | 9 | 3 | 0,05400813 | 0,375 | Peripherals |
| OTU0828 | 3 | 12 | 12 | 0 | 0,61829992 | 0 | Peripherals |
| OTU0922 | 7 | 5 | 4 | 1 | -0,26233033 | 0,32 | Peripherals |
| OTU0607 | 3 | 4 | 4 | 0 | -0,8864782 | 0 | Peripherals |

| | | | | | | | |
|---|---|---|---|---|---|---|---|
| OTU0510 | 3 | 10 | 10 | 0 | 0,24210539 | 0 | Peripherals |
| OTU0429 | 3 | 11 | 11 | 0 | 0,43020266 | 0 | Peripherals |
| OTU0514 | 8 | 4 | 3 | 1 | -0,34956332 | 0,375 | Peripherals |
| OTU0311 | 7 | 6 | 6 | 0 | 0,65582584 | 0 | Peripherals |
| OTU0134 | 3 | 5 | 3 | 2 | -1,07457547 | 0,56 | Peripherals |
| OTU0842 | 8 | 8 | 8 | 0 | 2,14731753 | 0 | Peripherals |
| OTU0806 | 6 | 10 | 9 | 1 | -0,10196049 | 0,18 | Peripherals |
| OTU0006 | 6 | 13 | 13 | 0 | 0,61451864 | 0 | Peripherals |
| OTU0295 | 6 | 8 | 8 | 0 | -0,28108027 | 0 | Peripherals |
| OTU0078 | 3 | 16 | 16 | 0 | 1,37068899 | 0 | Peripherals |
| OTU0398 | 3 | 5 | 4 | 1 | -0,8864782 | 0,32 | Peripherals |
| OTU0670 | 7 | 4 | 3 | 1 | -0,72140842 | 0,375 | Peripherals |
| OTU0396 | 5 | 10 | 5 | 5 | -1,42892529 | 0,66 | Connector |
| OTU0022 | 5 | 33 | 24 | 9 | 0,59464593 | 0,39669421 | Peripherals |
| OTU0455 | 5 | 3 | 2 | 1 | -1,74843654 | 0,44444444 | Peripherals |
| OTU0789 | 1 | 4 | 4 | 0 | 0,42640143 | 0 | Peripherals |
| OTU0336 | 1 | 2 | 2 | 0 | -0,63960215 | 0 | Peripherals |
| OTU0036 | 3 | 17 | 14 | 3 | 0,99449446 | 0,29065744 | Peripherals |
| OTU0790 | 3 | 11 | 10 | 1 | 0,24210539 | 0,16528926 | Peripherals |
| OTU0038 | 5 | 31 | 28 | 3 | 1,02066092 | 0,1748179 | Peripherals |
| OTU0370 | 7 | 7 | 7 | 0 | 1,11490392 | 0 | Peripherals |
| OTU0200 | 3 | 3 | 2 | 1 | -1,26267274 | 0,44444444 | Peripherals |
| OTU0273 | 4 | 18 | 17 | 1 | 2,02343019 | 0,10493827 | Peripherals |
| OTU0296 | 8 | 5 | 5 | 0 | 0,64918902 | 0 | Peripherals |
| OTU0493 | 3 | 14 | 14 | 0 | 0,99449446 | 0 | Peripherals |
| OTU0133 | 3 | 17 | 17 | 0 | 1,55878625 | 0 | Peripherals |
| OTU0369 | 3 | 17 | 16 | 1 | 1,37068899 | 0,11072664 | Peripherals |
| OTU0921 | 5 | 28 | 26 | 2 | 0,80765343 | 0,13265306 | Peripherals |
| OTU0911 | 1 | 3 | 1 | 2 | -1,17260394 | 0,44444444 | Peripherals |
| OTU0145 | 5 | 32 | 25 | 7 | 0,70114968 | 0,34179688 | Peripherals |
| OTU0186 | 3 | 12 | 10 | 2 | 0,24210539 | 0,27777778 | Peripherals |
| OTU0449 | 4 | 13 | 13 | 0 | 1,11606239 | 0 | Peripherals |
| OTU0490 | 3 | 9 | 4 | 5 | -0,8864782 | 0,49382716 | Peripherals |
| OTU0538 | 3 | 29 | 15 | 14 | 1,18259172 | 0,49940547 | Peripherals |
| OTU0937 | 3 | 14 | 14 | 0 | 0,99449446 | 0 | Peripherals |
| OTU0358 | 10 | 11 | 11 | 0 | 0,50108484 | 0 | Peripherals |
| OTU0028 | 5 | 39 | 27 | 12 | 0,91415717 | 0,44049967 | Peripherals |
| OTU0052 | 3 | 8 | 7 | 1 | -0,32218641 | 0,21875 | Peripherals |
| OTU0698 | 3 | 6 | 5 | 1 | -0,69838094 | 0,27777778 | Peripherals |
| OTU0215 | 3 | 24 | 23 | 1 | 2,68736985 | 0,07986111 | Module Hub |
| OTU0652 | 3 | 12 | 7 | 5 | -0,32218641 | 0,48611111 | Peripherals |
| OTU0421 | 1 | 4 | 3 | 1 | -0,10660036 | 0,375 | Peripherals |

| | | | | | | | |
|---|---|---|---|---|---|---|---|
| OTU0744 | 1 | 10 | 7 | 3 | 2,02540681 | 0,48 | Peripherals |
| OTU0906 | 3 | 9 | 6 | 3 | -0,51028367 | 0,44444444 | Peripherals |
| OTU0649 | 5 | 28 | 27 | 1 | 0,91415717 | 0,06887755 | Peripherals |
| OTU0831 | 3 | 8 | 5 | 3 | -0,69838094 | 0,46875 | Peripherals |
| OTU0804 | 6 | 16 | 16 | 0 | 1,15187799 | 0 | Peripherals |
| OTU0647 | 3 | 11 | 11 | 0 | 0,43020266 | 0 | Peripherals |
| OTU0569 | 5 | 25 | 23 | 2 | 0,48814218 | 0,1472 | Peripherals |
| OTU0243 | 6 | 1 | 1 | 0 | -1,53491875 | 0 | Peripherals |

**Table S3.** Overview of the bacterial community composition derived from AD patients that attended synbiotic, prebiotic or placebo baths. All OTUs are summarized up to genus level.

| #OTU ID | Domain | Phylum | Class | Order | Family | Genus |
|---|---|---|---|---|---|---|
| OTU0001 | Bacteria | Nitrospirae | Nitrospira | Nitrospirales | 0319-6A21 | Unclassified |
| OTU0002 | Bacteria | Proteobacteria | Deltaproteobacteria | Oligoflexales | 0319-6G20 | Unclassified |
| OTU0003 | Bacteria | Actinobacteria | Thermoleophilia | Solirubrobacterales | 0319-6M6 | Unclassified |
| OTU0004 | Bacteria | Acidobacteria | Acidobacteria | Subgroup_4 | 11-24 | Unclassified |
| OTU0005 | Bacteria | Actinobacteria | Thermoleophilia | Solirubrobacterales | 288-2 | Unclassified |
| OTU0006 | Bacteria | Bacteroidetes | Bacteroidia | Chitinophagales | 37-13 | Unclassified |
| OTU0007 | Bacteria | Actinobacteria | Thermoleophilia | Solirubrobacterales | 480-2 | Unclassified |
| OTU0008 | Bacteria | Actinobacteria | Thermoleophilia | Solirubrobacterales | 67-14 | Unclassified |
| OTU0009 | Bacteria | Proteobacteria | Alphaproteobacteria | Rhizobiales | A0839 | Unclassified |
| OTU0010 | Bacteria | Proteobacteria | Gammaproteobacteria | Betaproteobacteriales | A21b | Unclassified |
| OTU0011 | Bacteria | Acidobacteria | Holophagae | Subgroup_10 | ABS-19 | Unclassified |
| OTU0012 | Bacteria | Proteobacteria | Alphaproteobacteria | Rhodospirillales | Acetobacteraceae | Acetobacter |
| OTU0013 | Bacteria | Proteobacteria | Alphaproteobacteria | Rhodospirillales | Acetobacteraceae | Acidicaldus |
| OTU0014 | Bacteria | Proteobacteria | Alphaproteobacteria | Rhodospirillales | Acetobacteraceae | Acidiphilium |
| OTU0015 | Bacteria | Proteobacteria | Alphaproteobacteria | Rhodospirillales | Acetobacteraceae | Acidisoma |
| OTU0016 | Bacteria | Proteobacteria | Alphaproteobacteria | Rhodospirillales | Acetobacteraceae | Asaia |
| OTU0017 | Bacteria | Proteobacteria | Alphaproteobacteria | Rhodospirillales | Acetobacteraceae | Belnapia |
| OTU0018 | Bacteria | Proteobacteria | Alphaproteobacteria | Rhodospirillales | Acetobacteraceae | Craurococcus |
| OTU0019 | Bacteria | Proteobacteria | Alphaproteobacteria | Rhodospirillales | Acetobacteraceae | Roseomonas |
| OTU0020 | Bacteria | Proteobacteria | Alphaproteobacteria | Rhodospirillales | Acetobacteraceae | Rubritepida |
| OTU0021 | Bacteria | Proteobacteria | Alphaproteobacteria | Rhodospirillales | Acetobacteraceae | Unclassified |
| OTU0022 | Bacteria | Proteobacteria | Alphaproteobacteria | Rhodospirillales | Acetobacteraceae | uncultured |
| OTU0023 | Bacteria | Proteobacteria | Alphaproteobacteria | Rhodospirillales | Acetobacteraceae | Endobacter |
| OTU0024 | Bacteria | Proteobacteria | Alphaproteobacteria | Rhodospirillales | Acetobacteraceae | Rhodovastum |
| OTU0025 | Bacteria | Firmicutes | Negativicutes | Selenomonadales | Acidaminococcaceae | Acidaminococcus |

| | | | | | | |
|---|---|---|---|---|---|---|
| OTU0026 | Bacteria | Firmicutes | Negativicutes | Selenomonadales | Acidaminococcaceae | Phascolarctobacterium |
| OTU0027 | Bacteria | Firmicutes | Negativicutes | Selenomonadales | Acidaminococcaceae | Succiniclasticum |
| OTU0028 | Bacteria | Actinobacteria | Acidimicrobiia | Acidimicrobiales | Acidimicrobiaceae | CL500-29_marine_group |
| OTU0029 | Bacteria | Actinobacteria | Acidimicrobiia | Acidimicrobiales | Acidimicrobiaceae | Ilumatobacter |
| OTU0030 | Bacteria | Actinobacteria | Acidimicrobiia | Acidimicrobiales | Acidimicrobiaceae | Unclassified |
| OTU0031 | Bacteria | Actinobacteria | Acidimicrobiia | Acidimicrobiales | Acidimicrobiales_Incertae_Sedis | Candidatus_Microthrix |
| OTU0032 | Bacteria | Proteobacteria | Gammaproteobacteria | Acidithiobacillales | Acidithiobacillaceae | KCM-B-112 |
| OTU0033 | Bacteria | Acidobacteria | Acidobacteria | Acidobacteriales | Acidobacteriaceae_(Subgroup_1) | Acidicapsa |
| OTU0034 | Bacteria | Acidobacteria | Acidobacteria | Acidobacteriales | Acidobacteriaceae_(Subgroup_1) | Bryocella |
| OTU0035 | Bacteria | Acidobacteria | Acidobacteria | Acidobacteriales | Acidobacteriaceae_(Subgroup_1) | Edaphobacter |
| OTU0036 | Bacteria | Acidobacteria | Acidobacteria | Acidobacteriales | Acidobacteriaceae_(Subgroup_1) | Granulicella |
| OTU0037 | Bacteria | Acidobacteria | Acidobacteria | Acidobacteriales | Acidobacteriaceae_(Subgroup_1) | Telmatobacter |
| OTU0038 | Bacteria | Acidobacteria | Acidobacteria | Acidobacteriales | Acidobacteriaceae_(Subgroup_1) | Terriglobus |
| OTU0039 | Bacteria | Acidobacteria | Acidobacteria | Acidobacteriales | Acidobacteriaceae_(Subgroup_1) | Unclassified |
| OTU0040 | Bacteria | Acidobacteria | Acidobacteria | Acidobacteriales | Acidobacteriaceae_(Subgroup_1) | Candidatus_Koribacter |
| OTU0041 | Bacteria | Actinobacteria | Actinobacteria | Frankiales | Acidothermaceae | Acidothermus |
| OTU0042 | Bacteria | Actinobacteria | Actinobacteria | Actinomycetales | Actinomycetaceae | Actinobaculum |
| OTU0043 | Bacteria | Actinobacteria | Actinobacteria | Actinomycetales | Actinomycetaceae | Actinomyces |
| OTU0044 | Bacteria | Actinobacteria | Actinobacteria | Actinomycetales | Actinomycetaceae | Arcanobacterium |
| OTU0045 | Bacteria | Actinobacteria | Actinobacteria | Actinomycetales | Actinomycetaceae | Flaviflexus |
| OTU0046 | Bacteria | Actinobacteria | Actinobacteria | Actinomycetales | Actinomycetaceae | Mobiluncus |
| OTU0047 | Bacteria | Actinobacteria | Actinobacteria | Actinomycetales | Actinomycetaceae | Trueperella |
| OTU0048 | Bacteria | Actinobacteria | Actinobacteria | Actinomycetales | Actinomycetaceae | Unclassified |
| OTU0049 | Bacteria | Actinobacteria | Actinobacteria | Actinomycetales | Actinomycetaceae | Varibaculum |
| OTU0050 | Bacteria | Firmicutes | Bacilli | Lactobacillales | Aerococcaceae | Abiotrophia |
| OTU0051 | Bacteria | Firmicutes | Bacilli | Lactobacillales | Aerococcaceae | Aerococcus |
| OTU0052 | Bacteria | Firmicutes | Bacilli | Lactobacillales | Aerococcaceae | Eremococcus |
| OTU0053 | Bacteria | Firmicutes | Bacilli | Lactobacillales | Aerococcaceae | Facklamia |

| | | | | | | |
|---|---|---|---|---|---|---|
| OTU0054 | Bacteria | Firmicutes | Bacilli | Lactobacillales | Aerococcaceae | Unclassified |
| OTU0055 | Bacteria | Firmicutes | Bacilli | Lactobacillales | Aerococcaceae | Ignavigranum |
| OTU0056 | Bacteria | Proteobacteria | Gammaproteobacteria | Aeromonadales | Aeromonadaceae | Aeromonas |
| OTU0057 | Bacteria | Proteobacteria | Alphaproteobacteria | Rickettsiales | AKIW1012 | Unclassified |
| OTU0058 | Bacteria | Chloroflexi | Chloroflexia | Kallotenuales | AKIW781 | Unclassified |
| OTU0059 | Bacteria | Bacteroidetes | Sphingobacteriia | Sphingobacteriales | AKYH767 | Unclassified |
| OTU0060 | Bacteria | Proteobacteria | Betaproteobacteria | Burkholderiales | Alcaligenaceae | Achromobacter |
| OTU0061 | Bacteria | Firmicutes | Bacilli | Bacillales | Alicyclobacillaceae | Tumebacillus |
| OTU0062 | Bacteria | Proteobacteria | Gammaproteobacteria | Alteromonadales | Alteromonadaceae | Alishewanella |
| OTU0063 | Bacteria | Proteobacteria | Gammaproteobacteria | Alteromonadales | Alteromonadaceae | Rheinheimera |
| OTU0064 | Bacteria | Proteobacteria | Gammaproteobacteria | Alteromonadales | Alteromonadaceae | Marinobacter |
| OTU0065 | Bacteria | Chloroflexi | Anaerolineae | Anaerolineales | Anaerolineaceae | Unclassified |
| OTU0066 | Bacteria | Chloroflexi | Anaerolineae | Anaerolineales | Anaerolineaceae | Anaerolinea |
| OTU0067 | Bacteria | Chloroflexi | Anaerolineae | Anaerolineales | Anaerolineaceae | Leptolinea |
| OTU0068 | Bacteria | Chloroflexi | Ardenticatenia | Ardenticatenales | Ardenticatenaceae | uncultured |
| OTU0069 | Bacteria | Proteobacteria | Alphaproteobacteria | Rhizobiales | Aurantimonadaceae | Aureimonas |
| OTU0070 | Bacteria | Proteobacteria | Alphaproteobacteria | Rhizobiales | Aurantimonadaceae | Unclassified |
| OTU0071 | Bacteria | Proteobacteria | Alphaproteobacteria | Azospirillales | Azospirillaceae | Niveispirillum |
| OTU0072 | Bacteria | Firmicutes | Bacilli | Bacillales | Bacillaceae | Aeribacillus |
| OTU0073 | Bacteria | Firmicutes | Bacilli | Bacillales | Bacillaceae | Anoxybacillus |
| OTU0074 | Bacteria | Firmicutes | Bacilli | Bacillales | Bacillaceae | Bacillus |
| OTU0075 | Bacteria | Firmicutes | Bacilli | Bacillales | Bacillaceae | Geobacillus |
| OTU0076 | Bacteria | Firmicutes | Bacilli | Bacillales | Bacillaceae | Lentibacillus |
| OTU0077 | Bacteria | Firmicutes | Bacilli | Bacillales | Bacillaceae | Oceanobacillus |
| OTU0078 | Bacteria | Firmicutes | Bacilli | Bacillales | Bacillaceae | Sinobaca |
| OTU0079 | Bacteria | Firmicutes | Bacilli | Bacillales | Bacillaceae | Unclassified |
| OTU0080 | Bacteria | Firmicutes | Bacilli | Bacillales | Bacillaceae | Ureibacillus |
| OTU0081 | Bacteria | Proteobacteria | Deltaproteobacteria | Bdellovibrionales | Bacteriovoracaceae | Peredibacter |

| | | | | | | |
|---|---|---|---|---|---|---|
| OTU0082 | Bacteria | Bacteroidetes | Bacteroidia | Bacteroidales | Bacteroidaceae | Bacteroides |
| OTU0083 | Bacteria | Bacteroidetes | Bacteroidia | Bacteroidales | Bacteroidales UCG-001 | Unclassified |
| OTU0084 | Bacteria | Proteobacteria | Deltaproteobacteria | Bdellovibrionales | Bdellovibrionaceae | Bdellovibrio |
| OTU0085 | Bacteria | Proteobacteria | Deltaproteobacteria | Bdellovibrionales | Bdellovibrionaceae | OM27_clade |
| OTU0086 | Bacteria | Proteobacteria | Alphaproteobacteria | Rhizobiales | Beijerinckiaceae | 1174-901-12 |
| OTU0087 | Bacteria | Proteobacteria | Alphaproteobacteria | Rhizobiales | Beijerinckiaceae | alphaI cluster |
| OTU0088 | Bacteria | Proteobacteria | Alphaproteobacteria | Rhizobiales | Beijerinckiaceae | Bosea |
| OTU0089 | Bacteria | Proteobacteria | Alphaproteobacteria | Rhizobiales | Beijerinckiaceae | uncultured |
| OTU0090 | Bacteria | Proteobacteria | Alphaproteobacteria | Rhizobiales | Beijerinckiaceae | Chelatococcus |
| OTU0091 | Bacteria | Proteobacteria | Alphaproteobacteria | Rhizobiales | Beijerinckiaceae | Unclassified |
| OTU0092 | Bacteria | Proteobacteria | Alphaproteobacteria | Rhizobiales | Beijerinckiaceae | Methylobacterium |
| OTU0093 | Bacteria | Proteobacteria | Alphaproteobacteria | Rhizobiales | Beijerinckiaceae | Microvirga |
| OTU0094 | Bacteria | Proteobacteria | Alphaproteobacteria | Rhizobiales | Beijerinckiaceae | Rhodoblastus |
| OTU0095 | Bacteria | Proteobacteria | Alphaproteobacteria | Rhizobiales | Beijerinckiaceae | Roseiarcus |
| OTU0096 | Bacteria | Proteobacteria | Alphaproteobacteria | Rhizobiales | Beijerinckiaceae | Methylorosula |
| OTU0097 | Bacteria | Actinobacteria | Actinobacteria | Micrococcales | Beutenbergiaceae | Salana |
| OTU0098 | Bacteria | Actinobacteria | Actinobacteria | Micrococcales | Beutenbergiaceae | Unclassified |
| OTU0099 | Bacteria | Actinobacteria | Actinobacteria | Bifidobacteriales | Bifidobacteriaceae | Bifidobacterium |
| OTU0100 | Bacteria | Actinobacteria | Actinobacteria | Bifidobacteriales | Bifidobacteriaceae | Gardnerella |
| OTU0101 | Bacteria | Actinobacteria | Actinobacteria | Bifidobacteriales | Bifidobacteriaceae | Scardovia |
| OTU0102 | Bacteria | Actinobacteria | Actinobacteria | Bifidobacteriales | Bifidobacteriaceae | Unclassified |
| OTU0103 | Bacteria | Proteobacteria | Deltaproteobacteria | Myxococcales | BIrii41 | Unclassified |
| OTU0104 | Bacteria | Proteobacteria | Deltaproteobacteria | Myxococcales | Blfdi19 | Unclassified |
| OTU0105 | Bacteria | Actinobacteria | Actinobacteria | Micrococcales | Bogoriellaceae | Georgenia |
| OTU0106 | Bacteria | Actinobacteria | Actinobacteria | Micrococcales | Bogoriellaceae | Unclassified |
| OTU0107 | Bacteria | Proteobacteria | Alphaproteobacteria | Rhizobiales | Bradyrhizobiaceae | Unclassified |
| OTU0108 | Bacteria | Proteobacteria | Alphaproteobacteria | Rhizobiales | Bradyrhizobiaceae | Bradyrhizobium |
| OTU0109 | Bacteria | Proteobacteria | Alphaproteobacteria | Rhizobiales | Bradyrhizobiaceae | Rhodopseudomonas |

| | | | | | | |
|---|---|---|---|---|---|---|
| OTU0110 | Bacteria | Proteobacteria | Alphaproteobacteria | Rhizobiales | Bradyrhizobiaceae | Tardiphaga |
| OTU0111 | Bacteria | Actinobacteria | Actinobacteria | Micrococcales | Brevibacteriaceae | Brevibacterium |
| OTU0112 | Bacteria | Proteobacteria | Alphaproteobacteria | Rhizobiales | Brucellaceae | Ochrobactrum |
| OTU0113 | Bacteria | Proteobacteria | Alphaproteobacteria | Rhizobiales | Brucellaceae | Pseudochrobactrum |
| OTU0114 | Bacteria | Proteobacteria | Alphaproteobacteria | Rhizobiales | Brucellaceae | Unclassified |
| OTU0115 | Bacteria | Proteobacteria | Alphaproteobacteria | Rhizobiales | Brucellaceae | Daeguia |
| OTU0116 | Bacteria | Proteobacteria | Gammaproteobacteria | Betaproteobacteriales | Burkholderiaceae | AAP99 |
| OTU0117 | Bacteria | Proteobacteria | Gammaproteobacteria | Betaproteobacteriales | Burkholderiaceae | Advenella |
| OTU0118 | Bacteria | Proteobacteria | Gammaproteobacteria | Betaproteobacteriales | Burkholderiaceae | Alcaligenes |
| OTU0119 | Bacteria | Proteobacteria | Gammaproteobacteria | Betaproteobacteriales | Burkholderiaceae | Aquabacterium |
| OTU0120 | Bacteria | Proteobacteria | Gammaproteobacteria | Betaproteobacteriales | Burkholderiaceae | uncultured |
| OTU0121 | Bacteria | Proteobacteria | Gammaproteobacteria | Betaproteobacteriales | Burkholderiaceae | Azohydromonas |
| OTU0122 | Bacteria | Proteobacteria | Gammaproteobacteria | Betaproteobacteriales | Burkholderiaceae | Unclassified |
| OTU0123 | Bacteria | Proteobacteria | Gammaproteobacteria | Betaproteobacteriales | Burkholderiaceae | Burkholderia-Caballeronia-Paraburkholderia |
| OTU0124 | Bacteria | Proteobacteria | Gammaproteobacteria | Betaproteobacteriales | Burkholderiaceae | Comamonas |
| OTU0125 | Bacteria | Proteobacteria | Gammaproteobacteria | Betaproteobacteriales | Burkholderiaceae | Cupriavidus |
| OTU0126 | Bacteria | Proteobacteria | Gammaproteobacteria | Betaproteobacteriales | Burkholderiaceae | Curvibacter |
| OTU0127 | Bacteria | Proteobacteria | Gammaproteobacteria | Betaproteobacteriales | Burkholderiaceae | Delftia |
| OTU0128 | Bacteria | Proteobacteria | Gammaproteobacteria | Betaproteobacteriales | Burkholderiaceae | Diaphorobacter |
| OTU0129 | Bacteria | Proteobacteria | Gammaproteobacteria | Betaproteobacteriales | Burkholderiaceae | Duganella |
| OTU0130 | Bacteria | Proteobacteria | Gammaproteobacteria | Betaproteobacteriales | Burkholderiaceae | GKS98 freshwater group |
| OTU0131 | Bacteria | Proteobacteria | Gammaproteobacteria | Betaproteobacteriales | Burkholderiaceae | Hydrogenophaga |
| OTU0132 | Bacteria | Proteobacteria | Gammaproteobacteria | Betaproteobacteriales | Burkholderiaceae | Ideonella |
| OTU0133 | Bacteria | Proteobacteria | Gammaproteobacteria | Betaproteobacteriales | Burkholderiaceae | Janthinobacterium |
| OTU0134 | Bacteria | Proteobacteria | Gammaproteobacteria | Betaproteobacteriales | Burkholderiaceae | Lautropia |
| OTU0135 | Bacteria | Proteobacteria | Gammaproteobacteria | Betaproteobacteriales | Burkholderiaceae | Leptothrix |
| OTU0136 | Bacteria | Proteobacteria | Gammaproteobacteria | Betaproteobacteriales | Burkholderiaceae | Limnobacter |

| | | | | | | |
|---|---|---|---|---|---|---|
| OTU0137 | Bacteria | Proteobacteria | Gammaproteobacteria | Betaproteobacteriales | Burkholderiaceae | Massilia |
| OTU0138 | Bacteria | Proteobacteria | Gammaproteobacteria | Betaproteobacteriales | Burkholderiaceae | Noviherbaspirillum |
| OTU0139 | Bacteria | Proteobacteria | Gammaproteobacteria | Betaproteobacteriales | Burkholderiaceae | Oligella |
| OTU0140 | Bacteria | Proteobacteria | Gammaproteobacteria | Betaproteobacteriales | Burkholderiaceae | Ottowia |
| OTU0141 | Bacteria | Proteobacteria | Gammaproteobacteria | Betaproteobacteriales | Burkholderiaceae | Parasutterella |
| OTU0142 | Bacteria | Proteobacteria | Gammaproteobacteria | Betaproteobacteriales | Burkholderiaceae | Pelomonas |
| OTU0143 | Bacteria | Proteobacteria | Gammaproteobacteria | Betaproteobacteriales | Burkholderiaceae | Pigmentiphaga |
| OTU0144 | Bacteria | Proteobacteria | Gammaproteobacteria | Betaproteobacteriales | Burkholderiaceae | Piscinibacter |
| OTU0145 | Bacteria | Proteobacteria | Gammaproteobacteria | Betaproteobacteriales | Burkholderiaceae | Polaromonas |
| OTU0146 | Bacteria | Proteobacteria | Gammaproteobacteria | Betaproteobacteriales | Burkholderiaceae | Pseudacidovorax |
| OTU0147 | Bacteria | Proteobacteria | Gammaproteobacteria | Betaproteobacteriales | Burkholderiaceae | Pseudorhodoferax |
| OTU0148 | Bacteria | Proteobacteria | Gammaproteobacteria | Betaproteobacteriales | Burkholderiaceae | Ralstonia |
| OTU0149 | Bacteria | Proteobacteria | Gammaproteobacteria | Betaproteobacteriales | Burkholderiaceae | Ramlibacter |
| OTU0150 | Bacteria | Proteobacteria | Gammaproteobacteria | Betaproteobacteriales | Burkholderiaceae | Rhizobacter |
| OTU0151 | Bacteria | Proteobacteria | Gammaproteobacteria | Betaproteobacteriales | Burkholderiaceae | Schlegelella |
| OTU0152 | Bacteria | Proteobacteria | Gammaproteobacteria | Betaproteobacteriales | Burkholderiaceae | Sphaerotilus |
| OTU0153 | Bacteria | Proteobacteria | Gammaproteobacteria | Betaproteobacteriales | Burkholderiaceae | Sutterella |
| OTU0154 | Bacteria | Proteobacteria | Gammaproteobacteria | Betaproteobacteriales | Burkholderiaceae | Tepidimonas |
| OTU0155 | Bacteria | Proteobacteria | Gammaproteobacteria | Betaproteobacteriales | Burkholderiaceae | Thiomonas |
| OTU0156 | Bacteria | Proteobacteria | Gammaproteobacteria | Betaproteobacteriales | Burkholderiaceae | Achromobacter |
| OTU0157 | Bacteria | Proteobacteria | Gammaproteobacteria | Betaproteobacteriales | Burkholderiaceae | Verticia |
| OTU0158 | Bacteria | Proteobacteria | Gammaproteobacteria | Betaproteobacteriales | Burkholderiaceae | Bordetella |
| OTU0159 | Bacteria | Proteobacteria | Gammaproteobacteria | Betaproteobacteriales | Burkholderiaceae | Variovorax |
| OTU0160 | Bacteria | Proteobacteria | Gammaproteobacteria | Betaproteobacteriales | Burkholderiaceae | Limnohabitans |
| OTU0161 | Bacteria | Proteobacteria | Gammaproteobacteria | Betaproteobacteriales | Burkholderiaceae | Rhodoferax |
| OTU0162 | Bacteria | Proteobacteria | Gammaproteobacteria | Betaproteobacteriales | Burkholderiaceae | Acidovorax |
| OTU0163 | Bacteria | Proteobacteria | Gammaproteobacteria | Betaproteobacteriales | Burkholderiaceae | Caenimonas |
| OTU0164 | Bacteria | Proteobacteria | Gammaproteobacteria | Betaproteobacteriales | Burkholderiaceae | Xenophilus |

| | | | | | | |
|---|---|---|---|---|---|---|
| OTU0165 | Bacteria | Firmicutes | Clostridia | Clostridiales | Caldicoprobacteraceae | Caldicoprobacter |
| OTU0166 | Bacteria | Chloroflexi | Caldilineae | Caldilineales | Caldilineaceae | Unclassified |
| OTU0167 | Bacteria | Proteobacteria | Epsilonproteobacteria | Campylobacterales | Campylobacteraceae | Arcobacter |
| OTU0168 | Bacteria | Proteobacteria | Epsilonproteobacteria | Campylobacterales | Campylobacteraceae | Campylobacter |
| OTU0169 | Bacteria | Proteobacteria | Gammaproteobacteria | Cardiobacteriales | Cardiobacteriaceae | Cardiobacterium |
| OTU0170 | Bacteria | Proteobacteria | Gammaproteobacteria | Cardiobacteriales | Cardiobacteriaceae | Unclassified |
| OTU0171 | Bacteria | Firmicutes | Bacilli | Lactobacillales | Carnobacteriaceae | Alkalibacterium |
| OTU0172 | Bacteria | Firmicutes | Bacilli | Lactobacillales | Carnobacteriaceae | Alloiococcus |
| OTU0173 | Bacteria | Firmicutes | Bacilli | Lactobacillales | Carnobacteriaceae | Atopostipes |
| OTU0174 | Bacteria | Firmicutes | Bacilli | Lactobacillales | Carnobacteriaceae | Carnobacterium |
| OTU0175 | Bacteria | Firmicutes | Bacilli | Lactobacillales | Carnobacteriaceae | Dolosigranulum |
| OTU0176 | Bacteria | Firmicutes | Bacilli | Lactobacillales | Carnobacteriaceae | Granulicatella |
| OTU0177 | Bacteria | Firmicutes | Bacilli | Lactobacillales | Carnobacteriaceae | Trichococcus |
| OTU0178 | Bacteria | Firmicutes | Bacilli | Lactobacillales | Carnobacteriaceae | Unclassified |
| OTU0179 | Bacteria | Actinobacteria | Actinobacteria | Catenulisporales | Catenulisporaceae | Catenulispora |
| OTU0180 | Bacteria | Proteobacteria | Alphaproteobacteria | Caulobacterales | Caulobacteraceae | Asticcacaulis |
| OTU0181 | Bacteria | Proteobacteria | Alphaproteobacteria | Caulobacterales | Caulobacteraceae | Brevundimonas |
| OTU0182 | Bacteria | Proteobacteria | Alphaproteobacteria | Caulobacterales | Caulobacteraceae | Caulobacter |
| OTU0183 | Bacteria | Proteobacteria | Alphaproteobacteria | Caulobacterales | Caulobacteraceae | Phenylobacterium |
| OTU0184 | Bacteria | Proteobacteria | Alphaproteobacteria | Caulobacterales | Caulobacteraceae | Unclassified |
| OTU0185 | Bacteria | Proteobacteria | Alphaproteobacteria | Caulobacterales | Caulobacteraceae | PMMR1 |
| OTU0186 | Bacteria | Actinobacteria | Actinobacteria | Micrococcales | Cellulomonadaceae | Actinotalea |
| OTU0187 | Bacteria | Actinobacteria | Actinobacteria | Micrococcales | Cellulomonadaceae | Cellulomonas |
| OTU0188 | Bacteria | Proteobacteria | Gammaproteobacteria | Cellvibrionales | Cellvibrionaceae | uncultured |
| OTU0189 | Bacteria | Bacteroidetes | Sphingobacteriia | Sphingobacteriales | Chitinophagaceae | Chitinophaga |
| OTU0190 | Bacteria | Bacteroidetes | Sphingobacteriia | Sphingobacteriales | Chitinophagaceae | Ferruginibacter |
| OTU0191 | Bacteria | Bacteroidetes | Sphingobacteriia | Sphingobacteriales | Chitinophagaceae | Filimonas |
| OTU0192 | Bacteria | Bacteroidetes | Sphingobacteriia | Sphingobacteriales | Chitinophagaceae | Flavisolibacter |

| | | | | | | |
|---|---|---|---|---|---|---|
| OTU0193 | Bacteria | Bacteroidetes | Sphingobacteriia | Sphingobacteriales | Chitinophagaceae | Flavitalea |
| OTU0194 | Bacteria | Bacteroidetes | Sphingobacteriia | Sphingobacteriales | Chitinophagaceae | Hydrotalea |
| OTU0195 | Bacteria | Bacteroidetes | Sphingobacteriia | Sphingobacteriales | Chitinophagaceae | Niabella |
| OTU0196 | Bacteria | Bacteroidetes | Sphingobacteriia | Sphingobacteriales | Chitinophagaceae | Parasegetibacter |
| OTU0197 | Bacteria | Bacteroidetes | Sphingobacteriia | Sphingobacteriales | Chitinophagaceae | Sediminibacterium |
| OTU0198 | Bacteria | Bacteroidetes | Sphingobacteriia | Sphingobacteriales | Chitinophagaceae | Segetibacter |
| OTU0199 | Bacteria | Bacteroidetes | Sphingobacteriia | Sphingobacteriales | Chitinophagaceae | Taibaiella |
| OTU0200 | Bacteria | Bacteroidetes | Sphingobacteriia | Sphingobacteriales | Chitinophagaceae | Terrimonas |
| OTU0201 | Bacteria | Bacteroidetes | Sphingobacteriia | Sphingobacteriales | Chitinophagaceae | uncultured |
| OTU0202 | Bacteria | Bacteroidetes | Sphingobacteriia | Sphingobacteriales | Chitinophagaceae | Heliimonas |
| OTU0203 | Bacteria | Bacteroidetes | Sphingobacteriia | Sphingobacteriales | Chitinophagaceae | Unclassified |
| OTU0204 | Bacteria | Bacteroidetes | Sphingobacteriia | Sphingobacteriales | Chitinophagaceae | Parafilimonas |
| OTU0205 | Bacteria | Bacteroidetes | Sphingobacteriia | Sphingobacteriales | Chitinophagaceae | Dinghuibacter |
| OTU0206 | Bacteria | Bacteroidetes | Sphingobacteriia | Sphingobacteriales | Chitinophagaceae | Cnuella |
| OTU0207 | Bacteria | Chlamydiae | Chlamydiae | Chlamydiales | Chlamydiales_Incertae_Sedis | Criblamydia |
| OTU0208 | Bacteria | Chloroflexi | Chloroflexia | Chloroflexales | Chloroflexaceae | Chloroflexus |
| OTU0209 | Bacteria | Chloroflexi | Chloroflexia | Chloroflexales | Chloroflexaceae | FFCH7168 |
| OTU0210 | Bacteria | Chloroflexi | Chloroflexia | Chloroflexales | Chloroflexaceae | Chloronema |
| OTU0211 | Bacteria | Firmicutes | Clostridia | Clostridiales | Christensenellaceae | Christensenellaceae R-7 group |
| OTU0212 | Bacteria | Firmicutes | Clostridia | Clostridiales | Christensenellaceae | Unclassified |
| OTU0213 | Bacteria | Proteobacteria | Gammaproteobacteria | Betaproteobacteriales | Chromobacteriaceae | Unclassified |
| OTU0214 | Bacteria | Cyanobacteria | Cyanobacteria | Nostocales | Chroococcidiopsaceae | uncultured |
| OTU0215 | Bacteria | Verrucomicrobia | Spartobacteria | Chthoniobacterales | Chthoniobacteraceae | Chthoniobacter |
| OTU0216 | Bacteria | Verrucomicrobia | Spartobacteria | Chthoniobacterales | Chthoniobacteraceae | LD29 |
| OTU0217 | Bacteria | Verrucomicrobia | Verrucomicrobiae | Chthoniobacterales | Chthoniobacteraceae | Candidatus Udaeobacter |
| OTU0218 | Bacteria | Cloacimonetes | Cloacimonadia | Cloacimonadales | Cloacimonadaceae | W5 |
| OTU0219 | Bacteria | Firmicutes | Clostridia | Clostridiales | Clostridiaceae_1 | Candidatus_Arthromitus |
| OTU0220 | Bacteria | Firmicutes | Clostridia | Clostridiales | Clostridiaceae_1 | Clostridium_sensu_stricto_1 |

| | | | | | | |
|---|---|---|---|---|---|---|
| OTU0221 | Bacteria | Firmicutes | Clostridia | Clostridiales | Clostridiaceae_1 | Clostridium_sensu_stricto_10 |
| OTU0222 | Bacteria | Firmicutes | Clostridia | Clostridiales | Clostridiaceae_1 | Clostridium_sensu_stricto_12 |
| OTU0223 | Bacteria | Firmicutes | Clostridia | Clostridiales | Clostridiaceae_1 | Clostridium_sensu_stricto_13 |
| OTU0224 | Bacteria | Firmicutes | Clostridia | Clostridiales | Clostridiaceae_1 | Clostridium_sensu_stricto_7 |
| OTU0225 | Bacteria | Firmicutes | Clostridia | Clostridiales | Clostridiaceae_1 | Clostridium_sensu_stricto_9 |
| OTU0226 | Bacteria | Firmicutes | Clostridia | Clostridiales | Clostridiaceae_1 | Fonticella |
| OTU0227 | Bacteria | Firmicutes | Clostridia | Clostridiales | Clostridiaceae_1 | Sarcina |
| OTU0228 | Bacteria | Firmicutes | Clostridia | Clostridiales | Clostridiales vadinBB60 group | Unclassified |
| OTU0229 | Bacteria | Firmicutes | Clostridia | Clostridiales | Clostridiales_Incertae_Sedis | Proteiniborus |
| OTU0230 | Bacteria | Cyanobacteria | Oxyphotobacteria | Nostocales | Coleofasciculaceae | Microcoleus PCC-7113 |
| OTU0231 | Bacteria | Proteobacteria | Betaproteobacteria | Burkholderiales | Comamonadaceae | Acidovorax |
| OTU0232 | Bacteria | Proteobacteria | Betaproteobacteria | Burkholderiales | Comamonadaceae | Unclassified |
| OTU0233 | Bacteria | Actinobacteria | Thermoleophilia | Solirubrobacterales | Conexibacteraceae | Conexibacter |
| OTU0234 | Bacteria | Actinobacteria | Coriobacteriia | Coriobacteriales | Coriobacteriaceae | Atopobium |
| OTU0235 | Bacteria | Actinobacteria | Coriobacteriia | Coriobacteriales | Coriobacteriaceae | Olsenella |
| OTU0236 | Bacteria | Actinobacteria | Coriobacteriia | Coriobacteriales | Coriobacteriaceae | Unclassified |
| OTU0237 | Bacteria | Actinobacteria | Coriobacteriia | Coriobacteriales | Coriobacteriaceae | Collinsella |
| OTU0238 | Bacteria | Actinobacteria | Coriobacteriia | Coriobacteriales | Coriobacteriaceae | Gordonibacter |
| OTU0239 | Bacteria | Actinobacteria | Coriobacteriia | Coriobacteriales | Coriobacteriaceae | Phoenicibacter |
| OTU0240 | Bacteria | Actinobacteria | Actinobacteria | Corynebacteriales | Corynebacteriaceae | Corynebacterium |
| OTU0241 | Bacteria | Actinobacteria | Actinobacteria | Corynebacteriales | Corynebacteriaceae | Corynebacterium 1 |
| OTU0242 | Bacteria | Actinobacteria | Actinobacteria | Corynebacteriales | Corynebacteriaceae | Lawsonella |
| OTU0243 | Bacteria | Actinobacteria | Actinobacteria | Corynebacteriales | Corynebacteriaceae | Turicella |
| OTU0244 | Bacteria | Proteobacteria | Gammaproteobacteria | Legionellales | Coxiellaceae | Coxiella |
| OTU0245 | Bacteria | Proteobacteria | Gammaproteobacteria | Legionellales | Coxiellaceae | Aquicella |
| OTU0246 | Bacteria | Proteobacteria | Gammaproteobacteria | Legionellales | Coxiellaceae | Rickettsiella |
| OTU0247 | Bacteria | Bacteroidetes | Flavobacteriia | Flavobacteriales | Cryomorphaceae | Fluviicola |
| OTU0248 | Bacteria | Bacteroidetes | Flavobacteriia | Flavobacteriales | Cryomorphaceae | Owenweeksia |

| | | | | | | |
|---|---|---|---|---|---|---|
| OTU0249 | Bacteria | Actinobacteria | Actinobacteria | Frankiales | Cryptosporangiaceae | Fodinicola |
| OTU0250 | Bacteria | Chlamydiae | Chlamydiae | Chlamydiales | cvE6 | Unclassified |
| OTU0251 | Bacteria | Bacteroidetes | Cytophagia | Cytophagales | Cyclobacteriaceae | uncultured |
| OTU0252 | Bacteria | Proteobacteria | Deltaproteobacteria | Myxococcales | Cystobacteraceae | Anaeromyxobacter |
| OTU0253 | Bacteria | Proteobacteria | Deltaproteobacteria | Myxococcales | Cystobacteraceae | Cystobacter |
| OTU0254 | Bacteria | Proteobacteria | Deltaproteobacteria | Myxococcales | Cystobacteraceae | Unclassified |
| OTU0255 | Bacteria | Proteobacteria | Deltaproteobacteria | Myxococcales | Cystobacteraceae | Hyalangium |
| OTU0256 | Bacteria | Bacteroidetes | Cytophagia | Cytophagales | Cytophagaceae | Rhodocytophaga |
| OTU0257 | Bacteria | Bacteroidetes | Cytophagia | Cytophagales | Cytophagaceae | Sporocytophaga |
| OTU0258 | Bacteria | Bacteroidetes | Cytophagia | Cytophagales | Cytophagaceae | Adhaeribacter |
| OTU0259 | Bacteria | Bacteroidetes | Cytophagia | Cytophagales | Cytophagaceae | Hymenobacter |
| OTU0260 | Bacteria | Bacteroidetes | Cytophagia | Cytophagales | Cytophagaceae | Pontibacter |
| OTU0261 | Bacteria | Bacteroidetes | Cytophagia | Cytophagales | Cytophagaceae | Chryseolinea |
| OTU0262 | Bacteria | Bacteroidetes | Cytophagia | Cytophagales | Cytophagaceae | Ohtaekwangia |
| OTU0263 | Bacteria | Bacteroidetes | Cytophagia | Cytophagales | Cytophagaceae | Siphonobacter |
| OTU0264 | Bacteria | Bacteroidetes | Cytophagia | Cytophagales | Cytophagaceae | Unclassified |
| OTU0265 | Bacteria | Bacteroidetes | Cytophagia | Cytophagales | Cytophagaceae | Dyadobacter |
| OTU0266 | Bacteria | Bacteroidetes | Cytophagia | Cytophagales | Cytophagaceae | Emticicia |
| OTU0267 | Bacteria | Bacteroidetes | Cytophagia | Cytophagales | Cytophagaceae | Fibrella |
| OTU0268 | Bacteria | Bacteroidetes | Cytophagia | Cytophagales | Cytophagaceae | Larkinella |
| OTU0269 | Bacteria | Bacteroidetes | Cytophagia | Cytophagales | Cytophagaceae | Spirosoma |
| OTU0270 | Bacteria | Bacteroidetes | Cytophagia | Cytophagales | Cytophagaceae | Fibrisoma |
| OTU0271 | Bacteria | Proteobacteria | Alphaproteobacteria | Rhodospirillales | DA111 | Unclassified |
| OTU0272 | Bacteria | Firmicutes | Clostridia | Clostridiales | Defluviitaleaceae | Defluviitaleaceae UCG-011 |
| OTU0273 | Bacteria | Firmicutes | Clostridia | Clostridiales | Defluviitaleaceae | Incertae_Sedis |
| OTU0274 | Bacteria | Deinococcus-Thermus | Deinococci | Deinococcales | Deinococcaceae | Deinococcus |
| OTU0275 | Bacteria | Actinobacteria | Actinobacteria | Micrococcales | Dermabacteraceae | Brachybacterium |
| OTU0276 | Bacteria | Actinobacteria | Actinobacteria | Micrococcales | Dermabacteraceae | Dermabacter |

| | | | | | | |
|---|---|---|---|---|---|---|
| OTU0277 | Bacteria | Actinobacteria | Actinobacteria | Micrococcales | Dermacoccaceae | Dermacoccus |
| OTU0278 | Bacteria | Actinobacteria | Actinobacteria | Micrococcales | Dermacoccaceae | Flexivirga |
| OTU0279 | Bacteria | Actinobacteria | Actinobacteria | Micrococcales | Dermacoccaceae | Kytococcus |
| OTU0280 | Bacteria | Actinobacteria | Actinobacteria | Micrococcales | Dermacoccaceae | Unclassified |
| OTU0281 | Bacteria | Actinobacteria | Actinobacteria | Micrococcales | Dermatophilaceae | Mobilicoccus |
| OTU0282 | Bacteria | Actinobacteria | Actinobacteria | Micrococcales | Dermatophilaceae | Unclassified |
| OTU0283 | Bacteria | Proteobacteria | Deltaproteobacteria | Desulfovibrionales | Desulfovibrionaceae | Bilophila |
| OTU0284 | Bacteria | Proteobacteria | Deltaproteobacteria | Desulfovibrionales | Desulfovibrionaceae | Desulfovibrio |
| OTU0285 | Bacteria | Actinobacteria | Actinobacteria | Corynebacteriales | Dietziaceae | Dietzia |
| OTU0286 | Bacteria | Proteobacteria | Gammaproteobacteria | Diplorickettsiales | Diplorickettsiaceae | uncultured |
| OTU0287 | Bacteria | Actinobacteria | Thermoleophilia | Solirubrobacterales | Elev-16S-1332 | Unclassified |
| OTU0288 | Bacteria | Proteobacteria | Alphaproteobacteria | Sphingomonadales | Ellin6055 | Unclassified |
| OTU0289 | Bacteria | Proteobacteria | Gammaproteobacteria | Enterobacteriales | Enterobacteriaceae | Arsenophonus |
| OTU0290 | Bacteria | Proteobacteria | Gammaproteobacteria | Enterobacteriales | Enterobacteriaceae | Buchnera |
| OTU0291 | Bacteria | Proteobacteria | Gammaproteobacteria | Enterobacteriales | Enterobacteriaceae | Candidatus_Hamiltonella |
| OTU0292 | Bacteria | Proteobacteria | Gammaproteobacteria | Enterobacteriales | Enterobacteriaceae | Citrobacter |
| OTU0293 | Bacteria | Proteobacteria | Gammaproteobacteria | Enterobacteriales | Enterobacteriaceae | Cronobacter |
| OTU0294 | Bacteria | Proteobacteria | Gammaproteobacteria | Enterobacteriales | Enterobacteriaceae | Enterobacter |
| OTU0295 | Bacteria | Proteobacteria | Gammaproteobacteria | Enterobacteriales | Enterobacteriaceae | Erwinia |
| OTU0296 | Bacteria | Proteobacteria | Gammaproteobacteria | Enterobacteriales | Enterobacteriaceae | Escherichia-Shigella |
| OTU0297 | Bacteria | Proteobacteria | Gammaproteobacteria | Enterobacteriales | Enterobacteriaceae | Ewingella |
| OTU0298 | Bacteria | Proteobacteria | Gammaproteobacteria | Enterobacteriales | Enterobacteriaceae | Klebsiella |
| OTU0299 | Bacteria | Proteobacteria | Gammaproteobacteria | Enterobacteriales | Enterobacteriaceae | Unclassified |
| OTU0300 | Bacteria | Proteobacteria | Gammaproteobacteria | Enterobacteriales | Enterobacteriaceae | Pantoea |
| OTU0301 | Bacteria | Proteobacteria | Gammaproteobacteria | Enterobacteriales | Enterobacteriaceae | Pectobacterium |
| OTU0302 | Bacteria | Proteobacteria | Gammaproteobacteria | Enterobacteriales | Enterobacteriaceae | Proteus |
| OTU0303 | Bacteria | Proteobacteria | Gammaproteobacteria | Enterobacteriales | Enterobacteriaceae | Providencia |
| OTU0304 | Bacteria | Proteobacteria | Gammaproteobacteria | Enterobacteriales | Enterobacteriaceae | Rahnella |

| | | | | | | |
|---|---|---|---|---|---|---|
| OTU0305 | Bacteria | Proteobacteria | Gammaproteobacteria | Enterobacteriales | Enterobacteriaceae | Serratia |
| OTU0306 | Bacteria | Proteobacteria | Gammaproteobacteria | Enterobacteriales | Enterobacteriaceae | Tatumella |
| OTU0307 | Bacteria | Proteobacteria | Gammaproteobacteria | Enterobacteriales | Enterobacteriaceae | Raoultella |
| OTU0308 | Bacteria | Proteobacteria | Gammaproteobacteria | Enterobacteriales | Enterobacteriaceae | Hafnia-Obesumbacterium |
| OTU0309 | Bacteria | Proteobacteria | Gammaproteobacteria | Enterobacteriales | Enterobacteriaceae | Leminorella |
| OTU0310 | Bacteria | Proteobacteria | Gammaproteobacteria | Enterobacteriales | Enterobacteriaceae | Trabulsiella |
| OTU0311 | Bacteria | Firmicutes | Bacilli | Lactobacillales | Enterococcaceae | Enterococcus |
| OTU0312 | Bacteria | Firmicutes | Bacilli | Lactobacillales | Enterococcaceae | Vagococcus |
| OTU0313 | Bacteria | Bacteroidetes | Sphingobacteriia | Sphingobacteriales | env.OPS_17 | Unclassified |
| OTU0314 | Bacteria | Firmicutes | Erysipelotrichia | Erysipelotrichales | Erysipelotrichaceae | Allobaculum |
| OTU0315 | Bacteria | Firmicutes | Erysipelotrichia | Erysipelotrichales | Erysipelotrichaceae | Incertae_Sedis |
| OTU0316 | Bacteria | Firmicutes | Erysipelotrichia | Erysipelotrichales | Erysipelotrichaceae | Solobacterium |
| OTU0317 | Bacteria | Firmicutes | Erysipelotrichia | Erysipelotrichales | Erysipelotrichaceae | Turicibacter |
| OTU0318 | Bacteria | Firmicutes | Erysipelotrichia | Erysipelotrichales | Erysipelotrichaceae | Erysipelotrichaceae UCG-003 |
| OTU0319 | Bacteria | Proteobacteria | Alphaproteobacteria | Sphingomonadales | Erythrobacteraceae | Altererythrobacter |
| OTU0320 | Bacteria | Proteobacteria | Alphaproteobacteria | Sphingomonadales | Erythrobacteraceae | Unclassified |
| OTU0321 | Bacteria | Actinobacteria | Nitriliruptoria | Euzebyales | Euzebyaceae | Euzebya |
| OTU0322 | Bacteria | Proteobacteria | Alphaproteobacteria | Rhizobiales | F0723 | Unclassified |
| OTU0323 | Bacteria | Firmicutes | Clostridia | Clostridiales | Family XI | W5053 |
| OTU0324 | Bacteria | Firmicutes | Clostridia | Clostridiales | Family XIII | Mogibacterium |
| OTU0325 | Bacteria | Firmicutes | Clostridia | Thermoanaerobacterales | Family_III | Caldicellulosiruptor |
| OTU0326 | Bacteria | Firmicutes | Clostridia | Thermoanaerobacterales | Family_III | Thermoanaerobacterium |
| OTU0327 | Bacteria | Firmicutes | Bacilli | Bacillales | Family_X | Thermicanus |
| OTU0328 | Bacteria | Firmicutes | Bacilli | Bacillales | Family_XI | Gemella |
| OTU0329 | Bacteria | Firmicutes | Clostridia | Clostridiales | Family_XI | Anaerococcus |
| OTU0330 | Bacteria | Firmicutes | Clostridia | Clostridiales | Family_XI | Finegoldia |
| OTU0331 | Bacteria | Firmicutes | Clostridia | Clostridiales | Family_XI | Gallicola |
| OTU0332 | Bacteria | Firmicutes | Clostridia | Clostridiales | Family_XI | Helcococcus |

| | | | | | | |
|---|---|---|---|---|---|---|
| OTU0333 | Bacteria | Firmicutes | Clostridia | Clostridiales | Family_XI | Murdochiella |
| OTU0334 | Bacteria | Firmicutes | Clostridia | Clostridiales | Family_XI | Parvimonas |
| OTU0335 | Bacteria | Firmicutes | Clostridia | Clostridiales | Family_XI | Peptoniphilus |
| OTU0336 | Bacteria | Firmicutes | Clostridia | Clostridiales | Family_XI | Tepidimicrobium |
| OTU0337 | Bacteria | Firmicutes | Clostridia | Clostridiales | Family_XI | Tissierella |
| OTU0338 | Bacteria | Firmicutes | Clostridia | Clostridiales | Family_XI | W5053 |
| OTU0339 | Bacteria | Firmicutes | Clostridia | Clostridiales | Family_XI | Unclassified |
| OTU0340 | Bacteria | Firmicutes | Bacilli | Bacillales | Family_XII | Exiguobacterium |
| OTU0341 | Bacteria | Firmicutes | Clostridia | Clostridiales | Family_XII | Fusibacter |
| OTU0342 | Bacteria | Firmicutes | Clostridia | Clostridiales | Family_XIII | Incertae_Sedis |
| OTU0343 | Bacteria | Firmicutes | Clostridia | Clostridiales | Family_XIII | Mogibacterium |
| OTU0344 | Bacteria | Firmicutes | Clostridia | Clostridiales | Family_XIII | Family XIII AD3011 group |
| OTU0345 | Bacteria | Firmicutes | Clostridia | Clostridiales | Family_XVIII | Unclassified |
| OTU0346 | Bacteria | Cyanobacteria | Cyanobacteria | SubsectionIV | FamilyI | Aphanizomenon |
| OTU0347 | Bacteria | Cyanobacteria | Cyanobacteria | SubsectionIII | FamilyI | Phormidium |
| OTU0348 | Bacteria | Cyanobacteria | Cyanobacteria | SubsectionIII | FamilyI | Leptolyngbya |
| OTU0349 | Bacteria | Cyanobacteria | Cyanobacteria | SubsectionI | FamilyI | Gloeocalita |
| OTU0350 | Bacteria | Cyanobacteria | Cyanobacteria | SubsectionIV | FamilyI | Nostoc |
| OTU0351 | Bacteria | Cyanobacteria | Cyanobacteria | SubsectionIII | FamilyI | Microcoleus |
| OTU0352 | Bacteria | Cyanobacteria | Cyanobacteria | SubsectionII | FamilyII | Chroococcidiopsis |
| OTU0353 | Bacteria | Cyanobacteria | Cyanobacteria | SubsectionIV | FamilyII | Calothrix |
| OTU0354 | Bacteria | Cyanobacteria | Cyanobacteria | SubsectionII | FamilyII | Pleurocapsa |
| OTU0355 | Bacteria | Armatimonadetes | Fimbriimonadia | Fimbriimonadales | Fimbriimonadaceae | Unclassified |
| OTU0356 | Bacteria | Bacteroidetes | Cytophagia | Cytophagales | Flammeovirgaceae | Marivirga |
| OTU0357 | Bacteria | Bacteroidetes | Cytophagia | Cytophagales | Flammeovirgaceae | Porifericola |
| OTU0358 | Bacteria | Bacteroidetes | Flavobacteriia | Flavobacteriales | Flavobacteriaceae | Capnocytophaga |
| OTU0359 | Bacteria | Bacteroidetes | Flavobacteriia | Flavobacteriales | Flavobacteriaceae | Flavobacterium |
| OTU0360 | Bacteria | Bacteroidetes | Flavobacteriia | Flavobacteriales | Flavobacteriaceae | Gillisia |

| | | | | | | |
|---|---|---|---|---|---|---|
| OTU0361 | Bacteria | Bacteroidetes | Flavobacteriia | Flavobacteriales | Flavobacteriaceae | Myroides |
| OTU0362 | Bacteria | Bacteroidetes | Flavobacteriia | Flavobacteriales | Flavobacteriaceae | Pricia |
| OTU0363 | Bacteria | Bacteroidetes | Flavobacteriia | Flavobacteriales | Flavobacteriaceae | Psychroflexus |
| OTU0364 | Bacteria | Bacteroidetes | Flavobacteriia | Flavobacteriales | Flavobacteriaceae | Unclassified |
| OTU0365 | Bacteria | Bacteroidetes | Flavobacteriia | Flavobacteriales | Flavobacteriaceae | Bergeyella |
| OTU0366 | Bacteria | Bacteroidetes | Flavobacteriia | Flavobacteriales | Flavobacteriaceae | Chryseobacterium |
| OTU0367 | Bacteria | Bacteroidetes | Flavobacteriia | Flavobacteriales | Flavobacteriaceae | Cloacibacterium |
| OTU0368 | Bacteria | Bacteroidetes | Flavobacteriia | Flavobacteriales | Flavobacteriaceae | Elizabethkingia |
| OTU0369 | Bacteria | Bacteroidetes | Flavobacteriia | Flavobacteriales | Flavobacteriaceae | Empedobacter |
| OTU0370 | Bacteria | Bacteroidetes | Flavobacteriia | Flavobacteriales | Flavobacteriaceae | Epilithonimonas |
| OTU0371 | Bacteria | Bacteroidetes | Flavobacteriia | Flavobacteriales | Flavobacteriaceae | Wautersiella |
| OTU0372 | Bacteria | Bacteroidetes | Flavobacteriia | Flavobacteriales | Flavobacteriaceae | Weeksella |
| OTU0373 | Bacteria | Actinobacteria | Actinobacteria | Frankiales | Frankiaceae | Jatrophihabitans |
| OTU0374 | Bacteria | Fusobacteria | Fusobacteriia | Fusobacteriales | Fusobacteriaceae | Fusobacterium |
| OTU0375 | Bacteria | Actinobacteria | Thermoleophilia | Gaiellales | Gaiellaceae | Gaiella |
| OTU0376 | Bacteria | Proteobacteria | Gammaproteobacteria | Betaproteobacteriales | Gallionellaceae | Gallionella |
| OTU0377 | Bacteria | Gemmatimonadetes | Gemmatimonadetes | Gemmatimonadales | Gemmatimonadaceae | Gemmatimonas |
| OTU0378 | Bacteria | Gemmatimonadetes | Gemmatimonadetes | Gemmatimonadales | Gemmatimonadaceae | uncultured |
| OTU0379 | Bacteria | Gemmatimonadetes | Gemmatimonadetes | Gemmatimonadales | Gemmatimonadaceae | Gemmatirosa |
| OTU0380 | Bacteria | Proteobacteria | Deltaproteobacteria | Desulfuromonadales | Geobacteraceae | Geobacter |
| OTU0381 | Bacteria | Actinobacteria | Actinobacteria | Frankiales | Geodermatophilaceae | Blastococcus |
| OTU0382 | Bacteria | Actinobacteria | Actinobacteria | Frankiales | Geodermatophilaceae | Geodermatophilus |
| OTU0383 | Bacteria | Actinobacteria | Actinobacteria | Frankiales | Geodermatophilaceae | Modestobacter |
| OTU0384 | Bacteria | Actinobacteria | Actinobacteria | Frankiales | Geodermatophilaceae | Unclassified |
| OTU0385 | Bacteria | Proteobacteria | Deltaproteobacteria | Desulfuromonadales | GR-WP33-58 | Unclassified |
| OTU0386 | Bacteria | Firmicutes | Clostridia | Clostridiales | Gracilibacteraceae | Gracilibacter |
| OTU0387 | Bacteria | Proteobacteria | Deltaproteobacteria | Myxococcales | Haliangiaceae | Haliangium |
| OTU0388 | Bacteria | Proteobacteria | Gammaproteobacteria | Cellvibrionales | Halieaceae | OM60(NOR5) clade |

| | | | | | | |
|---|---|---|---|---|---|---|
| OTU0389 | Bacteria | Proteobacteria | Gammaproteobacteria | Oceanospirillales | Halomonadaceae | Chromohalobacter |
| OTU0390 | Bacteria | Proteobacteria | Gammaproteobacteria | Oceanospirillales | Halomonadaceae | Halomonas |
| OTU0391 | Bacteria | Proteobacteria | Epsilonproteobacteria | Campylobacterales | Helicobacteraceae | Sulfuricurvum |
| OTU0392 | Bacteria | Firmicutes | Clostridia | Clostridiales | Heliobacteriaceae | Hydrogenispora |
| OTU0393 | Bacteria | Chloroflexi | Chloroflexia | Herpetosiphonales | Herpetosiphonaceae | Herpetosiphon |
| OTU0394 | Bacteria | Acidobacteria | Holophagae | Holophagales | Holophagaceae | Unclassified |
| OTU0395 | Bacteria | Proteobacteria | Alphaproteobacteria | Rickettsiales | Holosporaceae | Unclassified |
| OTU0396 | Bacteria | Proteobacteria | Gammaproteobacteria | Betaproteobacteriales | Hydrogenophilaceae | Hydrogenophilus |
| OTU0397 | Bacteria | Proteobacteria | Gammaproteobacteria | Betaproteobacteriales | Hydrogenophilaceae | Tepidiphilus |
| OTU0398 | Bacteria | Proteobacteria | Gammaproteobacteria | Betaproteobacteriales | Hydrogenophilaceae | uncultured |
| OTU0399 | Bacteria | Proteobacteria | Alphaproteobacteria | Rhizobiales | Hyphomicrobiaceae | Devosia |
| OTU0400 | Bacteria | Proteobacteria | Alphaproteobacteria | Rhizobiales | Hyphomicrobiaceae | Hyphomicrobium |
| OTU0401 | Bacteria | Proteobacteria | Alphaproteobacteria | Rhizobiales | Hyphomicrobiaceae | Pedomicrobium |
| OTU0402 | Bacteria | Proteobacteria | Alphaproteobacteria | Rhizobiales | Hyphomicrobiaceae | Unclassified |
| OTU0403 | Bacteria | Proteobacteria | Alphaproteobacteria | Rhizobiales | Hyphomicrobiaceae | Rhodomicrobium |
| OTU0404 | Bacteria | Proteobacteria | Alphaproteobacteria | Rhizobiales | Hyphomicrobiaceae | Prosthecomicrobium |
| OTU0405 | Bacteria | Proteobacteria | Alphaproteobacteria | Rhizobiales | Hyphomicrobiaceae | Rhodoplanes |
| OTU0406 | Bacteria | Proteobacteria | Alphaproteobacteria | Caulobacterales | Hyphomonadaceae | Woodsholea |
| OTU0407 | Bacteria | Actinobacteria | Acidimicrobiia | Acidimicrobiales | Iamiaceae | Iamia |
| OTU0408 | Bacteria | Actinobacteria | Acidimicrobiia | Microtrichales | Ilumatobacteraceae | Unclassified |
| OTU0409 | Bacteria | Actinobacteria | Acidimicrobiia | Microtrichales | Ilumatobacteraceae | uncultured |
| OTU0410 | Bacteria | Actinobacteria | Actinobacteria | Micrococcales | Intrasporangiaceae | Janibacter |
| OTU0411 | Bacteria | Actinobacteria | Actinobacteria | Micrococcales | Intrasporangiaceae | Ornithinibacter |
| OTU0412 | Bacteria | Actinobacteria | Actinobacteria | Micrococcales | Intrasporangiaceae | Ornithinimicrobium |
| OTU0413 | Bacteria | Actinobacteria | Actinobacteria | Micrococcales | Intrasporangiaceae | Phycicoccus |
| OTU0414 | Bacteria | Actinobacteria | Actinobacteria | Micrococcales | Intrasporangiaceae | Terrabacter |
| OTU0415 | Bacteria | Actinobacteria | Actinobacteria | Micrococcales | Intrasporangiaceae | Tetrasphaera |
| OTU0416 | Bacteria | Actinobacteria | Actinobacteria | Micrococcales | Intrasporangiaceae | Unclassified |

| | | | | | | |
|---|---|---|---|---|---|---|
| OTU0417 | Bacteria | Actinobacteria | Actinobacteria | Micrococcales | Intrasporangiaceae | Ornithinicoccus |
| OTU0418 | Bacteria | Actinobacteria | Actinobacteria | Micrococcales | Intrasporangiaceae | Knoellia |
| OTU0419 | Bacteria | Actinobacteria | Actinobacteria | Micrococcales | Intrasporangiaceae | Serinicoccus |
| OTU0420 | Bacteria | Planctomycetes | Planctomycetacia | Isosphaerales | Isosphaeraceae | uncultured |
| OTU0421 | Bacteria | Planctomycetes | Planctomycetacia | Isosphaerales | Isosphaeraceae | Singulisphaera |
| OTU0422 | Bacteria | Planctomycetes | Planctomycetacia | Isosphaerales | Isosphaeraceae | Aquisphaera |
| OTU0423 | Bacteria | Chloroflexi | Chloroflexia | Thermomicrobiales | JG30-KF-CM45 | Unclassified |
| OTU0424 | Bacteria | Proteobacteria | Alphaproteobacteria | Sphingomonadales | JG34-KF-161 | Unclassified |
| OTU0425 | Bacteria | Proteobacteria | Alphaproteobacteria | Rhizobiales | JG34-KF-361 | Unclassified |
| OTU0426 | Bacteria | Proteobacteria | Alphaproteobacteria | Rhodospirillales | JG37-AG-20 | Unclassified |
| OTU0427 | Bacteria | Actinobacteria | Actinobacteria | Kineosporiales | Kineosporiaceae | Kineococcus |
| OTU0428 | Bacteria | Actinobacteria | Actinobacteria | Kineosporiales | Kineosporiaceae | Kineosporia |
| OTU0429 | Bacteria | Actinobacteria | Actinobacteria | Kineosporiales | Kineosporiaceae | Quadrisphaera |
| OTU0430 | Bacteria | Actinobacteria | Actinobacteria | Kineosporiales | Kineosporiaceae | Unclassified |
| OTU0431 | Bacteria | Firmicutes | Clostridia | Clostridiales | Lachnospiraceae | Anaerosporobacter |
| OTU0432 | Bacteria | Firmicutes | Clostridia | Clostridiales | Lachnospiraceae | Anaerostipes |
| OTU0433 | Bacteria | Firmicutes | Clostridia | Clostridiales | Lachnospiraceae | Blautia |
| OTU0434 | Bacteria | Firmicutes | Clostridia | Clostridiales | Lachnospiraceae | Butyrivibrio |
| OTU0435 | Bacteria | Firmicutes | Clostridia | Clostridiales | Lachnospiraceae | Catonella |
| OTU0436 | Bacteria | Firmicutes | Clostridia | Clostridiales | Lachnospiraceae | Cellulosilyticum |
| OTU0437 | Bacteria | Firmicutes | Clostridia | Clostridiales | Lachnospiraceae | Coprococcus |
| OTU0438 | Bacteria | Firmicutes | Clostridia | Clostridiales | Lachnospiraceae | Dorea |
| OTU0439 | Bacteria | Firmicutes | Clostridia | Clostridiales | Lachnospiraceae | Howardella |
| OTU0440 | Bacteria | Firmicutes | Clostridia | Clostridiales | Lachnospiraceae | Incertae_Sedis |
| OTU0441 | Bacteria | Firmicutes | Clostridia | Clostridiales | Lachnospiraceae | Johnsonella |
| OTU0442 | Bacteria | Firmicutes | Clostridia | Clostridiales | Lachnospiraceae | Lachnoanaerobaculum |
| OTU0443 | Bacteria | Firmicutes | Clostridia | Clostridiales | Lachnospiraceae | Lachnospira |
| OTU0444 | Bacteria | Firmicutes | Clostridia | Clostridiales | Lachnospiraceae | Marvinbryantia |

| | | | | | | |
|---|---|---|---|---|---|---|
| OTU0445 | Bacteria | Firmicutes | Clostridia | Clostridiales | Lachnospiraceae | Oribacterium |
| OTU0446 | Bacteria | Firmicutes | Clostridia | Clostridiales | Lachnospiraceae | Pseudobutyrivibrio |
| OTU0447 | Bacteria | Firmicutes | Clostridia | Clostridiales | Lachnospiraceae | Roseburia |
| OTU0448 | Bacteria | Firmicutes | Clostridia | Clostridiales | Lachnospiraceae | Stomatobaculum |
| OTU0449 | Bacteria | Firmicutes | Clostridia | Clostridiales | Lachnospiraceae | [Ruminococcus] torques group |
| OTU0450 | Bacteria | Firmicutes | Clostridia | Clostridiales | Lachnospiraceae | uncultured |
| OTU0451 | Bacteria | Firmicutes | Clostridia | Clostridiales | Lachnospiraceae | Tyzzerella 4 |
| OTU0452 | Bacteria | Firmicutes | Clostridia | Clostridiales | Lachnospiraceae | Unclassified |
| OTU0453 | Bacteria | Firmicutes | Clostridia | Clostridiales | Lachnospiraceae | [Eubacterium] hallii group |
| OTU0454 | Bacteria | Firmicutes | Clostridia | Clostridiales | Lachnospiraceae | CAG-56 |
| OTU0455 | Bacteria | Firmicutes | Bacilli | Lactobacillales | Lactobacillaceae | Lactobacillus |
| OTU0456 | Bacteria | Firmicutes | Bacilli | Lactobacillales | Lactobacillaceae | Pediococcus |
| OTU0457 | Bacteria | Proteobacteria | Gammaproteobacteria | Legionellales | Legionellaceae | Legionella |
| OTU0458 | Bacteria | Fusobacteria | Fusobacteriia | Fusobacteriales | Leptotrichiaceae | Leptotrichia |
| OTU0459 | Bacteria | Fusobacteria | Fusobacteriia | Fusobacteriales | Leptotrichiaceae | Streptobacillus |
| OTU0460 | Bacteria | Fusobacteria | Fusobacteriia | Fusobacteriales | Leptotrichiaceae | Unclassified |
| OTU0461 | Bacteria | Firmicutes | Bacilli | Lactobacillales | Leuconostocaceae | Leuconostoc |
| OTU0462 | Bacteria | Firmicutes | Bacilli | Lactobacillales | Leuconostocaceae | Weissella |
| OTU0463 | Bacteria | Firmicutes | Limnochordia | Limnochordales | Limnochordaceae | Unclassified |
| OTU0464 | Bacteria | Cyanobacteria | Oxyphotobacteria | Limnotrichales | Limnotrichaceae | Limnothrix |
| OTU0465 | Bacteria | Firmicutes | Bacilli | Bacillales | Listeriaceae | Brochothrix |
| OTU0466 | Bacteria | Gemmatimonadetes | Longimicrobia | Longimicrobiales | Longimicrobiaceae | Unclassified |
| OTU0467 | Bacteria | Bacteroidetes | Bacteroidia | Bacteroidales | Marinilabiliaceae | Ruminofilibacter |
| OTU0468 | Bacteria | Proteobacteria | Gammaproteobacteria | Alteromonadales | Marinobacteraceae | Marinobacter |
| OTU0469 | Bacteria | Proteobacteria | Alphaproteobacteria | Rhizobiales | Methylocystaceae | Hansschlegelia |
| OTU0470 | Bacteria | Proteobacteria | Alphaproteobacteria | Rhizobiales | Methylocystaceae | Pleomorphomonas |
| OTU0471 | Bacteria | Proteobacteria | Gammaproteobacteria | Betaproteobacteriales | Methylophilaceae | Methylophilus |
| OTU0472 | Bacteria | Proteobacteria | Gammaproteobacteria | Betaproteobacteriales | Methylophilaceae | Unclassified |

| | | | | | | |
|---|---|---|---|---|---|---|
| OTU0473 | Bacteria | Actinobacteria | Actinobacteria | Micrococcales | Microbacteriaceae | Agrococcus |
| OTU0474 | Bacteria | Actinobacteria | Actinobacteria | Micrococcales | Microbacteriaceae | Agromyces |
| OTU0475 | Bacteria | Actinobacteria | Actinobacteria | Micrococcales | Microbacteriaceae | Amnibacterium |
| OTU0476 | Bacteria | Actinobacteria | Actinobacteria | Micrococcales | Microbacteriaceae | Curtobacterium |
| OTU0477 | Bacteria | Actinobacteria | Actinobacteria | Micrococcales | Microbacteriaceae | Frondihabitans |
| OTU0478 | Bacteria | Actinobacteria | Actinobacteria | Micrococcales | Microbacteriaceae | Herbiconiux |
| OTU0479 | Bacteria | Actinobacteria | Actinobacteria | Micrococcales | Microbacteriaceae | Humibacter |
| OTU0480 | Bacteria | Actinobacteria | Actinobacteria | Micrococcales | Microbacteriaceae | Leucobacter |
| OTU0481 | Bacteria | Actinobacteria | Actinobacteria | Micrococcales | Microbacteriaceae | Lysinimonas |
| OTU0482 | Bacteria | Actinobacteria | Actinobacteria | Micrococcales | Microbacteriaceae | Microbacterium |
| OTU0483 | Bacteria | Actinobacteria | Actinobacteria | Micrococcales | Microbacteriaceae | Pseudoclavibacter |
| OTU0484 | Bacteria | Actinobacteria | Actinobacteria | Micrococcales | Microbacteriaceae | Rathayibacter |
| OTU0485 | Bacteria | Actinobacteria | Actinobacteria | Micrococcales | Microbacteriaceae | Subtercola |
| OTU0486 | Bacteria | Actinobacteria | Actinobacteria | Micrococcales | Microbacteriaceae | Unclassified |
| OTU0487 | Bacteria | Actinobacteria | Actinobacteria | Micrococcales | Microbacteriaceae | Parafrigoribacterium |
| OTU0488 | Bacteria | Actinobacteria | Actinobacteria | Micrococcales | Microbacteriaceae | Marisediminicola |
| OTU0489 | Bacteria | Actinobacteria | Actinobacteria | Micrococcales | Micrococcaceae | Arthrobacter |
| OTU0490 | Bacteria | Actinobacteria | Actinobacteria | Micrococcales | Micrococcaceae | Citricoccus |
| OTU0491 | Bacteria | Actinobacteria | Actinobacteria | Micrococcales | Micrococcaceae | Kocuria |
| OTU0492 | Bacteria | Actinobacteria | Actinobacteria | Micrococcales | Micrococcaceae | Micrococcus |
| OTU0493 | Bacteria | Actinobacteria | Actinobacteria | Micrococcales | Micrococcaceae | Nesterenkonia |
| OTU0494 | Bacteria | Actinobacteria | Actinobacteria | Micrococcales | Micrococcaceae | Rothia |
| OTU0495 | Bacteria | Actinobacteria | Actinobacteria | Micrococcales | Micrococcaceae | Pseudoglutamicibacter |
| OTU0496 | Bacteria | Actinobacteria | Actinobacteria | Micrococcales | Micrococcaceae | Unclassified |
| OTU0497 | Bacteria | Actinobacteria | Actinobacteria | Micrococcales | Micrococcaceae | Tersicoccus |
| OTU0498 | Bacteria | Actinobacteria | Actinobacteria | Micromonosporales | Micromonosporaceae | Plantactinospora |
| OTU0499 | Bacteria | Actinobacteria | Actinobacteria | Micromonosporales | Micromonosporaceae | Actinoplanes |
| OTU0500 | Bacteria | Actinobacteria | Actinobacteria | Micromonosporales | Micromonosporaceae | Luedemannella |

| | | | | | | |
|---|---|---|---|---|---|---|
| OTU0501 | Bacteria | Actinobacteria | Actinobacteria | Micromonosporales | Micromonosporaceae | Micromonospora |
| OTU0502 | Bacteria | Actinobacteria | Actinobacteria | Micromonosporales | Micromonosporaceae | Phytohabitans |
| OTU0503 | Bacteria | Actinobacteria | Actinobacteria | Micromonosporales | Micromonosporaceae | Pilimelia |
| OTU0504 | Bacteria | Actinobacteria | Actinobacteria | Micromonosporales | Micromonosporaceae | Stackebrandtia |
| OTU0505 | Bacteria | Actinobacteria | Actinobacteria | Micromonosporales | Micromonosporaceae | Unclassified |
| OTU0506 | Bacteria | Bacteroidetes | Cytophagia | Cytophagales | Microscillaceae | Siphonobacter |
| OTU0507 | Bacteria | Bacteroidetes | Cytophagia | Cytophagales | Microscillaceae | uncultured |
| OTU0508 | Bacteria | Proteobacteria | Deltaproteobacteria | Myxococcales | mle1-27 | Unclassified |
| OTU0509 | Bacteria | Proteobacteria | Gammaproteobacteria | Pseudomonadales | Moraxellaceae | Acinetobacter |
| OTU0510 | Bacteria | Proteobacteria | Gammaproteobacteria | Pseudomonadales | Moraxellaceae | Alkanindiges |
| OTU0511 | Bacteria | Proteobacteria | Gammaproteobacteria | Pseudomonadales | Moraxellaceae | Enhydrobacter |
| OTU0512 | Bacteria | Proteobacteria | Gammaproteobacteria | Pseudomonadales | Moraxellaceae | Moraxella |
| OTU0513 | Bacteria | Proteobacteria | Gammaproteobacteria | Pseudomonadales | Moraxellaceae | Perlucidibaca |
| OTU0514 | Bacteria | Proteobacteria | Gammaproteobacteria | Pseudomonadales | Moraxellaceae | Psychrobacter |
| OTU0515 | Bacteria | Proteobacteria | Gammaproteobacteria | Pseudomonadales | Moraxellaceae | Unclassified |
| OTU0516 | Bacteria | Actinobacteria | Actinobacteria | Corynebacteriales | Mycobacteriaceae | Mycobacterium |
| OTU0517 | Bacteria | Proteobacteria | Deltaproteobacteria | Myxococcales | Myxococcaceae | Corallococcus |
| OTU0518 | Bacteria | Proteobacteria | Deltaproteobacteria | Myxococcales | Myxococcaceae | Myxococcus |
| OTU0519 | Bacteria | Actinobacteria | Actinobacteria | Frankiales | Nakamurellaceae | Nakamurella |
| OTU0520 | Bacteria | Proteobacteria | Gammaproteobacteria | Betaproteobacteriales | Neisseriaceae | Conchiformibius |
| OTU0521 | Bacteria | Proteobacteria | Gammaproteobacteria | Betaproteobacteriales | Neisseriaceae | Eikenella |
| OTU0522 | Bacteria | Proteobacteria | Gammaproteobacteria | Betaproteobacteriales | Neisseriaceae | Kingella |
| OTU0523 | Bacteria | Proteobacteria | Gammaproteobacteria | Betaproteobacteriales | Neisseriaceae | Unclassified |
| OTU0524 | Bacteria | Proteobacteria | Gammaproteobacteria | Betaproteobacteriales | Neisseriaceae | Neisseria |
| OTU0525 | Bacteria | Proteobacteria | Gammaproteobacteria | Betaproteobacteriales | Neisseriaceae | uncultured |
| OTU0526 | Bacteria | Proteobacteria | Betaproteobacteria | Neisseriales | Neisseriaceae | Unclassified |
| OTU0527 | Bacteria | Proteobacteria | Gammaproteobacteria | Xanthomonadales | Nevskiaceae | Alkanibacter |
| OTU0528 | Bacteria | Proteobacteria | Gammaproteobacteria | Betaproteobacteriales | Nitrosomonadaceae | DSSD61 |

| | | | | | | |
|---|---|---|---|---|---|---|
| OTU0529 | Bacteria | Proteobacteria | Gammaproteobacteria | Betaproteobacteriales | Nitrosomonadaceae | Ellin6067 |
| OTU0530 | Bacteria | Proteobacteria | Gammaproteobacteria | Betaproteobacteriales | Nitrosomonadaceae | IS-44 |
| OTU0531 | Bacteria | Proteobacteria | Gammaproteobacteria | Betaproteobacteriales | Nitrosomonadaceae | MND1 |
| OTU0532 | Bacteria | Proteobacteria | Gammaproteobacteria | Betaproteobacteriales | Nitrosomonadaceae | Nitrosospira |
| OTU0533 | Bacteria | Proteobacteria | Gammaproteobacteria | Betaproteobacteriales | Nitrosomonadaceae | Unclassified |
| OTU0534 | Bacteria | Nitrospirae | Nitrospira | Nitrospirales | Nitrospiraceae | Nitrospira |
| OTU0535 | Bacteria | Actinobacteria | Actinobacteria | Corynebacteriales | Nocardiaceae | Gordonia |
| OTU0536 | Bacteria | Actinobacteria | Actinobacteria | Corynebacteriales | Nocardiaceae | Nocardia |
| OTU0537 | Bacteria | Actinobacteria | Actinobacteria | Corynebacteriales | Nocardiaceae | Rhodococcus |
| OTU0538 | Bacteria | Actinobacteria | Actinobacteria | Corynebacteriales | Nocardiaceae | Williamsia |
| OTU0539 | Bacteria | Actinobacteria | Actinobacteria | Corynebacteriales | Nocardiaceae | Smaragdicoccus |
| OTU0540 | Bacteria | Actinobacteria | Actinobacteria | Propionibacteriales | Nocardioidaceae | Aeromicrobium |
| OTU0541 | Bacteria | Actinobacteria | Actinobacteria | Propionibacteriales | Nocardioidaceae | Kribbella |
| OTU0542 | Bacteria | Actinobacteria | Actinobacteria | Propionibacteriales | Nocardioidaceae | Marmoricola |
| OTU0543 | Bacteria | Actinobacteria | Actinobacteria | Propionibacteriales | Nocardioidaceae | Nocardioides |
| OTU0544 | Bacteria | Actinobacteria | Actinobacteria | Propionibacteriales | Nocardioidaceae | Unclassified |
| OTU0545 | Bacteria | Actinobacteria | Actinobacteria | Streptosporangiales | Nocardiopsaceae | Nocardiopsis |
| OTU0546 | Bacteria | Cyanobacteria | Cyanobacteria | Nostocales | Nostocaceae | Nostoc PCC-7524 |
| OTU0547 | Bacteria | Cyanobacteria | Cyanobacteria | Nostocales | Nostocaceae | Scytonema UTEX 2349 |
| OTU0548 | Bacteria | Cyanobacteria | Oxyphotobacteria | Nostocales | Nostocaceae | Nostoc PCC-7524 |
| OTU0549 | Bacteria | Cyanobacteria | Oxyphotobacteria | Nostocales | Nostocaceae | Scytonema UTEX 2349 |
| OTU0550 | Bacteria | Cyanobacteria | Oxyphotobacteria | Nostocales | Nostocaceae | Unclassified |
| OTU0551 | Bacteria | Bacteroidetes | Flavobacteriia | Flavobacteriales | NS9_marine_group | Unclassified |
| OTU0552 | Bacteria | Proteobacteria | Gammaproteobacteria | Oceanospirillales | Oceanospirillaceae | Marinospirillum |
| OTU0553 | Bacteria | Proteobacteria | Gammaproteobacteria | Oceanospirillales | Oceanospirillaceae | Marinomonas |
| OTU0554 | Bacteria | Proteobacteria | Deltaproteobacteria | Oligoflexales | Oligoflexaceae | Oligoflexus |
| OTU0555 | Bacteria | Actinobacteria | Acidimicrobiia | Acidimicrobiales | OM1_clade | Unclassified |
| OTU0556 | Bacteria | Chlorobi | Chlorobia | Chlorobiales | OPB56 | Unclassified |

| | | | | | | |
|---|---|---|---|---|---|---|
| OTU0557 | Bacteria | Verrucomicrobia | Opitutae | Opitutales | Opitutaceae | Opitutus |
| OTU0558 | Bacteria | Firmicutes | Bacilli | Bacillales | p-251-o5 | Unclassified |
| OTU0559 | Bacteria | Proteobacteria | Deltaproteobacteria | Myxococcales | P3OB-42 | Unclassified |
| OTU0560 | Bacteria | Firmicutes | Bacilli | Bacillales | Paenibacillaceae | Aneurinibacillus |
| OTU0561 | Bacteria | Firmicutes | Bacilli | Bacillales | Paenibacillaceae | Brevibacillus |
| OTU0562 | Bacteria | Firmicutes | Bacilli | Bacillales | Paenibacillaceae | Cohnella |
| OTU0563 | Bacteria | Firmicutes | Bacilli | Bacillales | Paenibacillaceae | Oxalophagus |
| OTU0564 | Bacteria | Firmicutes | Bacilli | Bacillales | Paenibacillaceae | Paenibacillus |
| OTU0565 | Bacteria | Firmicutes | Bacilli | Bacillales | Paenibacillaceae | Saccharibacillus |
| OTU0566 | Bacteria | Firmicutes | Bacilli | Bacillales | Paenibacillaceae | Thermobacillus |
| OTU0567 | Bacteria | Chlamydiae | Chlamydiae | Chlamydiales | Parachlamydiaceae | Candidatus_Protochlamydia |
| OTU0568 | Bacteria | Chlamydiae | Chlamydiae | Chlamydiales | Parachlamydiaceae | Parachlamydia |
| OTU0569 | Bacteria | Chlamydiae | Chlamydiae | Chlamydiales | Parachlamydiaceae | uncultured |
| OTU0570 | Bacteria | Chlamydiae | Chlamydiae | Chlamydiales | Parachlamydiaceae | Unclassified |
| OTU0571 | Bacteria | Chlamydiae | Chlamydiae | Chlamydiales | Parachlamydiaceae | Neochlamydia |
| OTU0572 | Bacteria | Proteobacteria | Gammaproteobacteria | Pasteurellales | Pasteurellaceae | Actinobacillus |
| OTU0573 | Bacteria | Proteobacteria | Gammaproteobacteria | Pasteurellales | Pasteurellaceae | Aggregatibacter |
| OTU0574 | Bacteria | Proteobacteria | Gammaproteobacteria | Pasteurellales | Pasteurellaceae | Bibersteinia |
| OTU0575 | Bacteria | Proteobacteria | Gammaproteobacteria | Pasteurellales | Pasteurellaceae | Haemophilus |
| OTU0576 | Bacteria | Proteobacteria | Gammaproteobacteria | Pasteurellales | Pasteurellaceae | Pasteurella |
| OTU0577 | Bacteria | Proteobacteria | Gammaproteobacteria | Pasteurellales | Pasteurellaceae | uncultured |
| OTU0578 | Bacteria | Proteobacteria | Gammaproteobacteria | Pasteurellales | Pasteurellaceae | Unclassified |
| OTU0579 | Bacteria | Actinobacteria | Thermoleophilia | Solirubrobacterales | Patulibacteraceae | Patulibacter |
| OTU0580 | Bacteria | Verrucomicrobia | Verrucomicrobiae | Pedosphaerales | Pedosphaeraceae | Unclassified |
| OTU0581 | Bacteria | Firmicutes | Clostridia | Clostridiales | Peptococcaceae | Desulfosporosinus |
| OTU0582 | Bacteria | Firmicutes | Clostridia | Clostridiales | Peptococcaceae | Peptococcus |
| OTU0583 | Bacteria | Firmicutes | Clostridia | Clostridiales | Peptostreptococcaceae | Filifactor |
| OTU0584 | Bacteria | Firmicutes | Clostridia | Clostridiales | Peptostreptococcaceae | Incertae_Sedis |

| | | | | | | |
|---|---|---|---|---|---|---|
| OTU0585 | Bacteria | Firmicutes | Clostridia | Clostridiales | Peptostreptococcaceae | Peptostreptococcus |
| OTU0586 | Bacteria | Firmicutes | Clostridia | Clostridiales | Peptostreptococcaceae | Unclassified |
| OTU0587 | Bacteria | Firmicutes | Clostridia | Clostridiales | Peptostreptococcaceae | uncultured |
| OTU0588 | Bacteria | Proteobacteria | Deltaproteobacteria | Myxococcales | Phaselicystidaceae | Phaselicystis |
| OTU0589 | Bacteria | Planctomycetes | Phycisphaerae | Phycisphaerales | Phycisphaeraceae | SM1A02 |
| OTU0590 | Bacteria | Proteobacteria | Alphaproteobacteria | Rhizobiales | Phyllobacteriaceae | Aliihoeflea |
| OTU0591 | Bacteria | Proteobacteria | Alphaproteobacteria | Rhizobiales | Phyllobacteriaceae | Aquamicrobium |
| OTU0592 | Bacteria | Proteobacteria | Alphaproteobacteria | Rhizobiales | Phyllobacteriaceae | Mesorhizobium |
| OTU0593 | Bacteria | Proteobacteria | Alphaproteobacteria | Rhizobiales | Phyllobacteriaceae | Phyllobacterium |
| OTU0594 | Bacteria | Proteobacteria | Alphaproteobacteria | Rhizobiales | Phyllobacteriaceae | Unclassified |
| OTU0595 | Bacteria | Proteobacteria | Alphaproteobacteria | Rhizobiales | Phyllobacteriaceae | Aminobacter |
| OTU0596 | Bacteria | Planctomycetes | Planctomycetacia | Pirellulales | Pirellulaceae | uncultured |
| OTU0597 | Bacteria | Planctomycetes | Planctomycetacia | Planctomycetales | Planctomycetaceae | Gemmata |
| OTU0598 | Bacteria | Planctomycetes | Planctomycetacia | Planctomycetales | Planctomycetaceae | Zavarzinella |
| OTU0599 | Bacteria | Planctomycetes | Planctomycetacia | Planctomycetales | Planctomycetaceae | Isosphaera |
| OTU0600 | Bacteria | Planctomycetes | Planctomycetacia | Planctomycetales | Planctomycetaceae | Singulisphaera |
| OTU0601 | Bacteria | Planctomycetes | Planctomycetacia | Planctomycetales | Planctomycetaceae | Blastopirellula |
| OTU0602 | Bacteria | Planctomycetes | Planctomycetacia | Planctomycetales | Planctomycetaceae | Pir4_lineage |
| OTU0603 | Bacteria | Planctomycetes | Planctomycetacia | Planctomycetales | Planctomycetaceae | Pirellula |
| OTU0604 | Bacteria | Planctomycetes | Planctomycetacia | Planctomycetales | Planctomycetaceae | Planctomyces |
| OTU0605 | Bacteria | Planctomycetes | Planctomycetacia | Planctomycetales | Planctomycetaceae | Schlesneria |
| OTU0606 | Bacteria | Planctomycetes | Planctomycetacia | Planctomycetales | Planctomycetaceae | Unclassified |
| OTU0607 | Bacteria | Firmicutes | Bacilli | Bacillales | Planococcaceae | Lysinibacillus |
| OTU0608 | Bacteria | Firmicutes | Bacilli | Bacillales | Planococcaceae | Paenisporosarcina |
| OTU0609 | Bacteria | Firmicutes | Bacilli | Bacillales | Planococcaceae | Planococcus |
| OTU0610 | Bacteria | Firmicutes | Bacilli | Bacillales | Planococcaceae | Planomicrobium |
| OTU0611 | Bacteria | Firmicutes | Bacilli | Bacillales | Planococcaceae | Rummeliibacillus |
| OTU0612 | Bacteria | Firmicutes | Bacilli | Bacillales | Planococcaceae | Solibacillus |

| | | | | | | |
|---|---|---|---|---|---|---|
| OTU0613 | Bacteria | Firmicutes | Bacilli | Bacillales | Planococcaceae | Sporosarcina |
| OTU0614 | Bacteria | Firmicutes | Bacilli | Bacillales | Planococcaceae | Psychrobacillus |
| OTU0615 | Bacteria | Firmicutes | Bacilli | Bacillales | Planococcaceae | Unclassified |
| OTU0616 | Bacteria | Proteobacteria | Alphaproteobacteria | Rhizobiales | Pleomorphomonadaceae | Chthonobacter |
| OTU0617 | Bacteria | Proteobacteria | Deltaproteobacteria | Myxococcales | Polyangiaceae | Byssovorax |
| OTU0618 | Bacteria | Proteobacteria | Deltaproteobacteria | Myxococcales | Polyangiaceae | Sorangium |
| OTU0619 | Bacteria | Bacteroidetes | Bacteroidia | Bacteroidales | Porphyromonadaceae | Barnesiella |
| OTU0620 | Bacteria | Bacteroidetes | Bacteroidia | Bacteroidales | Porphyromonadaceae | Petrimonas |
| OTU0621 | Bacteria | Bacteroidetes | Bacteroidia | Bacteroidales | Porphyromonadaceae | Proteiniphilum |
| OTU0622 | Bacteria | Bacteroidetes | Bacteroidia | Bacteroidales | Porphyromonadaceae | uncultured |
| OTU0623 | Bacteria | Bacteroidetes | Bacteroidia | Bacteroidales | Porphyromonadaceae | Paludibacter |
| OTU0624 | Bacteria | Bacteroidetes | Bacteroidia | Bacteroidales | Porphyromonadaceae | Porphyromonas |
| OTU0625 | Bacteria | Bacteroidetes | Bacteroidia | Bacteroidales | Porphyromonadaceae | Macellibacteroides |
| OTU0626 | Bacteria | Bacteroidetes | Bacteroidia | Bacteroidales | Porphyromonadaceae | Parabacteroides |
| OTU0627 | Bacteria | Bacteroidetes | Bacteroidia | Bacteroidales | Porphyromonadaceae | Tannerella |
| OTU0628 | Bacteria | Bacteroidetes | Bacteroidia | Bacteroidales | Prevotellaceae | Alloprevotella |
| OTU0629 | Bacteria | Bacteroidetes | Bacteroidia | Bacteroidales | Prevotellaceae | Paraprevotella |
| OTU0630 | Bacteria | Bacteroidetes | Bacteroidia | Bacteroidales | Prevotellaceae | Prevotella |
| OTU0631 | Bacteria | Actinobacteria | Actinobacteria | Micrococcales | Promicromonosporaceae | Cellulosimicrobium |
| OTU0632 | Bacteria | Actinobacteria | Actinobacteria | Micrococcales | Promicromonosporaceae | Unclassified |
| OTU0633 | Bacteria | Actinobacteria | Actinobacteria | Propionibacteriales | Propionibacteriaceae | Friedmanniella |
| OTU0634 | Bacteria | Actinobacteria | Actinobacteria | Propionibacteriales | Propionibacteriaceae | Microlunatus |
| OTU0635 | Bacteria | Actinobacteria | Actinobacteria | Propionibacteriales | Propionibacteriaceae | Propionibacterium |
| OTU0636 | Bacteria | Actinobacteria | Actinobacteria | Propionibacteriales | Propionibacteriaceae | Propionicimonas |
| OTU0637 | Bacteria | Actinobacteria | Actinobacteria | Propionibacteriales | Propionibacteriaceae | Tessaracoccus |
| OTU0638 | Bacteria | Actinobacteria | Actinobacteria | Propionibacteriales | Propionibacteriaceae | uncultured |
| OTU0639 | Bacteria | Proteobacteria | Gammaproteobacteria | Alteromonadales | Pseudoalteromonadaceae | Pseudoalteromonas |
| OTU0640 | Bacteria | Proteobacteria | Gammaproteobacteria | Pseudomonadales | Pseudomonadaceae | Cellvibrio |

| | | | | | | |
|---|---|---|---|---|---|---|
| OTU0641 | Bacteria | Proteobacteria | Gammaproteobacteria | Pseudomonadales | Pseudomonadaceae | Pseudomonas |
| OTU0642 | Bacteria | Actinobacteria | Actinobacteria | Pseudonocardiales | Pseudonocardiaceae | Actinomycetospora |
| OTU0643 | Bacteria | Actinobacteria | Actinobacteria | Pseudonocardiales | Pseudonocardiaceae | Lentzea |
| OTU0644 | Bacteria | Actinobacteria | Actinobacteria | Pseudonocardiales | Pseudonocardiaceae | Pseudonocardia |
| OTU0645 | Bacteria | Actinobacteria | Actinobacteria | Pseudonocardiales | Pseudonocardiaceae | Saccharomonospora |
| OTU0646 | Bacteria | Actinobacteria | Actinobacteria | Pseudonocardiales | Pseudonocardiaceae | Saccharopolyspora |
| OTU0647 | Bacteria | Actinobacteria | Actinobacteria | Pseudonocardiales | Pseudonocardiaceae | Unclassified |
| OTU0648 | Bacteria | Proteobacteria | Gammaproteobacteria | Alteromonadales | Psychromonadaceae | Psychromonas |
| OTU0649 | Bacteria | Acidobacteria | Blastocatellia (Subgroup 4) | Pyrinomonadales | Pyrinomonadaceae | RB41 |
| OTU0650 | Bacteria | Acidobacteria | Acidobacteria | Subgroup_4 | RB41 | Unclassified |
| OTU0651 | Bacteria | Bacteroidetes | Bacteroidia | Bacteroidales | RF16 | Unclassified |
| OTU0652 | Bacteria | Proteobacteria | Alphaproteobacteria | Rhizobiales | Rhizobiaceae | Kaistia |
| OTU0653 | Bacteria | Proteobacteria | Alphaproteobacteria | Rhizobiales | Rhizobiaceae | Allorhizobium-Neorhizobium-Pararhizobium-Rhizobium |
| OTU0654 | Bacteria | Proteobacteria | Alphaproteobacteria | Rhizobiales | Rhizobiaceae | Rhizobium |
| OTU0655 | Bacteria | Proteobacteria | Alphaproteobacteria | Rhizobiales | Rhizobiaceae | Shinella |
| OTU0656 | Bacteria | Proteobacteria | Alphaproteobacteria | Rhizobiales | Rhizobiaceae | Unclassified |
| OTU0657 | Bacteria | Proteobacteria | Alphaproteobacteria | Rhizobiales | Rhizobiaceae | uncultured |
| OTU0658 | Bacteria | Proteobacteria | Alphaproteobacteria | Rhizobiales | Rhizobiales Incertae Sedis | Unclassified |
| OTU0659 | Bacteria | Proteobacteria | Alphaproteobacteria | Rhizobiales | Rhizobiales_Incertae_Sedis | Rhizomicrobium |
| OTU0660 | Bacteria | Proteobacteria | Alphaproteobacteria | Rhizobiales | Rhizobiales_Incertae_Sedis | Nordella |
| OTU0661 | Bacteria | Proteobacteria | Gammaproteobacteria | Xanthomonadales | Rhodanobacteraceae | Unclassified |
| OTU0662 | Bacteria | Proteobacteria | Gammaproteobacteria | Xanthomonadales | Rhodanobacteraceae | uncultured |
| OTU0663 | Bacteria | Proteobacteria | Alphaproteobacteria | Rhodobacterales | Rhodobacteraceae | Amaricoccus |
| OTU0664 | Bacteria | Proteobacteria | Alphaproteobacteria | Rhodobacterales | Rhodobacteraceae | Defluviimonas |
| OTU0665 | Bacteria | Proteobacteria | Alphaproteobacteria | Rhodobacterales | Rhodobacteraceae | Haematobacter |
| OTU0666 | Bacteria | Proteobacteria | Alphaproteobacteria | Rhodobacterales | Rhodobacteraceae | Jannaschia |

| | | | | | | |
|---|---|---|---|---|---|---|
| OTU0667 | Bacteria | Proteobacteria | Alphaproteobacteria | Rhodobacterales | Rhodobacteraceae | Paracoccus |
| OTU0668 | Bacteria | Proteobacteria | Alphaproteobacteria | Rhodobacterales | Rhodobacteraceae | Rhodobacter |
| OTU0669 | Bacteria | Proteobacteria | Alphaproteobacteria | Rhodobacterales | Rhodobacteraceae | Rubellimicrobium |
| OTU0670 | Bacteria | Proteobacteria | Alphaproteobacteria | Rhodobacterales | Rhodobacteraceae | Tabrizicola |
| OTU0671 | Bacteria | Proteobacteria | Alphaproteobacteria | Rhodobacterales | Rhodobacteraceae | Unclassified |
| OTU0672 | Bacteria | Proteobacteria | Alphaproteobacteria | Rhodobacterales | Rhodobacteraceae | Pannonibacter |
| OTU0673 | Bacteria | Proteobacteria | Alphaproteobacteria | Rhizobiales | Rhodobiaceae | Rhodobium |
| OTU0674 | Bacteria | Proteobacteria | Alphaproteobacteria | Rhizobiales | Rhodobiaceae | Parvibaculum |
| OTU0675 | Bacteria | Proteobacteria | Gammaproteobacteria | Betaproteobacteriales | Rhodocyclaceae | Azospira |
| OTU0676 | Bacteria | Proteobacteria | Gammaproteobacteria | Betaproteobacteriales | Rhodocyclaceae | Dechloromonas |
| OTU0677 | Bacteria | Proteobacteria | Gammaproteobacteria | Betaproteobacteriales | Rhodocyclaceae | Methyloversatilis |
| OTU0678 | Bacteria | Proteobacteria | Gammaproteobacteria | Betaproteobacteriales | Rhodocyclaceae | Thauera |
| OTU0679 | Bacteria | Proteobacteria | Gammaproteobacteria | Betaproteobacteriales | Rhodocyclaceae | Uliginosibacterium |
| OTU0680 | Bacteria | Proteobacteria | Gammaproteobacteria | Betaproteobacteriales | Rhodocyclaceae | Unclassified |
| OTU0681 | Bacteria | Proteobacteria | Gammaproteobacteria | Betaproteobacteriales | Rhodocyclaceae | uncultured |
| OTU0682 | Bacteria | Proteobacteria | Gammaproteobacteria | Betaproteobacteriales | Rhodocyclaceae | Zoogloea |
| OTU0683 | Bacteria | Proteobacteria | Alphaproteobacteria | Rhodospirillales | Rhodospirillaceae | Azospirillum |
| OTU0684 | Bacteria | Proteobacteria | Alphaproteobacteria | Rhodospirillales | Rhodospirillaceae | Skermanella |
| OTU0685 | Bacteria | Proteobacteria | Alphaproteobacteria | Rhodospirillales | Rhodospirillaceae | Dongia |
| OTU0686 | Bacteria | Proteobacteria | Alphaproteobacteria | Rhodospirillales | Rhodospirillaceae | Inquilinus |
| OTU0687 | Bacteria | Proteobacteria | Alphaproteobacteria | Rhodospirillales | Rhodospirillaceae | Defluviicoccus |
| OTU0688 | Bacteria | Proteobacteria | Alphaproteobacteria | Rhodospirillales | Rhodospirillaceae | Novispirillum |
| OTU0689 | Bacteria | Proteobacteria | Alphaproteobacteria | Rhodospirillales | Rhodospirillaceae | Thalassospira |
| OTU0690 | Bacteria | Proteobacteria | Alphaproteobacteria | Rhodospirillales | Rhodospirillales_Incertae_Sedis | Candidatus_Alysiosphaera |
| OTU0691 | Bacteria | Proteobacteria | Alphaproteobacteria | Rhodospirillales | Rhodospirillales_Incertae_Sedis | Geminicoccus |
| OTU0692 | Bacteria | Proteobacteria | Alphaproteobacteria | Rhodospirillales | Rhodospirillales_Incertae_Sedis | Reyranella |
| OTU0693 | Bacteria | Bacteroidetes | Cytophagia | Order_II | Rhodothermaceae | Rubrivirga |
| OTU0694 | Bacteria | Proteobacteria | Alphaproteobacteria | Rickettsiales | Rickettsiaceae | Rickettsia |

| | | | | | | |
|---|---|---|---|---|---|---|
| OTU0695 | Bacteria | Proteobacteria | Alphaproteobacteria | Rickettsiales | Rickettsiales_Incertae_Sedis | Candidatus_Odyssella |
| OTU0696 | Bacteria | Proteobacteria | Alphaproteobacteria | Rickettsiales | Rickettsiales_Incertae_Sedis | Unclassified |
| OTU0697 | Bacteria | Bacteroidetes | Bacteroidia | Bacteroidales | Rikenellaceae | Alistipes |
| OTU0698 | Bacteria | Bacteroidetes | Bacteroidia | Bacteroidales | Rikenellaceae | RC9_gut_group |
| OTU0699 | Bacteria | Bacteroidetes | Bacteroidia | Bacteroidales | Rikenellaceae | vadinBC27_wastewater-sludge_group |
| OTU0700 | Bacteria | Chloroflexi | Chloroflexia | Chloroflexales | Roseiflexaceae | Roseiflexus |
| OTU0701 | Bacteria | Verrucomicrobia | Verrucomicrobiae | Verrucomicrobiales | Rubritaleaceae | Luteolibacter |
| OTU0702 | Bacteria | Actinobacteria | Rubrobacteria | Rubrobacterales | Rubrobacteriaceae | Rubrobacter |
| OTU0703 | Bacteria | Firmicutes | Clostridia | Clostridiales | Ruminococcaceae | Acetivibrio |
| OTU0704 | Bacteria | Firmicutes | Clostridia | Clostridiales | Ruminococcaceae | Butyricicoccus |
| OTU0705 | Bacteria | Firmicutes | Clostridia | Clostridiales | Ruminococcaceae | Faecalibacterium |
| OTU0706 | Bacteria | Firmicutes | Clostridia | Clostridiales | Ruminococcaceae | Fastidiosipila |
| OTU0707 | Bacteria | Firmicutes | Clostridia | Clostridiales | Ruminococcaceae | Hydrogenoanaerobacterium |
| OTU0708 | Bacteria | Firmicutes | Clostridia | Clostridiales | Ruminococcaceae | Incertae_Sedis |
| OTU0709 | Bacteria | Firmicutes | Clostridia | Clostridiales | Ruminococcaceae | Oscillospira |
| OTU0710 | Bacteria | Firmicutes | Clostridia | Clostridiales | Ruminococcaceae | Ruminococcaceae UCG-010 |
| OTU0711 | Bacteria | Firmicutes | Clostridia | Clostridiales | Ruminococcaceae | Ruminococcaceae UCG-013 |
| OTU0712 | Bacteria | Firmicutes | Clostridia | Clostridiales | Ruminococcaceae | Ruminococcaceae UCG-014 |
| OTU0713 | Bacteria | Firmicutes | Clostridia | Clostridiales | Ruminococcaceae | Ruminococcus |
| OTU0714 | Bacteria | Firmicutes | Clostridia | Clostridiales | Ruminococcaceae | Subdoligranulum |
| OTU0715 | Bacteria | Firmicutes | Clostridia | Clostridiales | Ruminococcaceae | Ruminiclostridium 1 |
| OTU0716 | Bacteria | Firmicutes | Clostridia | Clostridiales | Ruminococcaceae | Unclassified |
| OTU0717 | Bacteria | Firmicutes | Clostridia | Clostridiales | Ruminococcaceae | [Eubacterium] coprostanoligenes group |
| OTU0718 | Bacteria | Firmicutes | Clostridia | Clostridiales | Ruminococcaceae | Ruminococcaceae UCG-005 |
| OTU0719 | Bacteria | Bacteroidetes | Bacteroidia | Bacteroidales | S24-7 | Unclassified |
| OTU0720 | Bacteria | Patescibacteria | Saccharimonadia | Saccharimonadales | Saccharimonadaceae | Unclassified |

| | | | | | | |
|---|---|---|---|---|---|---|
| OTU0721 | Bacteria | Proteobacteria | Deltaproteobacteria | Myxococcales | Sandaracinaceae | Unclassified |
| OTU0722 | Bacteria | Actinobacteria | Actinobacteria | Micrococcales | Sanguibacteraceae | Sanguibacter |
| OTU0723 | Bacteria | Bacteroidetes | Sphingobacteriia | Sphingobacteriales | Saprospiraceae | Unclassified |
| OTU0724 | Bacteria | Proteobacteria | Gammaproteobacteria | Betaproteobacteriales | SC-I-84 | Unclassified |
| OTU0725 | Bacteria | Proteobacteria | Gammaproteobacteria | Alteromonadales | Shewanellaceae | Shewanella |
| OTU0726 | Bacteria | Chlorobi | Chlorobia | Chlorobiales | SJA-28 | Unclassified |
| OTU0727 | Bacteria | Proteobacteria | Alphaproteobacteria | Rickettsiales | SM2D12 | Unclassified |
| OTU0728 | Bacteria | Acidobacteria | Acidobacteria | Acidobacteriia | Solibacterales | Solibacteraceae (Subgroup 3) |
| OTU0729 | Bacteria | Actinobacteria | Thermoleophilia | Solirubrobacterales | Solirubrobacteraceae | Conexibacter |
| OTU0730 | Bacteria | Actinobacteria | Thermoleophilia | Solirubrobacterales | Solirubrobacteraceae | Solirubrobacter |
| OTU0731 | Bacteria | Actinobacteria | Thermoleophilia | Solirubrobacterales | Solirubrobacteraceae | Unclassified |
| OTU0732 | Bacteria | Bacteroidetes | Sphingobacteriia | Sphingobacteriales | Sphingobacteriaceae | Arcticibacter |
| OTU0733 | Bacteria | Bacteroidetes | Sphingobacteriia | Sphingobacteriales | Sphingobacteriaceae | Mucilaginibacter |
| OTU0734 | Bacteria | Bacteroidetes | Sphingobacteriia | Sphingobacteriales | Sphingobacteriaceae | Nubsella |
| OTU0735 | Bacteria | Bacteroidetes | Sphingobacteriia | Sphingobacteriales | Sphingobacteriaceae | Olivibacter |
| OTU0736 | Bacteria | Bacteroidetes | Sphingobacteriia | Sphingobacteriales | Sphingobacteriaceae | Pedobacter |
| OTU0737 | Bacteria | Bacteroidetes | Sphingobacteriia | Sphingobacteriales | Sphingobacteriaceae | Sphingobacterium |
| OTU0738 | Bacteria | Bacteroidetes | Sphingobacteriia | Sphingobacteriales | Sphingobacteriaceae | Unclassified |
| OTU0739 | Bacteria | Proteobacteria | Alphaproteobacteria | Sphingomonadales | Sphingomonadaceae | Blastomonas |
| OTU0740 | Bacteria | Proteobacteria | Alphaproteobacteria | Sphingomonadales | Sphingomonadaceae | Novosphingobium |
| OTU0741 | Bacteria | Proteobacteria | Alphaproteobacteria | Sphingomonadales | Sphingomonadaceae | Sandarakinorhabdus |
| OTU0742 | Bacteria | Proteobacteria | Alphaproteobacteria | Sphingomonadales | Sphingomonadaceae | Sphingobium |
| OTU0743 | Bacteria | Proteobacteria | Alphaproteobacteria | Sphingomonadales | Sphingomonadaceae | Sphingomonas |
| OTU0744 | Bacteria | Proteobacteria | Alphaproteobacteria | Sphingomonadales | Sphingomonadaceae | Sphingopyxis |
| OTU0745 | Bacteria | Proteobacteria | Alphaproteobacteria | Sphingomonadales | Sphingomonadaceae | Unclassified |
| OTU0746 | Bacteria | Proteobacteria | Alphaproteobacteria | Sphingomonadales | Sphingomonadaceae | Sphingorhabdus |
| OTU0747 | Bacteria | Proteobacteria | Alphaproteobacteria | Sphingomonadales | Sphingomonadaceae | Rhizorhapis |
| OTU0748 | Bacteria | Proteobacteria | Alphaproteobacteria | Sphingomonadales | Sphingomonadaceae | uncultured |

| | | | | | | |
|---|---|---|---|---|---|---|
| OTU0749 | Bacteria | Spirochaetae | Spirochaetes | Spirochaetales | Spirochaetaceae | Treponema |
| OTU0750 | Bacteria | Actinobacteria | Actinobacteria | Frankiales | Sporichthyaceae | Sporichthya |
| OTU0751 | Bacteria | Actinobacteria | Actinobacteria | Frankiales | Sporichthyaceae | uncultured |
| OTU0752 | Bacteria | Firmicutes | Bacilli | Bacillales | Sporolactobacillaceae | Sporolactobacillus |
| OTU0753 | Bacteria | Firmicutes | Bacilli | Bacillales | Sporolactobacillaceae | Tuberibacillus |
| OTU0754 | Bacteria | Firmicutes | Bacilli | Bacillales | Staphylococcaceae | Jeotgalicoccus |
| OTU0755 | Bacteria | Firmicutes | Bacilli | Bacillales | Staphylococcaceae | Macrococcus |
| OTU0756 | Bacteria | Firmicutes | Bacilli | Bacillales | Staphylococcaceae | Nosocomiicoccus |
| OTU0757 | Bacteria | Firmicutes | Bacilli | Bacillales | Staphylococcaceae | Salinicoccus |
| OTU0758 | Bacteria | Firmicutes | Bacilli | Bacillales | Staphylococcaceae | Staphylococcus |
| OTU0759 | Bacteria | Firmicutes | Bacilli | Bacillales | Staphylococcaceae | Unclassified |
| OTU0760 | Bacteria | Proteobacteria | Gammaproteobacteria | Steroidobacterales | Steroidobacteraceae | uncultured |
| OTU0761 | Bacteria | Firmicutes | Bacilli | Lactobacillales | Streptococcaceae | Lactococcus |
| OTU0762 | Bacteria | Firmicutes | Bacilli | Lactobacillales | Streptococcaceae | Streptococcus |
| OTU0763 | Bacteria | Firmicutes | Bacilli | Lactobacillales | Streptococcaceae | Unclassified |
| OTU0764 | Bacteria | Actinobacteria | Actinobacteria | Streptomycetales | Streptomycetaceae | Streptomyces |
| OTU0765 | Bacteria | Actinobacteria | Actinobacteria | Streptomycetales | Streptomycetaceae | Unclassified |
| OTU0766 | Bacteria | Actinobacteria | Actinobacteria | Streptosporangiales | Streptosporangiaceae | Microbispora |
| OTU0767 | Bacteria | Actinobacteria | Actinobacteria | Streptosporangiales | Streptosporangiaceae | Nonomuraea |
| OTU0768 | Bacteria | Actinobacteria | Actinobacteria | Streptosporangiales | Streptosporangiaceae | Streptosporangium |
| OTU0769 | Bacteria | Acidobacteria | Acidobacteria | Acidobacteriia | Subgroup 2 | Unclassified |
| OTU0770 | Bacteria | Actinobacteria | Acidimicrobiia | Acidimicrobiales | Sva0996_marine_group | Unclassified |
| OTU0771 | Bacteria | Synergistetes | Synergistia | Synergistales | Synergistaceae | Anaerobaculum |
| OTU0772 | Bacteria | Synergistetes | Synergistia | Synergistales | Synergistaceae | Cloacibacillus |
| OTU0773 | Bacteria | Synergistetes | Synergistia | Synergistales | Synergistaceae | Fretibacterium |
| OTU0774 | Bacteria | Synergistetes | Synergistia | Synergistales | Synergistaceae | Jonquetella |
| OTU0775 | Bacteria | Synergistetes | Synergistia | Synergistales | Synergistaceae | uncultured |
| OTU0776 | Bacteria | Synergistetes | Synergistia | Synergistales | Synergistaceae | Thermovirga |

| | | | | | | |
|---|---|---|---|---|---|---|
| OTU0777 | Bacteria | Proteobacteria | Deltaproteobacteria | Syntrophobacterales | Syntrophaceae | uncultured |
| OTU0778 | Bacteria | Firmicutes | Clostridia | Clostridiales | Syntrophomonadaceae | Syntrophomonas |
| OTU0779 | Bacteria | Firmicutes | Clostridia | Clostridiales | Syntrophomonadaceae | Unclassified |
| OTU0780 | Bacteria | Planctomycetes | Phycisphaerae | Tepidisphaerales | Tepidisphaeraceae | Unclassified |
| OTU0781 | Bacteria | Verrucomicrobia | Verrucomicrobiae | Chthoniobacterales | Terrimicrobiaceae | Terrimicrobium |
| OTU0782 | Bacteria | Deinococcus-Thermus | Deinococci | Thermales | Thermaceae | Meiothermus |
| OTU0783 | Bacteria | Deinococcus-Thermus | Deinococci | Thermales | Thermaceae | Thermus |
| OTU0784 | Bacteria | Firmicutes | Bacilli | Bacillales | Thermoactinomycetaceae | Laceyella |
| OTU0785 | Bacteria | Firmicutes | Bacilli | Bacillales | Thermoactinomycetaceae | Planifilum |
| OTU0786 | Bacteria | Firmicutes | Bacilli | Bacillales | Thermoactinomycetaceae | Shimazuella |
| OTU0787 | Bacteria | Firmicutes | Bacilli | Bacillales | Thermoactinomycetaceae | Thermoactinomyces |
| OTU0788 | Bacteria | Firmicutes | Bacilli | Bacillales | Thermoactinomycetaceae | Unclassified |
| OTU0789 | Bacteria | Firmicutes | Clostridia | Thermoanaerobacterales | Thermoanaerobacteraceae | Syntrophaceticus |
| OTU0790 | Bacteria | Firmicutes | Clostridia | Thermoanaerobacterales | Thermoanaerobacteraceae | Gelria |
| OTU0791 | Bacteria | Actinobacteria | Actinobacteria | Streptosporangiales | Thermomonosporaceae | Actinomadura |
| OTU0792 | Bacteria | Proteobacteria | Alphaproteobacteria | Rickettsiales | TK34 | Unclassified |
| OTU0793 | Bacteria | Actinobacteria | Thermoleophilia | Solirubrobacterales | TM146 | Unclassified |
| OTU0794 | Bacteria | Proteobacteria | Gammaproteobacteria | Betaproteobacteriales | TRA3-20 | Unclassified |
| OTU0795 | Bacteria | Deinococcus-Thermus | Deinococci | Deinococcales | Trueperaceae | Truepera |
| OTU0796 | Bacteria | Actinobacteria | Actinobacteria | Corynebacteriales | Tsukamurellaceae | Tsukamurella |
| OTU0797 | Bacteria | Proteobacteria | Deltaproteobacteria | GR-WP33-30 | Unclassified | Unclassified |
| OTU0798 | Bacteria | Bacteroidetes | Bacteroidia | Bacteroidales | Unclassified | Unclassified |
| OTU0799 | Bacteria | Patescibacteria | Gracilibacteria | Absconditabacteriales (SR1) | Unclassified | Unclassified |
| OTU0800 | Bacteria | Armatimonadetes | Armatimonadia | Armatimonadales | Unclassified | Unclassified |
| OTU0801 | Bacteria | Firmicutes | Bacilli | Bacillales | Unclassified | Unclassified |
| OTU0802 | Bacteria | Patescibacteria | Parcubacteria | Candidatus Adlerbacteria | Unclassified | Unclassified |
| OTU0803 | Bacteria | Patescibacteria | Parcubacteria | Candidatus Kaiserbacteria | Unclassified | Unclassified |
| OTU0804 | Bacteria | Proteobacteria | Gammaproteobacteria | CCD24 | Unclassified | Unclassified |

| | | | | | | |
|---|---|---|---|---|---|---|
| OTU0805 | Bacteria | Cyanobacteria | Oxyphotobacteria | Chloroplast | Unclassified | Unclassified |
| OTU0806 | Bacteria | Proteobacteria | Gammaproteobacteria | Unclassified | Unclassified | Unclassified |
| OTU0807 | Bacteria | Actinobacteria | Actinobacteria | Frankiales | Unclassified | Unclassified |
| OTU0808 | Bacteria | Cyanobacteria | Melainabacteria | Gastranaerophilales | Unclassified | Unclassified |
| OTU0809 | Bacteria | Actinobacteria | Acidimicrobiia | Acidimicrobiales | Unclassified | Unclassified |
| OTU0810 | Bacteria | Actinobacteria | Acidimicrobiia | IMCC26256 | Unclassified | Unclassified |
| OTU0811 | Bacteria | BD1-5 | Gracilibacteria | JGI 0000069-P22 | Unclassified | Unclassified |
| OTU0812 | Bacteria | Firmicutes | Bacilli | Lactobacillales | Unclassified | Unclassified |
| OTU0813 | Bacteria | Verrucomicrobia | Verrucomicrobiae | LD1-PB3 | Unclassified | Unclassified |
| OTU0814 | Bacteria | Firmicutes | Clostridia | MBA03 | Unclassified | Unclassified |
| OTU0815 | Bacteria | Actinobacteria | Actinobacteria | Micrococcales | Unclassified | Unclassified |
| OTU0816 | Bacteria | Proteobacteria | Deltaproteobacteria | Myxococcales | Unclassified | Unclassified |
| OTU0817 | Bacteria | Cyanobacteria | Melainabacteria | Obscuribacterales | Unclassified | Unclassified |
| OTU0818 | Bacteria | Actinobacteria | Actinobacteria | PeM15 | Unclassified | Unclassified |
| OTU0819 | Bacteria | Proteobacteria | Gammaproteobacteria | PLTA13 | Unclassified | Unclassified |
| OTU0820 | Bacteria | Proteobacteria | Gammaproteobacteria | R7C24 | Unclassified | Unclassified |
| OTU0821 | Bacteria | Proteobacteria | Alphaproteobacteria | Rhizobiales | Unclassified | Unclassified |
| OTU0822 | Bacteria | Patescibacteria | Saccharimonadia | Saccharimonadales | Unclassified | Unclassified |
| OTU0823 | Bacteria | Acidobacteria | Holophagae | Subgroup_7 | Unclassified | Unclassified |
| OTU0824 | Bacteria | Chloroflexi | JG37-AG-4 | Unclassified | Unclassified | Unclassified |
| OTU0825 | Bacteria | Firmicutes | Bacilli | Unclassified | Unclassified | Unclassified |
| OTU0826 | Bacteria | Planctomycetes | BD7-11 | Unclassified | Unclassified | Unclassified |
| OTU0827 | Bacteria | Firmicutes | BRH-c20a | Unclassified | Unclassified | Unclassified |
| OTU0828 | Bacteria | Chloroflexi | Gitt-GS-136 | Unclassified | Unclassified | Unclassified |
| OTU0829 | Bacteria | BD1-5 | Gracilibacteria | Unclassified | Unclassified | Unclassified |
| OTU0830 | Bacteria | Chloroflexi | KD4-96 | Unclassified | Unclassified | Unclassified |
| OTU0831 | Bacteria | Actinobacteria | MB-A2-108 | Unclassified | Unclassified | Unclassified |
| OTU0832 | Bacteria | Patescibacteria | Parcubacteria | Unclassified | Unclassified | Unclassified |

| | | | | | | |
|---|---|---|---|---|---|---|
| OTU0833 | Bacteria | Gemmatimonadetes | Gemmatimonadetes | S0134_terrestrial_group | Unclassified | Unclassified |
| OTU0834 | Bacteria | Cyanobacteria | ML635J-21 | Unclassified | Unclassified | Unclassified |
| OTU0835 | Bacteria | Acidobacteria | Acidobacteria | Subgroup_17 | Unclassified | Unclassified |
| OTU0836 | Bacteria | Acidobacteria | Acidobacteria | Subgroup_6 | Unclassified | Unclassified |
| OTU0837 | Bacteria | Chloroflexi | TK10 | Unclassified | Unclassified | Unclassified |
| OTU0838 | Bacteria | Chloroflexi | Ktedonobacteria | C0119 | Unclassified | Unclassified |
| OTU0839 | Bacteria | Chlamydiae | Chlamydiae | Chlamydiales | Unclassified | Unclassified |
| OTU0840 | Bacteria | Chloroflexi | Chloroflexia | Chloroflexales | Unclassified | Unclassified |
| OTU0841 | Bacteria | Firmicutes | Clostridia | Clostridiales | Unclassified | Unclassified |
| OTU0842 | Bacteria | Actinobacteria | Actinobacteria | Corynebacteriales | Unclassified | Unclassified |
| OTU0843 | Bacteria | Planctomycetes | Phycisphaerae | CPla-3_termite_group | Unclassified | Unclassified |
| OTU0844 | Bacteria | Bacteroidetes | Cytophagia | Cytophagales | Unclassified | Unclassified |
| OTU0845 | Bacteria | Chloroflexi | Thermomicrobia | JG30-KF-CM45 | Unclassified | Unclassified |
| OTU0846 | Bacteria | Proteobacteria | Gammaproteobacteria | KI89A_clade | Unclassified | Unclassified |
| OTU0847 | Bacteria | Tenericutes | Mollicutes | NB1-n | Unclassified | Unclassified |
| OTU0848 | Bacteria | Proteobacteria | Alphaproteobacteria | Rickettsiales | Unclassified | Unclassified |
| OTU0849 | Bacteria | Bacteroidetes | Sphingobacteriia | Sphingobacteriales | Unclassified | Unclassified |
| OTU0850 | Bacteria | Actinobacteria | Actinobacteria | Unclassified | Unclassified | Unclassified |
| OTU0851 | Bacteria | Proteobacteria | Alphaproteobacteria | Unclassified | Unclassified | Unclassified |
| OTU0852 | Bacteria | Unclassified | Unclassified | Unclassified | Unclassified | Unclassified |
| OTU0853 | Bacteria | Bacteroidetes | Cytophagia | Unclassified | Unclassified | Unclassified |
| OTU0854 | Bacteria | Planctomycetes | OM190 | Unclassified | Unclassified | Unclassified |
| OTU0855 | Bacteria | Firmicutes | OPB54 | Unclassified | Unclassified | Unclassified |
| OTU0856 | Bacteria | Armatimonadetes | Unclassified | Unclassified | Unclassified | Unclassified |
| OTU0857 | Bacteria | Bacteroidetes | Unclassified | Unclassified | Unclassified | Unclassified |
| OTU0858 | Bacteria | Candidate_division_BRC1 | Unclassified | Unclassified | Unclassified | Unclassified |
| OTU0859 | Bacteria | Candidate_division_OD1 | Unclassified | Unclassified | Unclassified | Unclassified |
| OTU0860 | Bacteria | Candidate_division_OP11 | Unclassified | Unclassified | Unclassified | Unclassified |

| | | | | | | |
|---|---|---|---|---|---|---|
| OTU0861 | Bacteria | Candidate_division_OP3 | Unclassified | Unclassified | Unclassified | Unclassified |
| OTU0862 | Bacteria | Candidate_division_TM7 | Unclassified | Unclassified | Unclassified | Unclassified |
| OTU0863 | Bacteria | Latescibacteria | Unclassified | Unclassified | Unclassified | Unclassified |
| OTU0864 | Bacteria | Chloroflexi | Unclassified | Unclassified | Unclassified | Unclassified |
| OTU0865 | Bacteria | Cyanobacteria | Unclassified | Unclassified | Unclassified | Unclassified |
| OTU0866 | Bacteria | Firmicutes | Unclassified | Unclassified | Unclassified | Unclassified |
| OTU0867 | Bacteria | Proteobacteria | Unclassified | Unclassified | Unclassified | Unclassified |
| OTU0868 | Bacteria | SHA-109 | Unclassified | Unclassified | Unclassified | Unclassified |
| OTU0869 | Bacteria | FBP | Unclassified | Unclassified | Unclassified | Unclassified |
| OTU0870 | Bacteria | Cyanobacteria | Melainabacteria | Vampirovibrionales | Unclassified | Unclassified |
| OTU0871 | Bacteria | Planctomycetes | Phycisphaerae | WD2101_soil_group | Unclassified | Unclassified |
| OTU0872 | Bacteria | Proteobacteria | Gammaproteobacteria | Xanthomonadales | Unclassified | Unclassified |
| OTU0873 | Bacteria | Patescibacteria | uncultured | Unclassified | Unclassified | Unclassified |
| OTU0874 | Bacteria | Actinobacteria | OPB41 | Unclassified | Unclassified | Unclassified |
| OTU0875 | Bacteria | Planctomycetes | Planctomycetacia | uncultured | Unclassified | Unclassified |
| OTU0876 | Bacteria | Kiritimatiellaeota | Kiritimatiellae | WCHB1-41 | Unclassified | Unclassified |
| OTU0877 | Bacteria | Proteobacteria | Gammaproteobacteria | WD260 | Unclassified | Unclassified |
| OTU0878 | Bacteria | Actinobacteria | Thermoleophilia | Gaiellales | Unclassified | Unclassified |
| OTU0879 | Bacteria | Proteobacteria | Alphaproteobacteria | DB1-14 | Unclassified | Unclassified |
| OTU0880 | Bacteria | Proteobacteria | Gammaproteobacteria | NKB5 | Unclassified | Unclassified |
| OTU0881 | Bacteria | Proteobacteria | Gammaproteobacteria | Alteromonadales | uncultured | Unclassified |
| OTU0882 | Bacteria | Chloroflexi | Ardenticatenia | Ardenticatenales | uncultured | Unclassified |
| OTU0883 | Bacteria | Proteobacteria | Campylobacteria | Campylobacterales | uncultured | Unclassified |
| OTU0884 | Bacteria | Bacteroidetes | Bacteroidia | Chitinophagales | uncultured | Unclassified |
| OTU0885 | Bacteria | Actinobacteria | Actinobacteria | Frankiales | uncultured | Unclassified |
| OTU0886 | Bacteria | Actinobacteria | Thermoleophilia | Gaiellales | uncultured | Unclassified |
| OTU0887 | Bacteria | Firmicutes | Limnochordia | Limnochordales | uncultured | Unclassified |
| OTU0888 | Bacteria | Proteobacteria | Alphaproteobacteria | Rhodospirillales | uncultured | Unclassified |

| | | | | | | |
|---|---|---|---|---|---|---|
| OTU0889 | Bacteria | Proteobacteria | Gammaproteobacteria | Gammaproteobacteria Incertae Sedis | Unknown Family | Acidibacter |
| OTU0890 | Bacteria | Proteobacteria | Gammaproteobacteria | Gammaproteobacteria Incertae Sedis | Unknown Family | Candidatus Berkiella |
| OTU0891 | Bacteria | Firmicutes | Negativicutes | Selenomonadales | Veillonellaceae | Negativicoccus |
| OTU0892 | Bacteria | Firmicutes | Negativicutes | Selenomonadales | Veillonellaceae | Veillonella |
| OTU0893 | Bacteria | Firmicutes | Negativicutes | Selenomonadales | Veillonellaceae | Anaeroglobus |
| OTU0894 | Bacteria | Firmicutes | Negativicutes | Selenomonadales | Veillonellaceae | Centipeda |
| OTU0895 | Bacteria | Firmicutes | Negativicutes | Selenomonadales | Veillonellaceae | Dialister |
| OTU0896 | Bacteria | Firmicutes | Negativicutes | Selenomonadales | Veillonellaceae | Megamonas |
| OTU0897 | Bacteria | Firmicutes | Negativicutes | Selenomonadales | Veillonellaceae | Megasphaera |
| OTU0898 | Bacteria | Firmicutes | Negativicutes | Selenomonadales | Veillonellaceae | Pelosinus |
| OTU0899 | Bacteria | Firmicutes | Negativicutes | Selenomonadales | Veillonellaceae | Selenomonas |
| OTU0900 | Bacteria | Firmicutes | Negativicutes | Selenomonadales | Veillonellaceae | Selenomonas 3 |
| OTU0901 | Bacteria | Firmicutes | Negativicutes | Selenomonadales | Veillonellaceae | Unclassified |
| OTU0902 | Bacteria | Dependentiae | Babeliae | Babeliales | Vermiphilaceae | Unclassified |
| OTU0903 | Bacteria | Verrucomicrobia | Verrucomicrobiae | Verrucomicrobiales | Verrucomicrobiaceae | Akkermansia |
| OTU0904 | Bacteria | Verrucomicrobia | Verrucomicrobiae | Verrucomicrobiales | Verrucomicrobiaceae | Luteolibacter |
| OTU0905 | Bacteria | Verrucomicrobia | Verrucomicrobiae | Verrucomicrobiales | Verrucomicrobiaceae | Prosthecobacter |
| OTU0906 | Bacteria | Verrucomicrobia | Verrucomicrobiae | Verrucomicrobiales | Verrucomicrobiaceae | uncultured |
| OTU0907 | Bacteria | Proteobacteria | Gammaproteobacteria | Vibrionales | Vibrionaceae | Photobacterium |
| OTU0908 | Bacteria | Proteobacteria | Gammaproteobacteria | Vibrionales | Vibrionaceae | Vibrio |
| OTU0909 | Bacteria | Bacteroidetes | Sphingobacteriia | Sphingobacteriales | WCHB1-69 | Unclassified |
| OTU0910 | Bacteria | Planctomycetes | Phycisphaerae | Tepidisphaerales | WD2101 soil group | Unclassified |
| OTU0911 | Bacteria | Bacteroidetes | Flavobacteriia | Flavobacteriales | Weeksellaceae | Bergeyella |
| OTU0912 | Bacteria | Bacteroidetes | Flavobacteriia | Flavobacteriales | Weeksellaceae | Chryseobacterium |
| OTU0913 | Bacteria | Bacteroidetes | Flavobacteriia | Flavobacteriales | Weeksellaceae | Empedobacter |
| OTU0914 | Bacteria | Bacteroidetes | Flavobacteriia | Flavobacteriales | Weeksellaceae | Unclassified |

| | | | | | | |
|---|---|---|---|---|---|---|
| OTU0915 | Bacteria | Proteobacteria | Gammaproteobacteria | Steroidobacterales | Woeseiaceae | Woeseia |
| OTU0916 | Bacteria | Proteobacteria | Alphaproteobacteria | Rhizobiales | Xanthobacteraceae | Labrys |
| OTU0917 | Bacteria | Proteobacteria | Alphaproteobacteria | Rhizobiales | Xanthobacteraceae | Ancylobacter |
| OTU0918 | Bacteria | Proteobacteria | Alphaproteobacteria | Rhizobiales | Xanthobacteraceae | Pseudolabrys |
| OTU0919 | Bacteria | Proteobacteria | Alphaproteobacteria | Rhizobiales | Xanthobacteraceae | Unclassified |
| OTU0920 | Bacteria | Proteobacteria | Alphaproteobacteria | Rhizobiales | Xanthobacteraceae | Xanthobacter |
| OTU0921 | Bacteria | Proteobacteria | Alphaproteobacteria | Rhizobiales | Xanthobacteraceae | uncultured |
| OTU0922 | Bacteria | Proteobacteria | Gammaproteobacteria | Xanthomonadales | Xanthomonadaceae | Dokdonella |
| OTU0923 | Bacteria | Proteobacteria | Gammaproteobacteria | Xanthomonadales | Xanthomonadaceae | Dyella |
| OTU0924 | Bacteria | Proteobacteria | Gammaproteobacteria | Xanthomonadales | Xanthomonadaceae | Luteibacter |
| OTU0925 | Bacteria | Proteobacteria | Gammaproteobacteria | Xanthomonadales | Xanthomonadaceae | Rhodanobacter |
| OTU0926 | Bacteria | Proteobacteria | Gammaproteobacteria | Xanthomonadales | Xanthomonadaceae | Rudaea |
| OTU0927 | Bacteria | Proteobacteria | Gammaproteobacteria | Xanthomonadales | Xanthomonadaceae | Unclassified |
| OTU0928 | Bacteria | Proteobacteria | Gammaproteobacteria | Xanthomonadales | Xanthomonadaceae | Wohlfahrtiimonas |
| OTU0929 | Bacteria | Proteobacteria | Gammaproteobacteria | Xanthomonadales | Xanthomonadaceae | Arenimonas |
| OTU0930 | Bacteria | Proteobacteria | Gammaproteobacteria | Xanthomonadales | Xanthomonadaceae | Luteimonas |
| OTU0931 | Bacteria | Proteobacteria | Gammaproteobacteria | Xanthomonadales | Xanthomonadaceae | Lysobacter |
| OTU0932 | Bacteria | Proteobacteria | Gammaproteobacteria | Xanthomonadales | Xanthomonadaceae | Pseudoxanthomonas |
| OTU0933 | Bacteria | Proteobacteria | Gammaproteobacteria | Xanthomonadales | Xanthomonadaceae | Stenotrophomonas |
| OTU0934 | Bacteria | Proteobacteria | Gammaproteobacteria | Xanthomonadales | Xanthomonadaceae | Thermomonas |
| OTU0935 | Bacteria | Proteobacteria | Gammaproteobacteria | Xanthomonadales | Xanthomonadaceae | SN8 |
| OTU0936 | Bacteria | Proteobacteria | Gammaproteobacteria | Xanthomonadales | Xanthomonadaceae | Xanthomonas |
| OTU0937 | Bacteria | Verrucomicrobia | Spartobacteria | Chthoniobacterales | Xiphinematobacteraceae | Candidatus_Xiphinematobacter |
